# Photoelectrocatalytic C–H halogenation over an oxygen vacancy-rich TiO_2_ photoanode

**DOI:** 10.1038/s41467-021-26997-z

**Published:** 2021-11-18

**Authors:** Zhenhua Li, Lan Luo, Min Li, Wangsong Chen, Yuguang Liu, Jiangrong Yang, Si-Min Xu, Hua Zhou, Lina Ma, Ming Xu, Xianggui Kong, Haohong Duan

**Affiliations:** 1 State Key Laboratory of Chemical Resource Engineering, Beijing University of Chemical Technology, Beijing, 100029 China; 2 Department of Chemistry, Tsinghua University, 30 Shuangqing Rd, Beijing, 100084 China

**Keywords:** Nanoscale materials, Photocatalysis, Photocatalysis

## Abstract

Photoelectrochemical cells are emerging as powerful tools for organic synthesis. However, they have rarely been explored for C–H halogenation to produce organic halides of industrial and medicinal importance. Here we report a photoelectrocatalytic strategy for C–H halogenation using an oxygen-vacancy-rich TiO_2_ photoanode with NaX (X=Cl^−^, Br^−^, I^−^). Under illumination, the photogenerated holes in TiO_2_ oxidize the halide ions to corresponding radicals or X_2_, which then react with the substrates to yield organic halides. The PEC C–H halogenation strategy exhibits broad substrate scope, including arenes, heteroarenes, nonpolar cycloalkanes, and aliphatic hydrocarbons. Experimental and theoretical data reveal that the oxygen vacancy on TiO_2_ facilitates the photo-induced carriers separation efficiency and more importantly, promotes halide ions adsorption with intermediary strength and hence increases the activity. Moreover, we designed a self-powered PEC system and directly utilised seawater as both the electrolyte and chloride ions source, attaining chlorocyclohexane productivity of 412 µmol h^−1^ coupled with H_2_ productivity of 9.2 mL h^−1^, thus achieving a promising way to use solar for upcycling halogen in ocean resource into valuable organic halides.

## Abstract (web summary)

Photoelectrochemical cells are promising tools for C–H functionalisation coupled with H2 production. In this work, Duan et. al., reported the photoelectrocatalytic C–H halogenation to produce organic halides of industrial and medicinal importance with promoted H_2_ production.

## Introduction

**Fig. 1:**
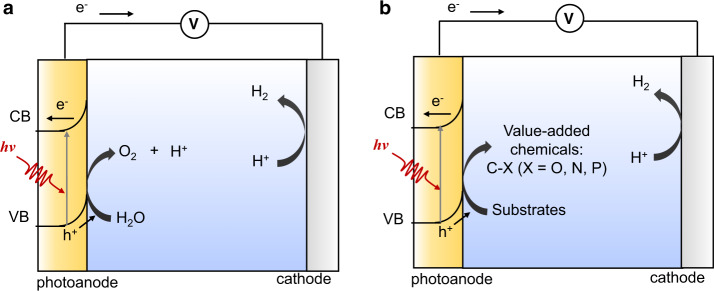
PEC cells. **a** PEC water oxidation coupled with H_2_ production. **b** PEC C–H functionalization coupled with H_2_ production. CB conduction band, VB valence band.

Photoelectrochemical (PEC) technology is regarded as an environmentally benign route for H_2_ production via water splitting. In a typical PEC cell for water splitting, the photoexcited electrons from the photoanode are transferred to the photocathode to drive H_2_O reduction to produce H_2_, and the photogenerated holes left on the photoanode are employed for the oxidation of H_2_O to O_2_ (Fig. 1a)^1,2^. However, the reaction efficiency is always hindered by the energetically and kinetically demanding O_2_ evolution reaction (OER)^3,4^. In recent years, PEC works have circumvented this limitation by introducing organic molecules oxidation reactions which are energetically more favorable than OER, making it an attractive strategy that promotes H_2_ production rate and simultaneously produces value-added chemicals from cheap organic compounds^5,6^. However, the reaction variety is limited to the oxidation of simple alcohol substrates and oxygenated biomass feedstocks^7–11^ such as 5-Hydroxymethylfurfural (HMF)^7,8^ and benzyl alcohol^11^. Recently, significant developments have been made on C–H functionalization in PEC cell system, achieving the construction of carbon–heteroatom bonds to produce high value-added chemicals over the photoanode at mild conditions (Fig. 1b)^12–14^. For instance, Wu and co-workers successfully realized the P–H/C–H cross-coupling to construct C–P bond via a PEC process, which showed good functional-group tolerance, broad substrate scope and high yield^12^. Hu and co-workers creatively developed an efficient PEC strategy to achieve non-directed arene C–H animation with high *ortho* selectivity over a hematite photoanode^13^. Despite these progresses, there is only a handful of carbon–heteroatom bonds that can be constructed in PEC cell system, involving C–N, C–O, and C–P bonds^14^. Therefore, the development of efficient PEC strategy for C–H functionalization to construct more types of carbon–heteroatom bonds is yet to be explored and highly desirable.

**Fig. 2:**
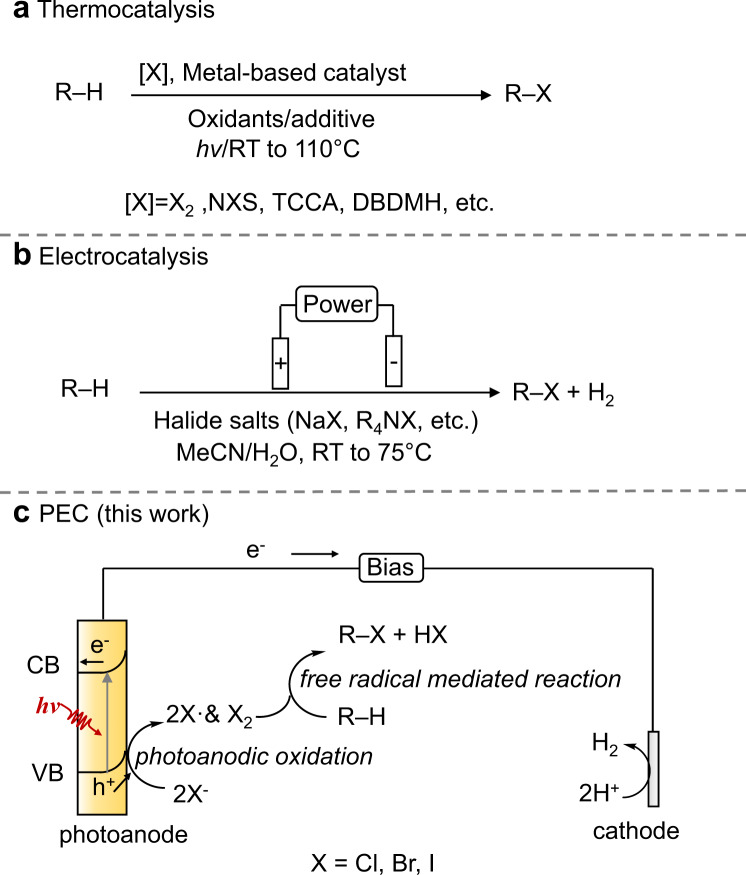
C–H halogenation. **a** Thermocatalytic process. **b** Electrocatalytic process. **c** Photoelectrocatalytic process (this work).

Organic halides are a family of important fine chemicals that have wide applications in various branches of chemistry (e.g., medicines, dyestuffs, agrochemicals, etc), as well as in electronic and metalworking industries^15^. The conventional synthesis methods involve thermal homogeneous or heterogeneous catalytic processes (Fig. 2a)^16–23^. Although efficient and well-reported in literatures, these methods have limitations that hinder the production in a sustainable manner, such as the use of hazardous and expensive halogenation reagents (such as dihalogen or *N*-halogensuccinimide)^16,17^, the need of metal salts as the catalysts (e.g., CoX_2_, CuX_2_, LiX, etc) that are difficult to be separated and reused^18,19^, the need of external oxidants (e.g., O_2_, 2KHSO_5_·KHSO_4_·K_2_SO_4_, AgSbF_6_, etc)^20,21^ and elevated temperature^22,23^. Very recently, Lei and co-workers reported an electrochemical oxidation method for C–H halogenation with HX/NaX, showing broad substrate scope and high efficiency^24^ (Fig. 2b). Nevertheless, this process still needs large external bias (>2.5 V vs. RHE), and organic solvent (e.g., MeCN, DMF) and electrolyte (e.g., ^*n*^Bu_4_NBF_4_) are required for optimum reaction conditions. Compared with electrocatalytic method, photoelectrocatalytic method is expected to reduce the applied external bias, therefore saving electricity and potentially suppressing side reactions that may proceed under high applied voltage. It is reported that halogenation reaction is a typical free radical-mediated process, in which the halogen radicals react with the substrates containing C–H bonds to yield organic halides^16,18,25^. Thus, we speculate that halogenation reaction can proceed in the PEC cell with halogen ions if the photogenerated holes in the valence band (VB) of photoanodes is more positive than the oxidation potential of the halogen ions to halogen radicals (Fig. 2c). However, direct C–H halogenation has never been explored in PEC cells.

In this work, we report a photoelectrocatalytic strategy for C–H halogenation with NaX (X=Cl^−^, Br^−^, I^−^) over an oxygen-vacancy-rich TiO_2_ photoanode, producing high value-added organic halides coupled with cathodic H_2_ production (Fig. 2c). The NaX aqueous solution (pH = 7) is used as both the electrolyte and halide source. Specifically, we achieve monochlorination of cyclohexane to chlorocyclohexane in 92.5% selectivity with yield of 0.064 mmol cm^−2^ h^−1^. Experimental integrated with spin-polarized density functional theory (DFT) studies reveal that the PEC chlorination of cyclohexane mainly follows a free radical chain reaction process, in which the chlorine radicals and Cl_2_ are generated through the activation of halogen ions catalyzed by the photogeneated holes over TiO_2_. The strategy exhibits broad substrate scope for arenes, heteroarenes, nonpolar cycloalkanes, and aliphatic hydrocarbons. Moreover, both experimental and theoretical results indicate that the oxygen vacancies on TiO_2_ help enhance the adsorption of halogen ion and thus their enrichment in local environment, together with the enhanced photo-induced carriers separation efficiency. Finally, we design a self-powered PEC system and directly utilized seawater as the electrolyte and the Cl^−^ source, attaining chlorocyclohexane productivity of 412 µmol h^−1^ coupled with H_2_ productivity of 9.2 mL h^−1^. As a result, we achieve a promising way to use solar for upcycling halogen in ocean resource into valuable organic halides.

## Results

### Screening and optimization of photoanodes

**Fig. 3:**
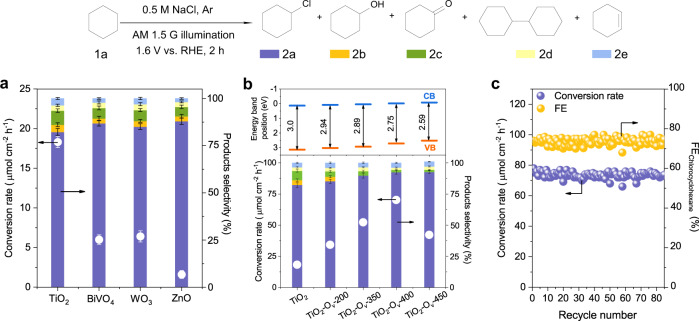
Cyclohexane chlorination performance via PEC. **a** Photoelectrocatalytic conversion rate of cyclohexane and corresponding products selectivity over typical semiconductors. **b** Photoelectrocatalytic conversion rate of cyclohexane and corresponding products selectivity over TiO_2_-O_v_-*T* (*T* represents the temperatures, 200, 350, 400, and 450 °C) photoanodes. **c** Stability test of TiO_2_-O_v_-400 photoanode for the chlorination of cyclohexane for 255 h in batch reaction (85 batches). The reaction was carried out in a batch reaction in 0.5 M NaCl with 18.8 mmol cyclohexane at 1.6 V vs. RHE under AM 1.5G, 100 mW cm^−2^ illumination, with 3 h for one batch. The spent TiO_2_-O_v_-400 photoanode was washed by deionized water and dried under vacuum for the next batch reaction. The error bars represent the relative deviation, which is within 5%. The curves in each figure are guides to the eye.

Cyclohexane was selected as the model substrate for PEC chlorination reaction. The PEC tests were performed in a three-electrode configuration in a H-type quartz cell, and Pt foil was used as the cathode and Ag/AgCl electrode as the reference electrode. A NaCl aqueous solution (0.5 M, pH = 7) was used as both the electrolyte and chlorine source. Several typical semiconductors (TiO_2_, WO_3_, BiVO_4_, and ZnO) were synthesised and screened as the photoanode (the synthesis methods and structural characterisations see experimental section and Supplementary Fig. 1). Linear sweep voltammetry (LSV) curves in Supplementary Fig. 2 show that TiO_2_ exhibits higher photocurrent density compared to other semiconductors in a 0.5 M NaCl electrolyte containing 18.8 mmol cyclohexane under AM 1.5G irradiation (that is 100 mW cm^−2^), and achieves higher cyclohexane conversion rate of 16 µmol cm^−2^ h^−1^ at 1.6 V vs. RHE (Fig. 3a). The gas chromatography-mass spectrometry (GC-MS, Supplementary Fig. 3 and Supplementary Table 1) show that chlorocyclohexane (2a) is the main product over different photoanodes with selectivity in the range of 82.3–88.0% and FE of 56.8–68.5%. This indicates the efficacy of PEC strategy for chlorination reaction, although the selectivity of chlorocyclohexane requires further improvement. We notice that the byproducts include cyclohexanol (2b), cyclohexanone (2c), bicyclohexyl (2d) and cyclohexene (2e) with much lower selectivity. Among them, cyclohexanol and cyclohexanone are likely attributed to the oxidation of cyclohexane by hydroxyl radicals from H_2_O^26,27^, cyclohexene is due to the dehydration of cyclohexanol, and bicyclohexyl may come from the recombination of cyclohexane radical^18^. Due to the higher catalytic performance of TiO_2_ in PEC chlorination of cyclohexane, we focus on it for further study.

Previous reports reveal that introducing oxygen vacancy can effectively enhance the photo-induced charge separation and visible light absorption performances for TiO_2_ photoanode^28^. We thus explored the effect of oxygen vacancy on PEC chlorination reaction over the TiO_2_ photoanode. The oxygen vacancy was generated through treating the pristine TiO_2_ photoanode with H_2_ under different temperatures. The treated samples are denoted as TiO_2_-O_v_-*T* (*T* represents the temperatures, 200, 350, 400, and 450 °C were used; Supplementary Fig. 4). The formation of oxygen vacancy was identified by X-ray photoelectron spectroscopy (XPS; Supplementary Fig. 5), in which the O *1s* XPS spectra of all the TiO_2_-O_v_-*T* samples show a typical oxygen adsorption peak at the bonding energy of 531.4 eV, which are assigned to the oxygen vacancy^28,29^. The corresponding peak intensity was enhanced with the increase of H_2_ treatment temperature, an indicative of the increased oxygen vacancy concentration (Supplementary Table 2), consistent with the previous reports^29^. The as-prepared TiO_2_-O_v_-*T* samples were then tested for PEC chlorination of cyclohexane. As shown in Supplementary Figs. 6 and 7, the pristine TiO_2_ photoanode exhibits a bandgap of 3.0 eV with an absorption wavelength of solar spectrum < 400 nm. This causes a relatively low photocurrent density of 1.5 mA cm^−2^ at 1.6 V vs. RHE in 0.5 M NaCl with 18.8 mmol cyclohexane under AM 1.5G illumination (100 mW cm^−2^). After introducing oxygen vacancy, the photocurrent density increases from 1.5 mA cm^−2^ (pristine TiO_2_) to 3.4 mA cm^−2^ (TiO_2_-O_v_-400), suggesting the vital role of the oxygen vacancy for the PEC reaction activity. This can be partly attributed to the narrower bandgap of TiO_2_-O_v_-400 (2.75 eV; Fig. 3b and Supplementary Fig. 7) that facilitates photo-induced charge separation, suppresses electron-hole recombination, and also expands the light absorption range to visible light (from 410 nm to 450 nm). Noted that further increasing the H_2_ treatment temperature (TiO_2_-O_v_-450) resulted in lower photocurrent density, which can be rationalized that the oxygen vacancy might induce photogenerated carriers recombination at higher concentration^30^.

As a result, the TiO_2_-O_v_-400 exhibits better catalytic performance compared to other TiO_2_ samples, achieving higher cyclohexane conversion rate, relatively higher selectivity (92.5%) and FE (77.5%) of chlorocyclohexane, and relatively lower FE of OER (7.3%) (Fig. 3b, Supplementary Fig. 8 and Supplementary Table 3). For the selectivity and FE calculations, all the organic products and evolved O_2_ were included. The photon-to-current conversion efficiencies (IPCE) and photon-to-chlorocyclohexane quantum efficiency (QE) achieve 89.2% and 64.2% at a wavelength of 370 nm over TiO_2_-O_v_-400 (Supplementary Fig. 9), both higher than that of TiO_2_ samples. The optimized PEC chlorination condition is presented as 0.5 M NaCl solution at 1.6 V vs. RHE under AM 1.5G illumination for 2 h (Supplementary Fig. 10), which was adopted for further catalytic reactions unless specified. The kinetic curves in Supplementary Fig. 11 show that chlorocyclohexane is the main product when the conversion of cyclohexane is lower than 60%. At this stage, cyclohexane underwent rapid oxidation, giving >50% yield of chlorocyclohexane with FE > 72%. However, chlorocyclohexane was partly converted to dichlorocyclohexane at higher cyclohexane conversion, and the conversion rate of cyclohexane gradually decreased, which is due to the involvement of highly reactive chlorine radicals that react with the accumulated chlorocyclohexane. Finally, we achieved a maximum chlorocyclohexane yield of 56.3% when the conversion of cyclohexane reached 63.1%. By using the PEC strategy, the organic halogenation reaction can be conducted in an energy-saving way by harnessing solar energy. To demonstrate it, we constructed an EC cell composed of a glass carbon anode, a Pt plate counter electrode and an Ag/AgCl reference electrode for the cyclohexane chlorination^12^ (Supplementary Fig. 12). The results show that the applied bias to produce a (photo)-current of 2 mA cm^−2^ is 0.7 V vs. RHE for the TiO_2_-O_v_-400 PEC system, which is much lower than that of 2.4 V vs. RHE for the glassy carbon EC system. These potentials afford very similar cyclohexane conversion rates (41.2 µmol cm^−2^ h^−1^ in the PEC cell and 36.5 µmol cm^−2^ h^−1^ in the EC cell) and chlorocyclohexane selectivity (93% in the PEC cell and 90.7% in the EC cell) after 2 h of reaction, indicating the potential of energy-saving process for PEC organic synthesis.

For the H_2_ production performance at the counterpart Pt cathode, the space-time yield reaches 1.57 mL cm^−2^ h^−1^ over TiO_2_-O_v_-400, which is much higher than that of the pristine TiO_2_ (0.66 mL cm^−2^ h^−1^; Supplementary Fig. 13). Both TiO_2_-O_v_-400 and pristine TiO_2_ exhibit high Faradaic efficiency of H_2_ (FE > 99%). We also evaluated the stability of the TiO_2_ and TiO_2_-O_v_-400 photoanodes in PEC cyclohexane chlorination in batch reactions. As shown in Fig. 3c and Supplementary Fig. 14, after 85 batches (overall 255 h), the conversion rate (from 70.0 to 69.2 µmol cm^−2^ h^−1^) and the FE of chlorocyclohexane (from 77.5 to 74.3%) remain stable, demonstrating the stability of the TiO_2_-O_v_-400 photoanode.

### Characterisations and photoelectric properties of the optimal TiO_2_-O_v_-400

**Fig. 4:**
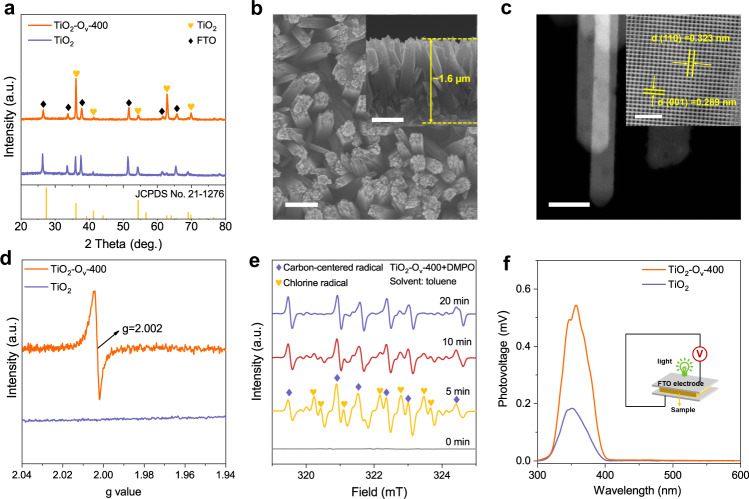
Structural characterisations and photoelectric properties of TiO_2_-O_v_-400 photoanode. **a** XRD patterns of TiO_2_ and TiO_2_-O_v_-400. **b** SEM images of TiO_2_-O_v_-400. Scale bars: 200 nm (inset). **c** HAADF-STEM images of the TiO_2_-O_v_-400. Scale bars: 200 nm, 2 nm (inset). **d** Room temperature EPR spectra of TiO_2_ and TiO_2_-O_v_-400. **e** EPR spectrum detected from the photolysis of TiO_2_-O_v_-400 after immersing in 0.5 M NaCl under AM 1.5G (100 mW cm^−2^) illumination in toluene solution containing DMPO as spin-trapping agent. **f** Steady-state SPV spectra of TiO_2_ and TiO_2_-O_v_-400 photoanodes. The inset shows the schematic of the photovoltaic cell structure. a.u.: arbitrary units.

The optimal TiO_2_-O_v_-400 photocatalyst was then characterised to understand the catalytic performance. X-ray diffraction (XRD) shows that TiO_2_-O_v_-400 displays a typical rutile phase of TiO_2_ (Fig. 4a)^32^. Scanning electron microscope (SEM) image reveals the formation of nanorod array structure with average diameter of 200–300 nm and length of ∼1.6 μm (Fig. 4b). High-angle annular dark-field scanning transmission electron microscope (HAADF-STEM) images show that the nanorods are well crystallized with fringe distances of 0.323 nm and 0.289 nm (Fig. 4c), closely matching the (110) and (001) plane of TiO_2_. The TiO_2_-O_v_-400 sample exhibits a strong electron paramagnetic resonance (EPR) signal at *g* value of 2.002 (Fig. 4d), which can be attributed to the electrons trapped in oxygen vacancies^33^. Together with the XPS results shown above, these results further confirm the formation of oxygen vacancy in TiO_2_-O_v_-400.

We then performed in-situ EPR and Cl_2_ experiments to reveal the reaction mechanism of PEC chlorination of cyclohexane over TiO_2_-O_v_-400 photocatalyst. The EPR measurement was conducted in toluene by using 5,5-dimethyl-1-pyrroline N-oxide (DMPO) as the trapping agent^34,35^. As shown in Fig. 4e, both chlorine radical and carbon-centered radical were observed in the EPR spectrum after 5 min irradiation. After irradiation for 20 min, chlorine radical disappeared and carbon-centered radical remained in the system. This may be due to the quenching of chlorine radicals after it reacted with the C–H bond to yield carbon-centered radicals. Since chlorocyclohexane is the main product rather than C–C coupling products, the reaction mechanism should not follow a radical-radical combination process, but probably follow the free radical chain reaction. To further demonstrate the reaction mechanism, we used Cl_2_ as the chlorine source for halogenation of cyclohexane. Cl_2_ was injected into an aqueous solution containing 5 mmol cyclohexane in the presence of TiO_2_-O_v_ or pure TiO_2_ or without catalyst, and the reaction was performed under light irradiation or under darkness for 15 min. As shown in Supplementary Fig. 15a, under light irradiation, chlorocyclohexane is the only product for all the three reactions, and there are negligible products for reaction under darkness while other conditions were kept the same (Supplementary Fig. 15b). Cl· was generated via the homolytic cleavage of Cl_2_ under light irradiation, which follows a free-radical chain mechanism for cyclohexane chlorination^36^. As a result, the PEC halogenation of cyclohexane in our work probably follows a similar free-radical mechanism, except that Cl_2_ was produced by direct oxidation of Cl^−^ catalyzed by the photogenerated holes (h^+^) over photoanode.

To understand the high photoelectrocatalytic activity of the TiO_2_-O_v_-400 photoanode, its intrinsic photoelectric properties of TiO_2_-O_v_-400 were studied. Surface photovoltage (SPV) spectra provides a direct probe of the photophysics within semiconductor materials^37^. As shown in Fig. 4f, the TiO_2_-O_v_-400 exhibits an obvious steady-state SPV signal under light irradiation. The SPV signal decays with the increasement of excitation light wavelength, which corresponds to the excitation of oxygen vacancy. Clearly, introducing oxygen vacancy enhances the SPV response of TiO_2_, which demonstrates efficient photogenerated charge carrier separation efficiency. The photoluminescence (PL) emission spectra can be used to evaluate the recombination of photoexcited electrons/holes in TiO_2_, which shows the maximum broadbands around 470 nm^33^. As shown in Supplementary Fig. 16, the TiO_2_-O_v_-400 sample exhibits lower intensity compared to the pristine TiO_2_ and other TiO_2_-O_v_ samples, demonstrating a more efficient separation of the photo-induced carriers. Moreover, the electrochemical impedance spectroscopy (EIS) plots present a faster kinetics of charge transfer at the interface of TiO_2_-O_v_-400 and the electrolyte (Supplementary Fig. 17), proving an efficient separation of the photo-induced carriers^38^.

### Effect of oxygen vacancy in promoting chloride ions oxidation

**Fig. 5:**
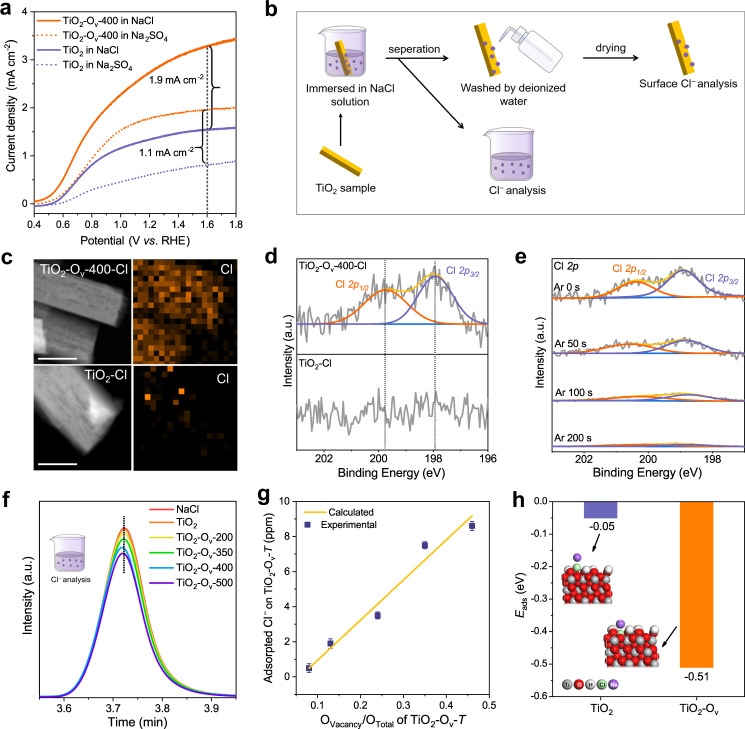
Mechanistic investigation of enriched Cl^−^ at oxygen-vacancy sites. **a** LSV curves at scan rate of 10 mV s^−1^ in 0.5 M Na_2_SO_4_ or NaCl electrolyte under AM 1.5G (100 mW cm^−2^) illumination. **b** Schematic diagram of the pretreatment process of TiO_2_-O_v_-400 and TiO_2_ samples for further measurements (denoted as TiO_2_-O_v_-400-Cl and TiO_2_-Cl). **c** STEM combined EDS mapping images of TiO_2_-O_v_-400-Cl and TiO_2_-Cl. Scale bars: 100 nm. **d** Cl *2p* XPS spectra of TiO_2_-O_v_-400-Cl and TiO_2_-Cl. **e** ARXPS spectra of Cl on TiO_2_-O_v_-400-Cl after Ar^+^ sputtering. **f** IC spectrogram of NaCl solution after adsorbed by different TiO_2_-O_v_-*T* samples. **g** Cl^−^ adsorption behavior of TiO_2_-O_v_-400 with different concentration of oxygen vacancy. **h** Adsorption energy of Cl^−^ over TiO_2_ and TiO_2_-O_v_. Inset: optimized geometries for TiO_2_ and TiO_2_-O_v_ adsorbed with Cl^−^, the color of each element is also labeled. a.u.: arbitrary units.

We observed that TiO_2_-O_v_-400 shows higher photocurrent than pristine TiO_2_ in a 0.5 M Na_2_SO_4_ aqueous solution (dashed line, Fig. 5a), indicating that TiO_2_ with oxygen vacancy can also promote the activity of oxidation of H_2_O. However, the photocurrent increments for oxidation of Cl^−^ is higher than oxidation of H_2_O. For the oxidation of Cl^−^ at 1.6 V vs. RHE, the photocurrent was increased from 1.5 mA cm^−2^ (pristine TiO_2_) to 3.4 mA cm^−2^ (TiO_2_-O_v_-400), that equals to 1.9 mA cm^−2^ enhancement. In contrast, for the oxidation of H_2_O at 1.6 V vs. RHE, the photocurrent was increased from 0.8 mA cm^−2^ (pristine TiO_2_) to 1.9 mA cm^−2^ (TiO_2_-O_v_-400), showing 1.1 mA cm^−2^ enhancement. The inhibition of water oxidation of TiO_2_-O_v_-400 in 0.5 M NaCl was confirmed by online monitoring the O_2_ generation rate and FE. As shown in Supplementary Fig. 18, the FE of O_2_ is <10% over TiO_2_-O_v_-400, lower than that over TiO_2_ (~22%) under the conditions (in a 0.5 M NaCl solution after 1 h of reaction). These results indicate that the oxygen vacancy not only facilitates the photo-induced carriers separation efficiency over TiO_2_, but also has additional promoting effect for Cl^−^ oxidation.

We consider that it worth investigating the interaction between the TiO_2_ with oxygen vacancy and Cl^−^. It is assumed that the Cl^−^ upon adsorption would be difficult to remove by moderate washing treatment and retain on the TiO_2_ surface if a strong interaction exists between them, then the remained Cl^−^ can be detected. For this consideration, as illustrated in Fig. 5b, TiO_2_-O_v_-400 and pristine TiO_2_ were firstly immersed in a 0.5 M NaCl solution under continuous stirring for 1 h to fully adsorb Cl^−^. Then, they were washed by deionized water and dried under vacuum (denoted as TiO_2_-O_v_-400-Cl and TiO_2_-Cl, respectively) for further analysis. The energy-dispersive X-ray spectroscopy (EDS) mapping results in Fig. 5c and Supplementary Fig. 19 reveal that Cl species are uniformly distributed on the surface of the TiO_2_-O_v_-400-Cl with mass loading of ~0.67 wt% (Supplementary Table 4). In contrast, there is no obvious Cl on the surface of the TiO_2_-Cl. XPS measurement (Fig. 5d) show that TiO_2_-O_v_-400-Cl exhibits a set of typical Cl *2p* XPS peaks at 198.1 eV (Cl *2p*_*3/2*_) and at 199.8 eV (Cl *2p*_*1/2*_)^39^, while they are absent in the TiO_2_-Cl sample. These results suggest that TiO_2_-O_v_-400 adsorbs Cl^−^ stronger than the pristine TiO_2_ that is due to the oxygen vacancy. To investigate the location of the adsorbed Cl over the TiO_2_-O_v_-400-Cl, namely on the surface or in the bulk, angle-resolved XPS (ARXPS) was conducted. The results show that the peak intensities of Cl *2p* on the TiO_2_-O_v_-400 became less intensive and finally disappeared with the extension of Ar^+^ sputtering time (from 0 to 200 s; Fig. 5e), indicating that Cl^−^ is mainly adsorbed on the surface of TiO_2_-O_v_-400. We then estimated the adsorption quantity of Cl^−^ by immersing TiO_2_-O_v_-*T* samples in a NaCl aqueous solution and then measuring the Cl^−^ concentration in the NaCl solution using ion chromatography (IC; Fig. 5f, Supplementary Fig. 20 and Supplementary Table 5). Based on the measurement results, the adsorbed amount of Cl^−^ has a positive correlation with the concentration of oxygen vacancy on TiO_2_-O_v_-*T* (Fig. 5g), demonstrating that the oxygen vacancy on TiO_2_ has a pivotal role in Cl^−^ adsorption and may enrich Cl^−^ in local environment on TiO_2_ surface for enhanced catalytic performance.

### Spin-polarized density functional theory studies

Spin-polarized density functional theory (DFT) calculations were performed to reveal the effect of oxygen vacancy on the adsorption of Cl^–^, and its further oxidation to Cl· and Cl_2_ on TiO_2_-O_v_, together with the chlorination of cyclohexane by Cl· and Cl_2_ in solvent. Detailed information for model construction and computational methods are presented in the Methods section. According to the HAADF-STEM results (Fig. 4c), the exposed facet of TiO_2_-O_V_ is determined to be the (101) facet, which has been reported to be one of the preferably exposed facets of rutile TiO_2_^40^. By calculating the Gibbs free energies of TiO_2_ (101) facets terminated with Ti, –H, –OH, and –O species, it is found that the (101) facet terminated with –OH is more stable than the others (Supplementary Figs. 21 and 22, and Supplementary Note 1). Therefore, the TiO_2_ (101) facet terminated with –OH was employed in the following calculations. As shown in Fig. 5h, the Cl^‒^ is adsorbed on the oxygen vacancy of TiO_2_-O_v_ with a moderate adsorption energy of –0.51 eV, resulting from the orbital overlap between the occupied Cl-3*p* orbital in Cl^‒^ and unoccupied Ti-3*d* orbital. In contrast, the adsorption of Cl^‒^ on TiO_2_ is much weaker than that on TiO_2_-O_v_ because of the repulsion between the lone pair electrons of Cl^‒^ and that of bridged O in TiO_2_, resulting in the adsorption energy of –0.05 eV (Fig. 5h and Supplementary Fig. 23). These theoretical results well explain the moderate adsorption of Cl^‒^ on TiO_2_-O_v_-400 photoanode according to the experimental results (Fig. 5c–g). According to the Sabatier rule^41^, too weak adsorption of reactant may lead to a slow reaction rate, as in the case with TiO_2_. While TiO_2_-O_v_ possesses an intermediary adsorption strength to Cl^–^, facilitating the adsorption of Cl^–^, generation and desorption of Cl*.

**Fig. 6:**
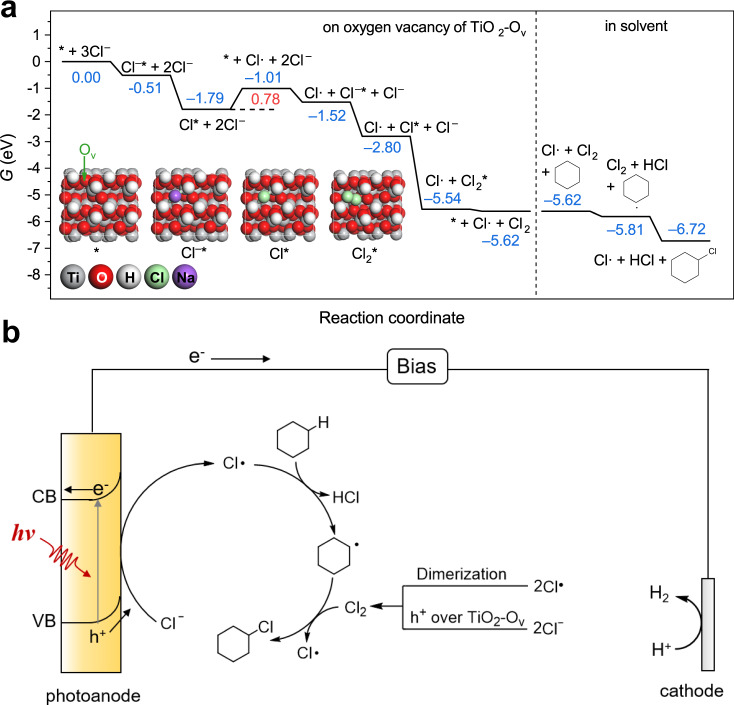
DFT calculations of the PEC cyclohexane chlorination and proposed mechanism. **a** Gibbs free energy diagram for the adsorption of Cl^–^, generation of Cl* and Cl_2_*, and their further desorption to Cl· and Cl_2_ on TiO_2_-O_v_, together with the chlorination of cyclohexane by Cl· and Cl_2_. The Gibbs free energies (Δ*G*) of reactant, intermediates, and product are labeled with blue numbers in brackets with the unit of eV. The Δ*G* of the potential determining step, desorption of Cl*, is labeled in red number with the unit of eV. The top views for the reaction intermediates, * (the reaction site of oxygen vacancy is highlighted with green circle), Cl^–^*, Cl*, and Cl_2_*, are also displayed with the color of each element labeled. **b** Proposed mechanism of PEC chlorination of cyclohexane over TiO_2_-O_v_-400 photoanode.

Furthermore, the Gibbs free energy diagrams of the whole process for the chlorination of cyclohexane over TiO_2_-O_v_ were calculated. As shown in Fig. 6a, after the adsorption of Cl^–^ on TiO_2_-O_v_, the Cl^–^ is oxidized by the hole of TiO_2_-O_v_ spontaneously with a Gibbs free energy change (Δ*G*) of –1.28 eV. The potential determining step is the desorption of Cl* to Cl· with a Δ*G* of 0.78 eV, which can be overcome by the kinetic energy of Cl* at room temperature. After the recovery of the active site, another Cl^–^ is adsorbed on the oxygen vacancy, which is followed by the oxidation of Cl^–^ to Cl*. After that, another Cl^–^ is oxidized by the hole on Cl*, generating Cl_2_* exothermically with a Δ*G* of –2.73 eV. Then the Cl_2_ desorbs spontaneously from the oxygen vacancy of TiO_2_-O_v_ with a Δ*G* of –0.08 eV. Noted that Cl_2_ may be also formed by dimerization of two Cl· that desorbed from the surface of TiO_2_-O_v_. After that, the generated Cl· and Cl_2_ chlorinate the cyclohexane in solvent. Firstly, the Cl· reacts with the C‒H bond in cyclohexane to generate carbon-centered radical exothermically with a Δ*G* of –0.20 eV. Then, the generated carbon-centered radical reacts with the Cl_2_ to give chlorocyclohexane and release the Cl· for the next cycle spontaneously with a Δ*G* of –0.91 eV. The proposed mechanism of PEC cyclohexane chlorination is presented in Fig. 6b: i. chlorine radicals (Cl·) are generated through the activation of Cl^–^ by the photogenerated holes (h^+^) over TiO_2_-O_v_-400 or TiO_2_ photoanode via single electron transfer; ii. the generated Cl· then activates the C–H bonds of cyclohexane to form cyclohexane radical. Cl_2_ can be generated by dimerization of two Cl· or by oxidation of two Cl^−^ catalyzed by the photogenerated holes (h^+^) over TiO_2_; iii. the generated cyclohexane radical reacts with Cl_2_ to form chlorocyclohexane and release the Cl· for the next cycle. The chlorocyclohexane can be further chlorinated to form dichlorocyclohexane following the same free-radical mechanism.

### Scope of photoelectrocatalytic C–H halogenation

To demonstrate the general applicability of the photoelectrocatalytic strategy for C–H halogenation over the TiO_2_-O_v_-400 photoanode, a broad scope of substrates was tested, including arenes, heteroarenes, nonpolar cycloalkanes, and aliphatic hydrocarbons. As shown in Table 1, for aromatic compounds with strong electron-donating groups (e.g., anisole and phenol; 1 and 2), di-chlorinated products were mainly obtained under high conversion (>90%), with selectivity of 52.6% for 2,4-dichloro-1-methoxybenzene (1) and 51.5% for 2,4-dichlorophenol (2) (Supplementary Figs. 24 and 25). For aromatic compounds with strong electron-withdrawing halogen groups (3–5), mono-chlorinated products were mostly obtained with the regioselectivities (the ratio of *para*- to*ortho-*, *p*/*o*) approximately of 1:0.7 to 1:1 (Supplementary Figs. 26, 27, and 28). It should be noted that for aromatic compounds with methyl group (e.g., toluene and methylnaphthalene; 6 and 7), aromatic ring-chlorinated compounds were the major products rather than the methyl-chlorinated ones (Supplementary Figs. 29 and 30). This is due to the PEC chlorination of toluene and methylnaphthalene over TiO_2_-O_v_-400 photoanode mainly follows an electrophilic substitution mechanism, which was demonstrated by the chlorine gas (Cl_2_) experiment (Supplementary Figs. 31–33; please see the detailed discussion in Supplementary Note 2 in the Supplementary information). For aromatic substrate with singular reactive site (vanillin, 8), high 5-chlorovanillin selectivity (>99%) were achieved under high conversion (94.2%) (Supplementary Fig. 34). Moreover, the TiO_2_-O_v_-400 photoanode can realize the PEC chlorination of heteroarenes (including thiophene, pyrazole, indoles, pyridine, furan, and imidazole-based substrates; 9–15), affording mono- or di-chlorinated products under high conversion (Supplementary Figs. 35–41). We then attempted to extend the substrate to drug-related compound, anesthetic procaine (16), and high selectivity of chloroprocaine was obtained, although the conversion (23.2%) was not improved significantly as the reaction time were prolonged, which is attributed to the steric hindrance of the benzene ring with large function group (Supplementary Fig. 42).

**Table 1:**
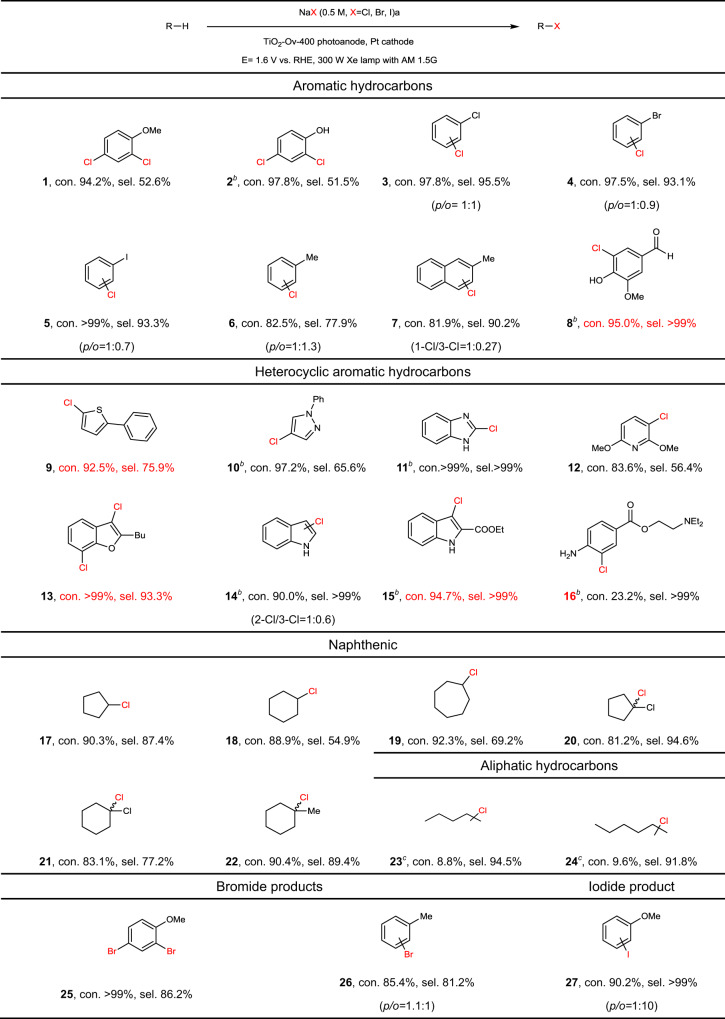
Scope of PEC C−H halogenation over TiO_2_-O_v_-400 photoanode.

^a^General conditions: substrate (0.1 mmol) in NaX (0.5 M, 10 mL) at ambient temperature under AM 1.5 G, 100 mW cm^−2^ illumination, Ar, 10 h. Applied potential, *E* = 1.6 V vs. RHE, conversion and selectivity. Other products are shown in Supplementary Figs. 24–53.

^b^General conditions: substrate (0.1 mmol) dissolved in 0.5 mL acetonitrile, NaCl (0.5 M, 10 ml) at ambient temperature under AM 1.5 G, 100 mW cm^−2^ illumination, Ar, 10 h. Applied potential, *E* = 1.6 V vs. RHE, conversion and selectivity. Other products are shown in Supplementary Figs. 24–53.

^c^Products obtained at low conversion.

For nonpolar cycloalkanes (17–19), mono-chlorinated products were the main products under high conversion (Supplementary Figs. 43–45). The chlorination of cycloalkanes with chlorine group (20 and 21) and methyl group (22) took place in the tertiary C − H bond position (Supplementary Figs. 46–48) because it is more reactive. To our delight, the PEC strategy is applicable to chlorination of alkanes including *n*-pentane and *n*-hexane (23, 24), and the chlorination mostly occurred in the tertiary C−H bond position (Supplementary Figs. 49 and 50). Because *n*-pentane and *n*-octane are highly volatile, some technical challenge remains to achieve high conversion by using the unsealed PEC reactor in our work. We are working on developing airtight reactor to overcome this challenge. Most of the products are detectable on GC, as evidenced by the comparable yield and conversion data that suggests the polymerization and other side reactions can be largely neglectable. Moreover, the halide anion used in the PEC halogenation can be extended from Cl^−^ to Br^−^, and I^−^ using NaBr and NaI as the reactants, respectively, achieving bromination and iodination reactions have been achieved (25–27; Supplementary Figs. 51–53), demonstrating the generality of the PEC halogenation strategy.

### PEC halogenation based on seawater

**Fig. 7:**
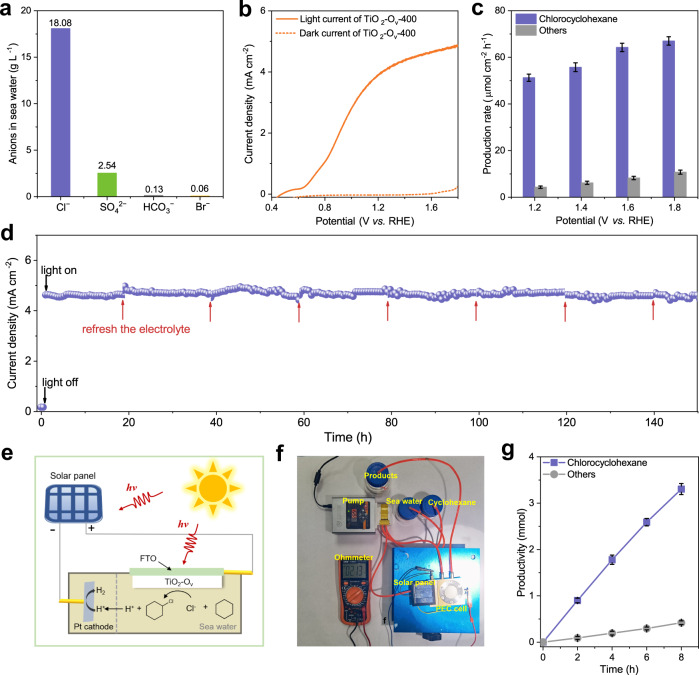
PEC chlorination based on seawater. **a** The concentration of the main anions in the natural seawater collected from the Yellow Sea, Shandong Province, China. **b** LSV curves at scan rate of 10 mV s^−1^ of TiO_2_-O_v_-400 photoanode in seawater under AM 1.5G (100 mW cm^−2^) illumination (solid line) or in the dark (dashed line). **c** Production rate of chlorocyclohexane on TiO_2_-O_v_-400 photoanode using seawater as electrolyte under AM 1.5G (100 mW cm^−2^) illumination at different potentials for 2 h in an H-type photoelectrocell. **d** Current-time (*I-t*) curve of TiO_2_-O_v_-400 for PEC cyclohexane chlorination in seawater for 150 h reaction. **e** Schematic illustration of the self-powered PEC system. **f** The photo of the self-powered PEC system under AM 1.5G (100 mW cm^−2^) illumination. **g** Productivity of the PEC chlorination products in self-powered PEC system. The error bars represent the relative deviation, which is within 5%.

For a practical application, we sought to perform the halogenating reaction coupled with H_2_ production by directly using seawater as both the electrolyte and Cl^−^ source, which provides a route to directly use renewable solar and ocean resource for recycling of halogen into valuable chemicals. Natural seawater used in this work was collected from the Yellow Sea (36.0532°N, 120.4115°E), Shandong Province, China, with chloride ion concentration of ∼18 g L^−1^ (determined by ion chromatography; Fig. 7a). The pH of the natural seawater was measured to be 7.4 after simply filtering out silts and algae microorganisms, etc. We then evaluated the PEC halogenation performance of TiO_2_-O_v_-400 in the natural seawater with 0.5 M of cyclohexane in H-type quartz cell. As shown in Fig. 7b, the TiO_2_-O_v_-400 exhibits an obviously enhanced photocurrent under AM 1.5G (100 mW cm^−2^) illumination. Specifically, the photocurrent density reaches 4.6 mA cm^−2^ at 1.6 V vs. RHE with chlorocyclohexane production rate of 64.5 μmol cm^−2^ h^−1^ and selectivity of 88.6% (Fig. 7c and Supplementary Fig. 54a). The production rate of chlorocyclohexane could be further improved with the increase of potential, but the production rate of the by-products would also increase (Fig. 7c and Supplementary Fig. 54b). The long-term stability of TiO_2_-O_v_-400 photoanode in seawater was also examined. As shown in Fig. 7d, the photocurrent density of TiO_2_-O_v_-400 maintained ~98% after 150 h at 1.6 V vs. RHE under AM 1.5G (100 mW cm^−2^) illumination in seawater. Moreover, the initial nanoarray structure and the oxygen-vacancy are stable for TiO_2_-O_v_-400 photoanode (Supplementary Fig. 55). The vacancy-related properties of TiO_2_-O_v_-400 photoanode were largely maintained, such as the light absorption range, energy band position (2.75 eV), the photo-induced charge separation efficiency and the charge transfer properties (Supplementary Fig. 56), demonstrating the high stability of TiO_2_-O_v_-400 photoanode.

In addition, we designed a self-powered PEC system, which is equipped with a solar panel (~2 V) to provide constant bias, a homemade PEC flow cell with TiO_2_-O_v_-400 photoanode, and seawater was directly utilised as the electrolyte and Cl^−^ source (Fig. 7e, f). As shown in Fig. 7g, this self-powered PEC system successfully achieved C–H halogenation of cyclohexane with chlorocyclohexane productivity of 3.3 mmol (~412 µmol h^−1^) and H_2_ productivity of ~9.2 mL h^−1^ after 8 h of reaction under AM 1.5G illumination (100 mW cm^−2^), demonstrating the potential of the PEC strategy in using seawater for sustainable organic halides production.

## Discussion

In summary, we achieved a highly efficient PEC C–H halogenation in a neutral aqueous solution to produce high value-added organic halides and promote cathodic H_2_ production by using an oxygen-vacancy-rich TiO_2_ photoanode. Experimental studies and DFT calculations reveal that the halide ions can be adsorbed at the oxygen-vacancy sites of photoanode, which promotes the oxidation of halide ions to free-radical and followed C–H halogenation process. We confirm that this PEC strategy exhibits broad substrate scope including arenes, heteroarenes, nonpolar cycloalkanes, and aliphatic hydrocarbons. Moreover, we designed a self-powered PEC system and directly utilized seawater as the electrolyte and the Cl^−^ source, which achieved chlorocyclohexane productivity of 412 µmol h^−1^ coupled with H_2_ productivity of 9.2 mL h^−1^. This work may open a promising sustainable approach for photoelectrochemical C–H halogenation coupling H_2_ production under mild conditions.

It should be noted that while the electrocatalysis (especially photovoltaics combined with electrolysis system) shows higher energy conversion efficiency in many catalytic reactions than PEC does, it is still important to develop PEC technology because it is ideally powered by sunlight^2^. PEC technology is still in its infancy and many fundamental and technical challenges remain that make it currently less competitive with electrocatalysis, such as the low energy efficiency and relatively lower catalyst activity. Nevertheless, the continuous innovation of the advanced photoelectrocatalysts and PEC devices with enhanced efficiency^31^ have shed light on PEC application in the future. We believe that the synergetic development of electrocatalysis, photocatalysis, and PEC technologies for material synthesis and energy conversion is essential to establish a sustainable society that doesn’t rely on fossil fuels.

## Methods

### General information

Except noted, all chemicals were purchased and used without further purification.

### Catalyst preparation

#### Preparation of TiO_2_ photoanode

TiO_2_ nanorod array was prepared via a facile hydrothermal method^32^. Initially, the fluorine doped conductive SnO_2_ (FTO, 7 Ω, light transmittance 80%, active area 20 × 20 × 2.2 mm) was sequentially washed with solution containing acetone, isopropanol, and deionized water (1:1:1) to remove surficial contaminants. Then, 0.175 mL of titanium butoxide was dropwise added into a H_2_O/HCl mix solution with equal volume (6 mL) of H_2_O and HCl (36.5–38 wt%) under continuous stirring. The resulting solution and the pre-treated FTO substrate were transferred and sealed in a Teflon-lined stainless-steel autoclave (25 mL) and heated to 150 °C for 20 h. The as-obtained TiO_2_ nanorod array was removed from the autoclave, washed thoroughly with distilled water and dried in air, then followed by annealing at 350 °C in an ambient environment for 2 h (heating rate: 5 °C min^−1^) to improve the crystallinity of TiO_2_ nanorods and enhance their contact to the FTO substrate.

#### Preparation of TiO_2_-O_v_ photoanode

The pristine TiO_2_ nanorod array was annealed in H_2_ atmosphere (10% H_2_ and 90% Ar) at various temperatures (200, 350, 400, and 450 °C) for 2 h (heating rate: 5° min^−1^). The H_2_-treated samples are denoted as TiO_2_-O_v_-*T* (*T* represents the temperatures, 200, 350, 400, and 450 °C).

#### Preparation of WO_3_ photoanode

WO_3_ nanosquare array was prepared via a facile hydrothermal method^42^. Typically, 6 mL of HCl (3 M) was dropwise added into Na_2_WO_4_·2H_2_O (10 mM) solution under constant stirring at room temperature. Subsequently, 0.2 g of (NH_4_)_2_C_2_O_4_ was added into the above suspension and deionized water was added to 70 mL with continual stirring for 30 min. The resulting solution and the pre-treated FTO substrate were transferred and sealed in a Teflon-lined stainless-steel autoclave (25 mL) and heated to 150 °C for 2 h. The as-obtained WO_3_ precursor simple was washed thoroughly with distilled water and calcined in air at 450 °C for 2 h (heating rate: 5 °C min^−1^) to obtain WO_3_ nanosquare array photoanode.

#### Preparation of BiVO_4_ photoanode

BiVO_4_ photoanode was synthesised following a two-step method^43^. Firstly, Bi-metal was electrodeposited onto the FTO substrate (Bi@FTO). The room temperature electrodeposition was carried out in a three-electrode configuration using an electrochemical workstation (CHI 760E, CH Instrument Co. USA), and was accomplished by holding the FTO electrode at −0.6 V (vs. Ag/AgCl) for 600 s while the FTO was in contact with an aqueous solution of 1 mmol Bi(NO_3_)_3_·5H_2_O in 50 mL of water and 100 mL ethylene glycol. The electrodeposited Bi was then washed with ethanol. The as-obtained the Bi@FTO samples were dipped in 0.2 mL VO(acac)_2_ dimethyl sulfoxide (DMSO) solution (0.2 M). The samples were then heated in air at 450 °C for 2 h (heating rate: 2 °C min^−1^). The samples were then left to cool naturally to ambient temperature. Residual V_2_O_5_ was removed by stirring the samples in 1 M NaOH. Finally, the BiVO_4_ photoanode was cleaned by deionized water and dried in air.

#### Preparation of ZnO photoanode

ZnO nanowire array were prepared by a facile hydrothermal method^44^. Briefly, 100 mL of a 0.1 M solution of Zn(CH_3_COOH)_2_ in absolute ethanol was prepared with ultrasonic agitation. A thin layer of ZnO seeds were obtained by spin-coating of the above solution on the FTO substrates, followed by annealing at 350 °C for 30 min. The seeded substrates were sealed in a reagent solution containing 0.1 M Zn(NO_3_)_2_ and 0.1 M HMT in an autoclave and then heated to 110 °C for 24 h for nanowire growth. The nanowire substrate was removed from the autoclave, washed thoroughly with distilled water and dried in air.

### Sample characterizations

X-ray powder diffraction (XRD) patterns were recorded using a Shimadzu XRD-6000 diffractometer equipped with a graphite-filtered Cu Kα radiation source (*λ* = 0.15418 nm). X-ray photoelectron spectra (XPS) were performed on a Thermo VG ESCALAB 250 X-ray photoelectron spectrometer at a pressure of about 2 × 10^−9^ Pa using Al Kα X-rays as the excitation source. Scanning electrode microscope (SEM) images were taken on a Zeiss SUPRA 55 Field Emission scanning electron microscope operated at 20 kV. Transmission electron microscopy (TEM) images were recorded with JEOL JEM-2010 high resolution (HR-)TEM operated at 200 kV, combined with energy dispersive X-ray spectroscopy (EDX). Powder samples used in this work were scraped from the TiO_2_ nanoarray-FTO glass. UV–vis diffuse reflectance spectrum was recorded in the spectral region of 300–800 nm on a Shimadzu UV-3000 spectrophotometer. Photoluminescence (PL) spectra were obtained at ambient temperature using a HitachiF-7000 fluorescence spectrophotometer with an excitation wavelength of 300 nm. The bandgap of samples was determined based on the Tauc plot. The steady-state and transient surface photovoltage (SPV) measurements were performed on a lock-in amplifier (SR830-DSP) with a light chopper (SR540) and a photovoltaic cell. A 300 W xenon lamp in conjunction with a double-prism monochromator (Zolix SBP500) provided monochromatic light as the light source. The concentration of chloride ion in solution was analyzed using the ion chromatography (IC, 2016 Timebase ICS-5000). Room-temperature EPR spectra of samples were collected on a JES-FA200 X ESR spectrometer (298 K, 9063.386 MHz). 5,5-Dimethyl-1-pyrroline N-oxide (DMPO) was used as the spin-trapping agent. 0.5 mg samples were dispersed in 2 mL toluene, then 50 µL DMPO was added into the solution, and after filled with argon irradiated with 300 W Xenon lamp (Microsolar 300; Beijing Perfectlight) with an AM 1.5G filter (100 mW cm^−2^) for ESR measurement.

### Photoelectrochemical characterization

Photoelectrochemical measurements were conducted on an electrochemical workstation (CHI 760E, CH Instruments, Inc.) using a sealed H-type glass cell in a three-electrode system (Ag/AgCl electrode as reference electrode and Pt foil as counter electrode). Nafion proton exchange membrane was used to separate the photoanode from the cathode chamber. The simulated solar illumination was obtained from a 300 W Xenon lamp with an AM 1.5 G filter (100 mW cm^−2^). The electrolyte contains 0.5 M NaCl aqueous solution. Photocurrent was recorded from 0.4 to 1.8 V vs. RHE at a scan rate of 10 mV s^−1^ with the presence of cyclohexane. All potentials mentioned in this work were converted to potentials versus RHE (in volts) according to Eq. 1:

1 $${E}_{{{{{{\rm{RHE}}}}}}}={E}_{{{{{{\rm{Ag}}}}}}/{{{{{\rm{AgCl}}}}}}}+{E}_{{{{{{\rm{Ag}}}}}}/{{{{{\rm{AgCl}}}}}}{{{{{\rm{vs}}}}}}.{{{{{\rm{NHE}}}}}}}+0.059{{{{{\rm{pH}}}}}}$$

Where *E*_Ag/AgCl vs. NHE_ in Eq. 1 is 0.197 V at 20 °C.

The photoelectrochemical impedance spectra were collected in a frequency range of 1~10^5^ Hz with an amplitude of 1 mV under AM 1.5G, 100 mW cm^−2^ illumination, from 0.4 V to 1.8 V vs. RHE. Mott–Schottky plots were measured at 1500 Hz in dark with potential varied from −0.4 to 1.0 V vs. RHE.

IPCE was measured under monochromatic irradiation under one sun illumination (AM 1.5 G, 100 mW cm^−2^) equipped with a monochromator at 1.6 V vs. RHE:

2 $${{{{{\rm{IPCE}}}}}}=\,\frac{\left[(\frac{1240}{\lambda })\times ({J}_{{{{{{\rm{light}}}}}}}-{J}_{{{{{{\rm{dark}}}}}}})\right]}{P}\times 100 \%$$

where *λ* is the wavelength, *J*_light_ is the photocurrent density under irradiation, *J*_dark_ is the current density under dark condition, and *P* is the incident light power density.

### Photoelectrocatalysis characterizations

Under general reaction conditions, the photoanode was immersed in 0.5 M NaCl (20 ml) containing cyclohexane (2 mL, 18.8 mmol) in a sealed H-type cell, and Ar was bubbled through the solution for 30 min. The solution was stirred continuously at 500 rotations min^−1^. Then, the photoelectrochemical chlorination was performed at a potential of 1.6 V vs. RHE for 2 h under AM 1.5G, 100 mW cm^−2^ illumination, and the temperature was maintained at room temperature (around 298 K). After reaction, 1 mL of solution was taken out from the cell and analyzed using gas chromatography mass spectrometry (GC-MS, Agilent Technologies, GC7890B, MS 5977B) to identify production or dissolve in 2 mL absolute ethanol for further quantitative analysis using an Agilent chromatograph equipped with a HP-5 (300 × d mm, 8 μm) column and FID detector.

Selectivity of products was calculated based on:

3 $${{{{{\rm{Selectivity}}}}}}=\,\frac{{{{{{\rm{content}}}}}}\,{{{{{\rm{of}}}}}}\,{{{{{\rm{corre}}}}}}s{{{{{\rm{ponding}}}}}}\,{{{{{\rm{product}}}}}}\,{{{{{\rm{detected}}}}}}\,{{{{{\rm{via}}}}}}\,{{{{{\rm{GC}}}}}}}{{{{{{\rm{consumption}}}}}}\,{{{{{\rm{of}}}}}}\,{{{{{\rm{reactant}}}}}}}\times 100 \%$$

4 $${{{{{\rm{Conversion}}}}}}\,{{{{{\rm{rate}}}}}}=\frac{{{{{{\rm{consumption}}}}}}\,{{{{{\rm{of}}}}}}\,{{{{{\rm{reactant}}}}}}}{t\times A}$$

where *t* is the reaction time (h), and *A* is the area of photoanode (cm^−2^).

Faradaic efficiency was calculated by:

5 $${{{{{\rm{Faradaic}}}}}}\,{{{{{\rm{efficiency}}}}}}=\frac{{e}_{{{{{{\rm{products}}}}}}}\times {n}_{{{{{{\rm{products}}}}}}}\times N}{{{{{{\rm{Q}}}}}}/n}\times 100 \%$$

where *e*_products_ is the number of holes required to oxidize cyclohexane/H_2_O molecule to products, including chlorocyclohexane (*e* = 2), cyclohexanol (*e* = 2), cyclohexene (*e* = 2), bicyclohexyl (*e* = 2), cyclohexanone (*e* = 4), O_2_ (*e* = 4). *n*_products_ is the productivity of products, *N* is Avogadro’s constant (*N* = 6.02 × 10^23^), Q is the quantity of electric charge, and *n* is the elementary charge (*e* = 1.602 × 10^−19^ C).

The external quantum efficiency of photon-to-products was calculated based on IPCE and faradaic efficiency of products. External quantum efficiency of DHA was calculated by:

6 $${{{{{\rm{QE}}}}}} 	=\; \frac{{{{{{\rm{Number}}}}}}\,{{{{{\rm{of}}}}}}\,{{{{{\rm{holes}}}}}}\,{{{{{\rm{to}}}}}}\,{{{{{\rm{chloridize}}}}}}\,{{{{{\rm{cyclohexane}}}}}}\,}{{{{{{\rm{Number}}}}}}\,{{{{{\rm{of}}}}}}\,{{{{{\rm{incident}}}}}}\,{{{{{\rm{photons}}}}}}}\times 100 \% \\ 	=\; {{{{{\rm{IPCE}}}}}}\times {{{{{\rm{Faradaic}}}}}}\,{{{{{\rm{efficiency}}}}}}\times 100 \%$$

Oxygen production was measured using a Neo-FOX oxygen-sensing system (Ocean Optics Inc.) equipped with a FOX Y probe inserted into the headspace of an airtight cell. The cell was purged with nitrogen for 2 h prior to measurement. The oxygen fluorescence signal was allowed to stabilize after the nitrogen flow was stopped. Once the residual oxygen signal was constant, bias was applied to the electrode. The oxygen evolution was monitored at 1.6 V vs. RHE.

The space-time yield of H_2_ was obtained by gas-collecting method of drainage water. For long-term photoelectrochemical measurement by constant potential (CP) strategies, the productivity of H_2_ was calculated based on the charge transfer of cyclohexane chlorination.

### Computational details

#### Model construction

Two models named as TiO_2_-O_v_ and TiO_2_ were constructed to represent the photoanode TiO_2_-O_V_-400 used in this work and the control sample TiO_2_ without oxygen vacancy, respectively. Firstly, the model of bulk rutile TiO_2_ was constructed according to the single crystal X-ray diffraction measurement in previous literature^45^. The space group of rutile TiO_2_ is P42/mnm, with the lattice parameters of *a* = *b* = 4.59 Å, *c* = 2.96 Å, *α* = *β* = *γ* = 90°. According to the HAADF-STEM image of TiO_2_-O_v_-400 (Fig. 2c) that displays the interplanar spacings of (110) and (101) facets, the exposed facet of TiO_2_-O_v_-400 is deduced to be the (011) facet, which is perpendicular to the (110) and (101) facets. Since rutile TiO_2_ is isotropic in the lattice *a* and *b* directions, the (011) facet is identical to the (101) facet. Moreover, the (101) facet has been widely reported to be one of the preferably exposed facets of rutile TiO_2_^38^. Thus the (101) facet of rutile TiO_2_ was cleaved, containing four layers of Ti atoms, eight layers of O atoms, and a vacuum layer of 20 Å. According to the previous literature, the surface of TiO_2_ may be passivated by OH* or H* (* represents the surface of rutile TiO_2_) in aqueous environment^46–49^, therefore, under the reaction condition here (1.6 V vs. RHE), the surface of TiO_2_-O_V_ may be covered with ‒H, –OH, or –O, or terminated with Ti. Thus, four surface models terminated with Ti, –H, –OH, and –O were constructed and named as S1, S2, S3, and S4, respectively (Supplementary Note 2). The optimized geometries of models S1, S2, S3, and S4 are displayed in Supplementary Fig. S24. Then the Gibbs free energies of these four facets were calculated referred to the method proposed by Valdes *et al*^50^ and displayed in Supplementary Fig. S25. It was found that the surface S3 where the coordination unsaturated Ti atoms are covered by the OH* are the most stable with the lowest Gibbs free energy among S1, S2, S3, and S4 under reaction condition, 1.6 V vs. RHE (Supplementary Fig. S25). Therefore, the facet S3 with the chemical formula of Ti_32_O_77_H_8_ is used as model TiO_2_-O_V_ in the following calculations. The chemical formula of model TiO_2_ is Ti_32_O_80_H_8_. The amount of oxygen vacancy on model TiO_2_-O_v_ is 37.5% on the surface, which was referred to the X-ray photoelectron spectra result that the amount of oxygen vacancy is 35% on the surface of TiO_2_-O_v_-400 (Supplementary Table 2). When calculating the mechanism of the generation of Cl* and Cl_2_* on TiO_2_-O_v_, the intermediate models of Cl^–^* (one Na^+^ cation was added to keep the model neutral), Cl*, and Cl_2_* were constructed. For each intermediate model, the possible adsorption sites of top, bridge, and fcc sites were screened to find the optimal adsorption site.

#### Computational methods

Spin-polarized DFT calculations were performed with the plane wave implementation in Cambridge Sequential Total Energy Package (CASTEP)^51^. The Perdew-Burke-Ernzerhof (PBE) in generalized gradient approximation (GGA) was used as the exchange-correlation functional^52^. The ionic core of Ti was described with the ultrasoft pseudopotentials to improve transferability and reduce the number of plane waves needed to expand the Kohn-Sham orbitals. The cutoff energy was set as 400 eV and the *k*-point meshes was set as 3 × 3 × 1^53^. To validate the convergence of the basis set size, the Δ*G* for the desorption of Cl* was calculated with a 600 eV cut-off. The Δ*G* varied by less than 0.015 eV, supporting the choice of 400 eV cutoff in this work. To validate the convergence of *k*-point meshes, the Δ*G* for the desorption of Cl* was calculated with a 5 × 5 × 1 *k*-point meshes. The Δ*G* varied by less than 0.010 eV, indicating that the 3 × 3 × 1 *k*-point meshes was converged. The Broyden-Fletcher-Goldfarb-Shanno (BFGS) algorithm was used to search the potential energy surface during geometry optimization^54^. The following three points were used during geometry optimization: (1) a maximum energy tolerance of 1.0 × 10^–6^ eV per atom, (2) a maximum force tolerance of 3.0 × 10^–2^ eV/Å, and (3) a maximum displacement tolerance of 1.0 × 10^–3^ Å.

#### Proposed mechanism

It is proposed that the chlorination of cyclohexane mainly follows a free-radical reaction mechanism^20,36^. Since chlorocyclohexane is the main product rather than C–C coupling products for photoelectrochemical chlorination of cyclohexane in this work, the chlorination of cyclohexane probably follows the free-radical chain reaction mechanism. The reaction formula of the chlorination of cyclohexane in this work is deduced to be as follows:

A $$\ast +{{{{{{\rm{Cl}}}}}}}^{-}\to {{{{{{\rm{Cl}}}}}}}^{-}\ast {\,}(\ast {{{{{\rm{represents}}}}}}\,{{{{{\rm{the}}}}}}\,{{{{{\rm{reaction}}}}}}\,{{{{{\rm{site}}}}}})$$

B $${{{{{{\rm{Cl}}}}}}}^{-}\ast \to {{{{{{\rm{Cl}}}}}}}\ast +{{{{{{\rm{e}}}}}}}^{{-}}$$

C $${{{{{{\rm{Cl}}}}}}}^{\ast }{\,}{\to }{\,}^{\ast }+{{{{{\rm{Cl}}}}}}{\cdot}$$

D $$\ast +{{{{{\rm{Cl}}}}}}^{-}\to {{{{{{\rm{Cl}}}}}}}^{{-}\ast}$$

E $${{{{{{\rm{Cl}}}}}}}^{{-}\ast }\to {{{{{{\rm{Cl}}}}}}}\ast +{{{{{{\rm{e}}}}}}}^{{-}}$$

F $${{{{{{\rm{Cl}}}}}}}^{\ast }+{{{{{\rm{Cl}}}}}}^{-}\to {{{{{{{\rm{Cl}}}}}}}_{2}}^{\ast }+{{{{{{\rm{e}}}}}}}^{{-}}$$

G $${{{{{\rm{Cl}}}}}}{\hskip -2.2pt}\cdot +{\,}{{{{{\rm{Cl}}}}}}\cdot \to {{{{{{\rm{Cl}}}}}}}_{2}({{{{{\rm{in}}}}}}\,{{{{{\rm{solvent}}}}}})$$

H $${{{{{{{\rm{Cl}}}}}}}_{2}}^{\ast }{\,}{\to }{\,}^{\ast }+{{{{{{\rm{Cl}}}}}}}_{2}$$

I $${{{{{\rm{Cl}}}}}}{\!}\cdot +{\,}{{{{{{\rm{C}}}}}}}_{6}{{{{{{\rm{H}}}}}}}_{12}\to {{{{{\rm{C}}}}}}_{6}{{{{{{\rm{H}}}}}}}_{11}{\!\!}\cdot +{\,}{{{{{\rm{HCl}}}}}}$$

J $${{{{{{\rm{Cl}}}}}}}_{2}+{{{{{{\rm{C}}}}}}}_{6}{{{{{{\rm{H}}}}}}}_{11}{\!}\cdot \to {{{{{{\rm{C}}}}}}}_{6}{{{{{{\rm{H}}}}}}}_{11}{{{{{\rm{Cl}}}}}}+{{{{{\rm{HCl}}}}}}$$

The Δ*G* of each elementary step was obtained by minus the Gibbs free energy of reactant with that of product. The Gibbs free energy of (Na^+^ + *e*^–^) was treated with the standard electrode potential of Na^+^/Na (–2.714 V vs. RHE)^55^ referred to the computational standard hydrogen electrode approximation^51^. The driving force of photo-induced hole on TiO_2_-O_v_ is determined to be 2.6 eV according to its band edge placement of valence band maximum (2.6 V vs. NHE, Supplementary Fig. 7d). The influence of bias was treated with the computational standard electrode potential approximation^C6^ with the term “–*eU*”. The Gibbs free energy of reactant, intermediates, and product were obtained by calculating their phonon density of states, as depicted in Eq. 1:

1 $${G}={E}+{ZPE}+{kT}\int {F}({{{{{\rm{\omega }}}}}}){{{{{\mathrm{ln}}}}}}\,\left[1-\exp \left(-\frac{\hslash {{{{{\rm{\omega }}}}}}}{kT}\right)\right]{d}{{{{{\rm{\omega }}}}}}$$

where *E* is the energy, *ZPE* is the zero-point energy, the term $kT\int F(\omega ){{{{\mathrm{ln}}}}}[1-\exp (-\frac{\hslash \omega }{kT})]d\omega$ was the correction of Gibbs free energy via thermodynamic analysis.

## Supplementary information

Supplementary Information

## Data Availability

Source data from the figures in the main manuscript are provided with this paper. Additional data that support the findings of this study are available from the corresponding author upon reasonable request. Source data are provided with this paper.

## Source data

Source data
