# Publisher Correction: Photoelectrocatalytic C–H halogenation over an oxygen vacancy-rich TiO_2_ photoanode

**DOI:** 10.1038/s41467-021-27582-0

**Published:** 2021-12-16

**Authors:** Zhenhua Li, Lan Luo, Min Li, Wangsong Chen, Yuguang Liu, Jiangrong Yang, Si-Min Xu, Hua Zhou, Lina Ma, Ming Xu, Xianggui Kong, Haohong Duan

**Affiliations:** 1 State Key Laboratory of Chemical Resource Engineering, Beijing University of Chemical Technology, Beijing, 100029 China; 2 Department of Chemistry, Tsinghua University, 30 Shuangqing Rd, Beijing, 100084 China

**Keywords:** Nanoscale materials, Photocatalysis, Photocatalysis

Correction to: *Nature Communications* 10.1038/s41467-021-26997-z, published online 18 November 2021.

The original version of this Article contained an error in Fig. 2, in which two curved arrows were inadvertently omitted. The correct version of Fig. 2 is:

**Figure 1:**
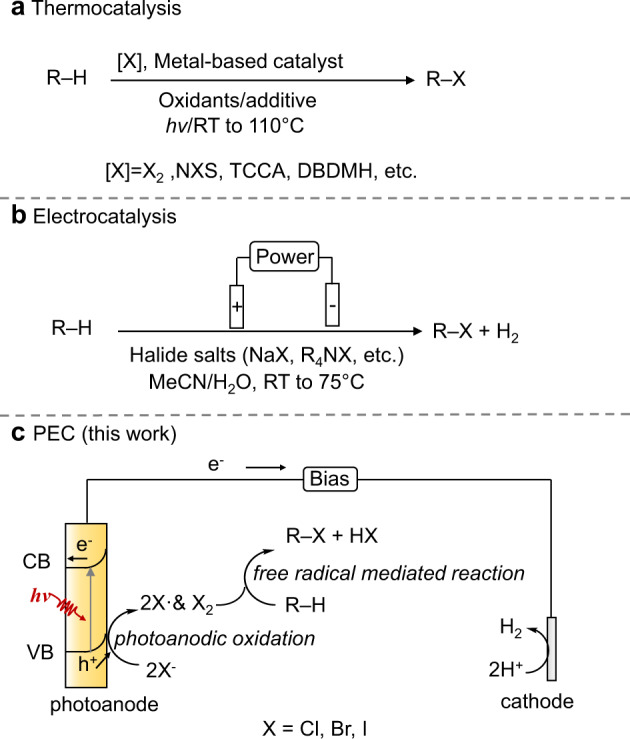
Fig. 2

which replaces the previous incorrect version:

**Figure 2:**
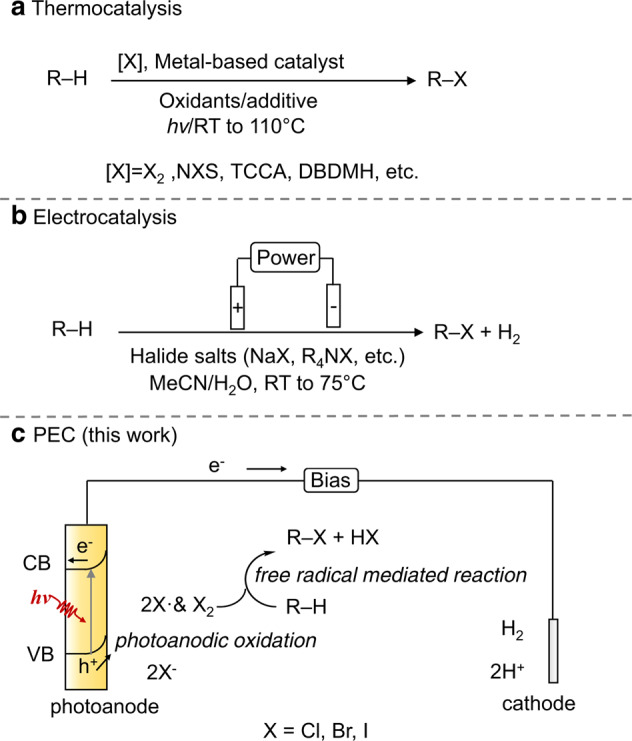
Fig. 2

This has been corrected in both the PDF and HTML versions of the Article.

The original version of this Article contained errors in Equations (B), (C), (E), (G), (H) and (I). In Equation (B), the l of Cl was superscripted and a hyphen was not superscripted, and incorrectly read: ${{{{{{\rm{Cl}}}}}}}^{-}* \to {{{{{{\rm{C}}}}}}}^{{{{{{\rm{l}}}}}}}* +{e}{-}$. The correct form of Equation (B) is: ${{{{{{\rm{Cl}}}}}}}^{{-}{\ast}} \to {{{{{\rm{Cl}}}}}}^{*} +{{\rm {e}}}^{-}$.

In Equation (C), a dot was omitted, and incorrectly read: ${{{{{{\rm{Cl}}}}}}}^{* }{\to }^{* }+{{{{{\rm{Cl}}}}}}$ The correct form of Equation (C) is: ${{{{{{\rm{Cl}}}}}}}^{* }{\to }^{* }+{{{{{\rm{Cl}}}}}}$.

In Equation (E), the l of Cl was superscripted, and incorrectly read: ${{{{{{\rm{Cl}}}}}}}^{{*} }\to {{{{{{\rm{C}}}}}}}^{{{{{{\rm{l}}}}}}* }+{{{{{{\rm{e}}}}}}}^{\mbox{-}}$. The correct form of Equation (E) is: ${{{{{{\rm{Cl}}}}}}}^{{*} }\to {{{{{\rm{Cl}}}}}}^{*} +{{{{{{\rm{e}}}}}}}^{\mbox{-}}$.

In Equation (G), a space was added, and incorrectly read: ${{{{{\rm{Cl}}}}}}\cdot +{{{{{\rm{Cl}}}}}}\cdot \to {{{{{{\rm{Cl}}}}}}}_{2}({{{{{\rm{in}}}}}}\,{{{{{\rm{solvent}}}}}})$. The correct form of Equation (G) is: ${{{{{\rm{Cl}}}}}}\cdot +\,{{{{{\rm{Cl}}}}}}\cdot \to {{{{{{\rm{Cl}}}}}}}_{2}\,({{{{{\rm{in}}}}}}\,{{{{{\rm{solvent}}}}}})$.

In Equation (H), a 2 was not subscripted, and incorrectly read: ${{{{{\rm{Cl}}}}}}{2}^{* }{\to }^{* }+{{{{{{\rm{Cl}}}}}}}_{2}$. The correct form of Equation (H) is: ${{{{{{{\rm{Cl}}}}}}}_{2}}^{* }{\to }^{* }+{{{{{{\rm{Cl}}}}}}}_{2}$.

In Equation (I), a space was added and a 6 was not subscripted, and incorrectly read: ${{{{{\rm{Cl}}}}}}\cdot +{{{{{{\rm{C}}}}}}}_{6}{{{{{{\rm{H}}}}}}}_{12}\to {{{{{\rm{C}}}}}}6{{{{{{\rm{H}}}}}}}_{11}\cdot +{{{{{\rm{HCl}}}}}}$. The correct form of Equation (I) is: ${{{{{\rm{Cl}}}}}}\cdot +{{{{{{\rm{C}}}}}}}_{6}{{{{{{\rm{H}}}}}}}_{12}\to {{{{{{\rm{C}}}}}}}_{6}{{{{{{\rm{H}}}}}}}_{11}\cdot +{{{{{\rm{HCl}}}}}}$.

This has been corrected in the PDF and HTML versions of the Article.

