# Supplementary material for: Photoelectrocatalytic C–H halogenation over an oxygen vacancy-rich TiO2 photoanode
Source: Nat Commun. 2021 Nov 18;12:6698. doi: 10.1038/s41467-021-26997-z (PMC8602285; doi:10.1038/s41467-021-26997-z)
Supplement: Supplementary file 1 — Supplementary Information [file 41467_2021_26997_MOESM1_ESM.pdf]

Supplementary Information

Photoelectrocatalytic C–H halogenation over an oxygen vacancy-rich TiO_2 photoanode

Zhenhua Li‡[1], Lan Luo‡[1], Min Li‡[2], Wangsong Chen[1], Yuguang Liu[1], Jiangrong Yang[1], Si-Min Xu[1], Hua Zhou[2], Lina Ma[1], Ming Xu[1], Xianggui Kong[1], Haohong Duan*[2]

[1]State Key Laboratory of Chemical Resource Engineering, Beijing University of Chemical Technology, Beijing 100029, China

[2]Department of Chemistry, Tsinghua University, 30 Shuangqing Rd, Haidian Qu, Beijing 100084, China

‡Zhenhua Li, Lan Luo and Min Li contributed equally to this work.

Correspondence and requests for materials should be addressed to H.D. (email: hhduan@mail.tsinghua.edu.cn).

Supplementary figures

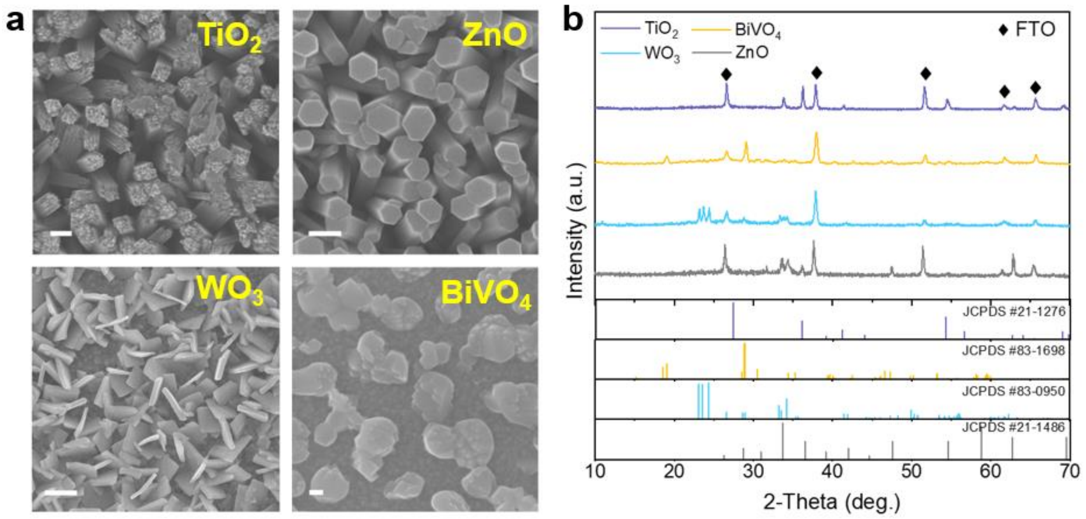

Supplementary Figure 1 | Characterisations of different photoanodes. a, Top-view SEM images of TiO_2, ZnO, WO_3 and $BiVO_4$ samples. Scale bar, 200 nm. **b**, X-ray diffraction patterns of TiO_2, $BiVO_4$, WO_3 and ZnO on FTO substrate. a.u.: arbitrary units.

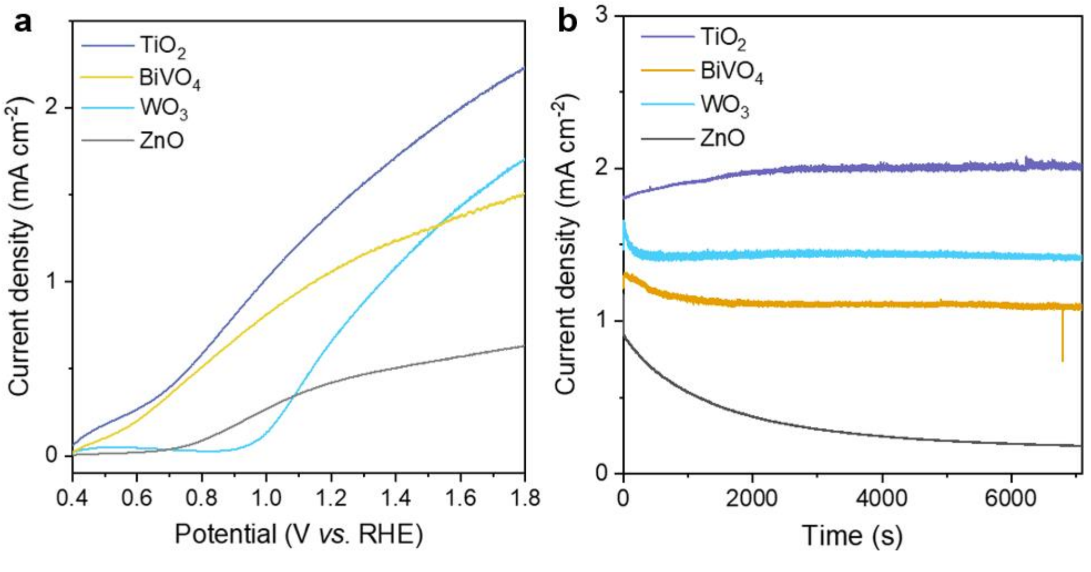

Supplementary Figure 2 | Photoelectrochemical performances. a, LSV curves of TiO_2, WO_3, $BiVO_4$ and ZnO photoanodes in 0.5 M NaCl with 18.8 mmol cyclohexane in a H-type cell under AM 1.5G, 100 mW cm^{-2} illumination. **b**, Chronoamperometric (CA) measurement of different photoanodes at 1.6 V vs. RHE in 0.5 M NaCl with 2 mL cyclohexane at room temperature.

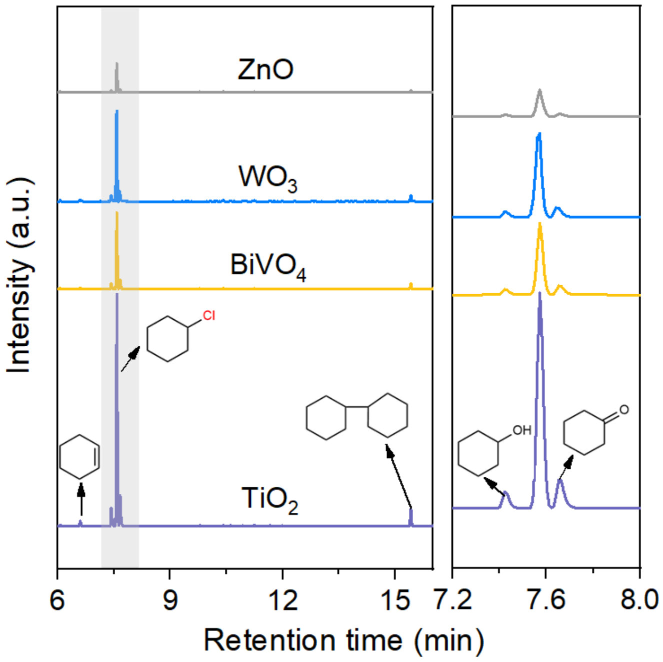

Supplementary Figure 3 | PEC cyclohexane chlorination products. GC spectra of the products for PEC cyclohexane chlorination on TiO_2, WO_3, $BiVO_4$ and ZnO photoanodes in 0.5 M NaCl electrolyte with 18.8 mmol cyclohexane at 1.6 V vs. RHE under AM 1.5G irradiation (100 mW cm^{-2}) for 2 h. a.u.: arbitrary units.

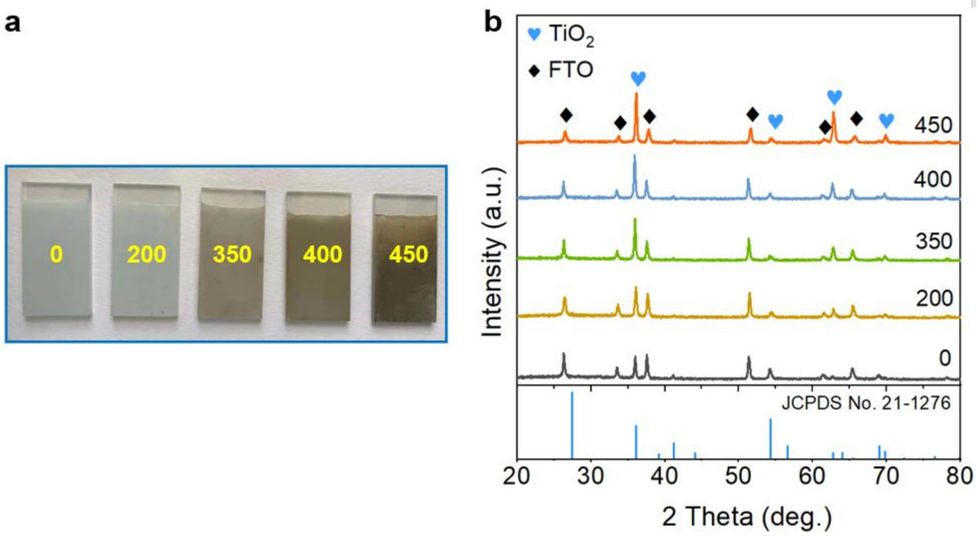

Supplementary Figure 4 | Characterization of TiO_2-O_v-*T* photoanodes. a, Photographs of TiO_2-O_v-*T* photoanodes. **b**, X-ray diffraction patterns of TiO_2, TiO_2-O_v-200, TiO_2-O_v-350, TiO_2-O_v-400 and TiO_2-O_v-450 on FTO substrate. a.u.: arbitrary units.

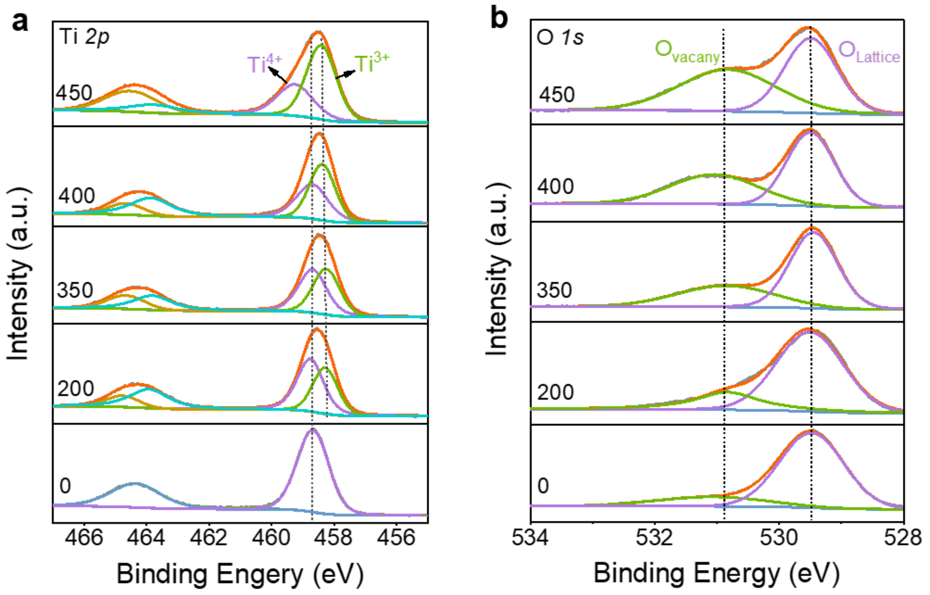

Supplementary Figure 5 | XPS spectra of TiO_2-O_v-T photoanodes. **a**, Ti *2p* and **b**, O *1s* spectra of TiO_2, TiO_2-O_v-200, TiO_2-O_v-350, TiO_2-O_v-400 and TiO_2-O_v-450 photoanodes. a.u.: arbitrary units.

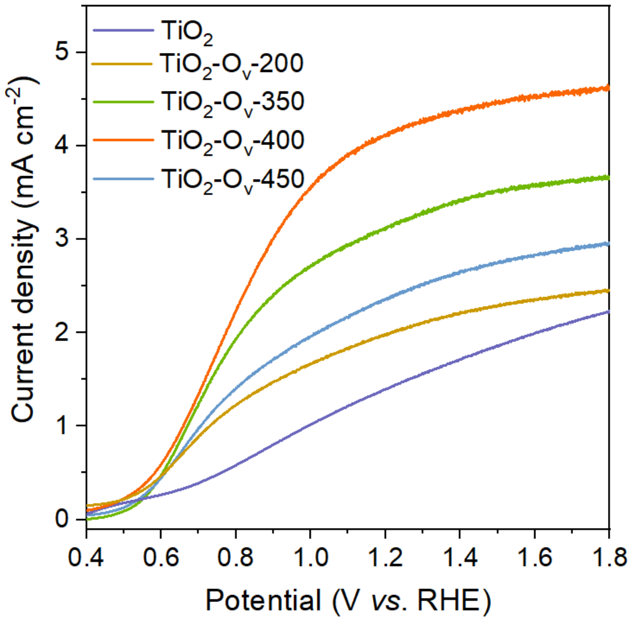

Supplementary Figure 6 | PEC performances of TiO_2-O_v-*T* photoanodes. LSV curves of TiO_2-O_v-*T* photoanodes in 0.5 M NaCl with 18.8 mmol cyclohexane in a H-type cell under AM 1.5G, 100 mW cm^{-2} illumination.

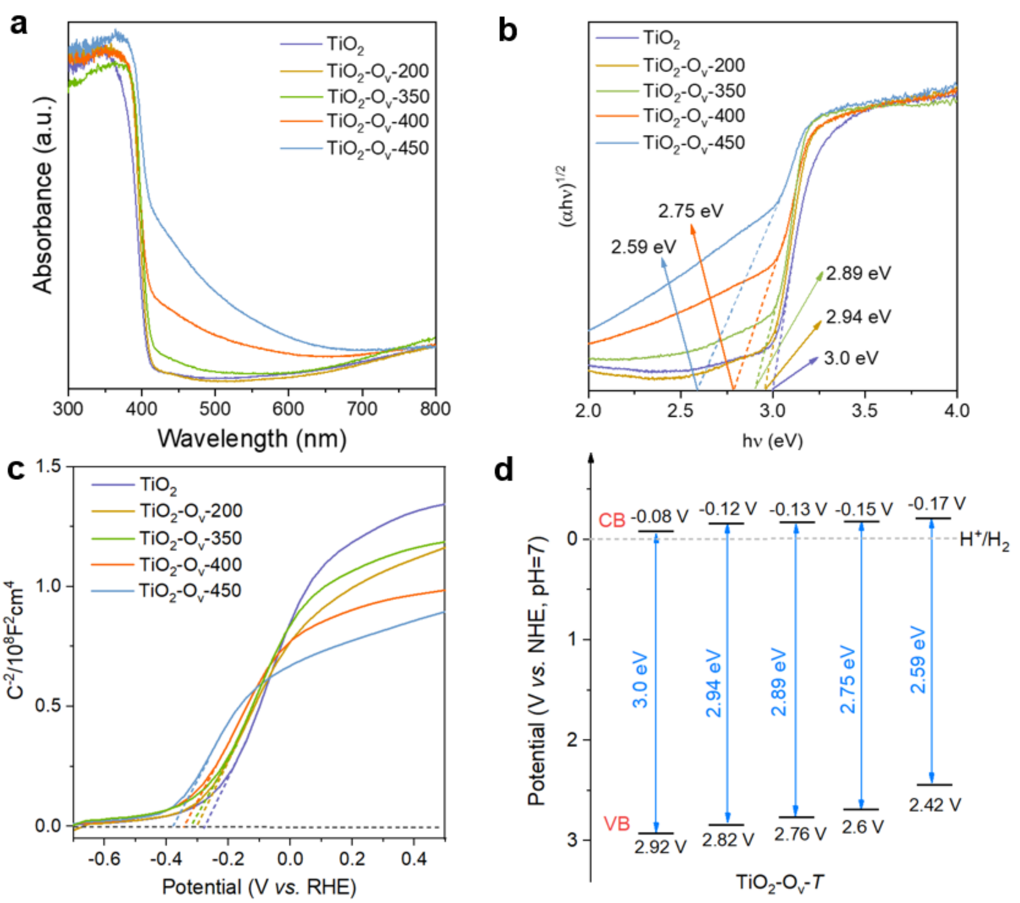

Supplementary Figure 7 | Ultraviolet–visible diffuse reflectance spectrum and bandgap of TiO_2-O_v-*T* photoanodes. **a**, Diffuse reflectance ultraviolet-visible spectra and the corresponding **b**, $(\alpha h\nu)^{1/2}$ versus photon energy plot of TiO_2-O_v-*T* samples. **c**, Mott–Schottky plots of TiO_2-O_v-*T* samples measured in 0.5 M NaCl under dark (1000 Hz). **d**, Schematic band diagrams of TiO_2-O_v-*T* samples. a.u.: arbitrary units.

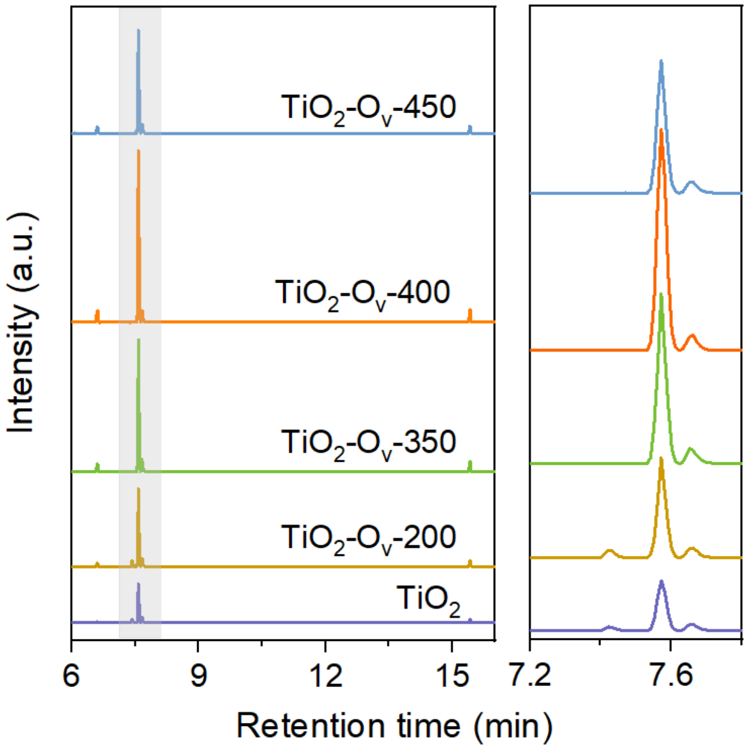

Supplementary Figure 8 | PEC chlorination of cyclohexane on TiO_2-O_v-*T* photoanodes. GC spectra of the products of PEC cyclohexane chlorination on TiO_2-O_v-*T* photoanodes in 0.5 M NaCl electrolyte with 18.8 mmol cyclohexane at 1.6 V vs. RHE under AM 1.5G, 100 mW cm^{-2} illumination for 2 h. a.u.: arbitrary units.

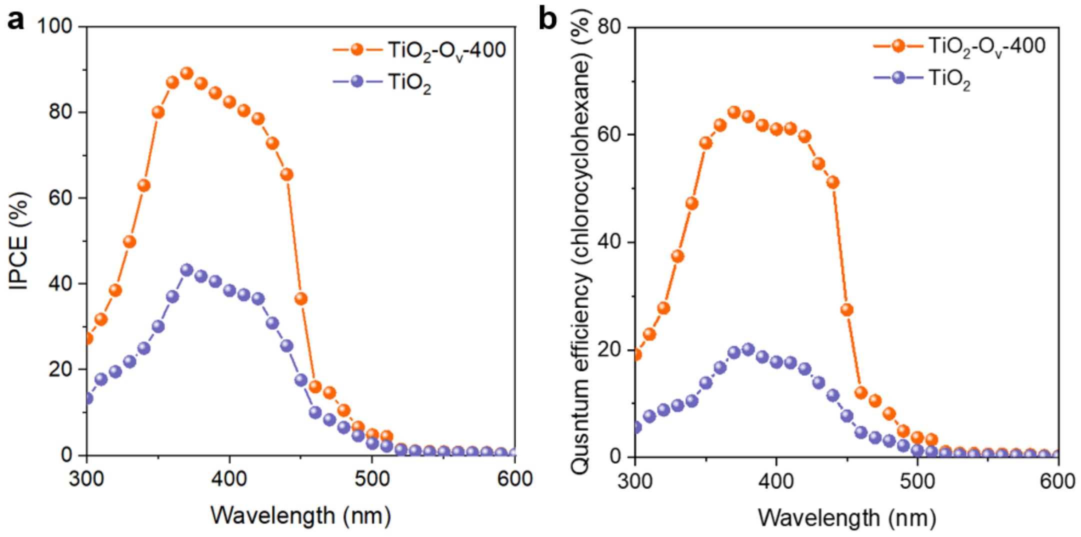

Supplementary Figure 9 | Charge carrier dynamics. a, Incident photon-to-current conversion efficiency spectra of TiO_2 and TiO_2-O_v-400 photoanodes acquired at 1.6 V vs. RHE. **b**, Incident photon-to-chlorocyclohexane conversion efficiency spectrum of TiO_2 and TiO_2-O_v-400 photoanodes acquired at 1.6 V vs. RHE.

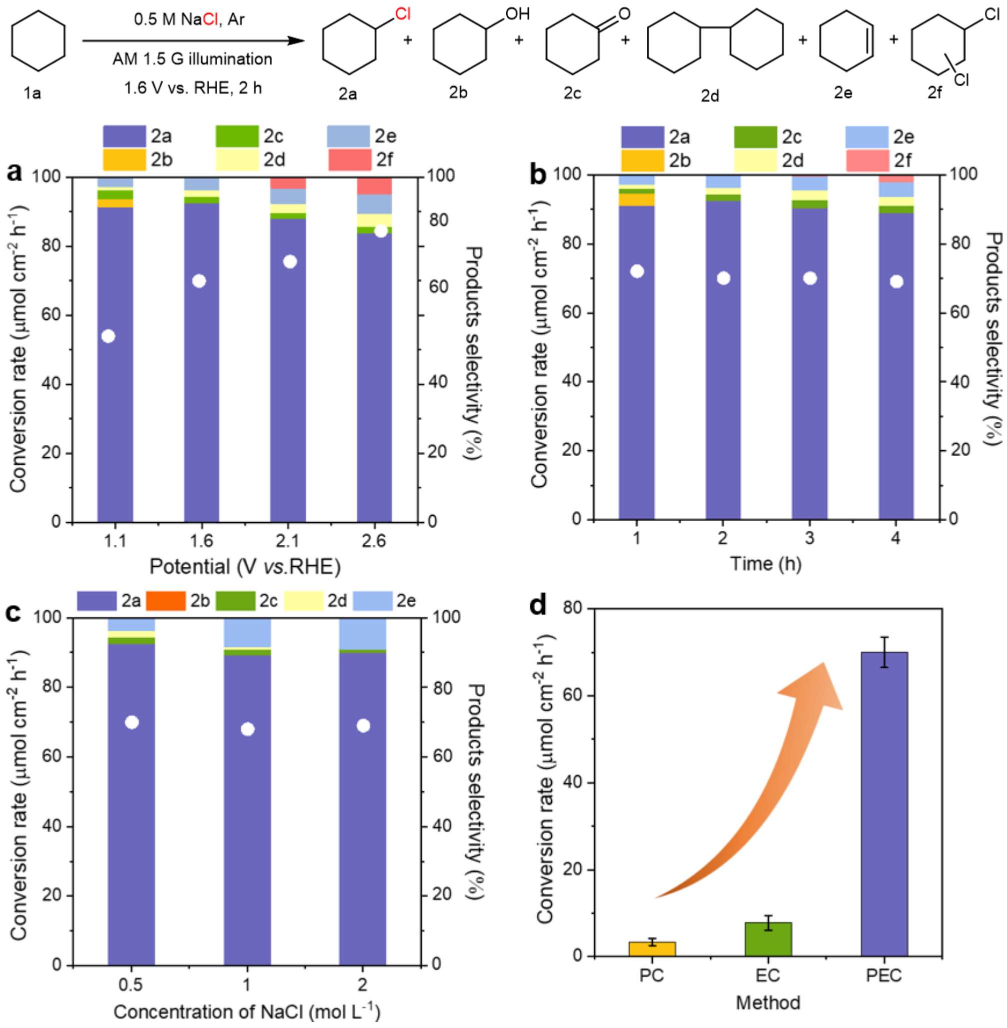

Supplementary Figure 10 | Optimisation of PEC chlorination reaction conditions. **a**, PEC conversion rate of cyclohexane and selectivity of products at different potentials in 0.5 M NaCl with 18.8 mmol cyclohexane under AM 1.5G, 100 mW cm^{-2} illumination for 2 h. **b**, PEC conversion rate of cyclohexane and selectivity of products in 0.5 M NaCl under AM 1.5G, 100 mW cm^{-2} illumination after different reaction time. **c**, PEC conversion rate of cyclohexane and selectivity of products for 2 h in different concentration of NaCl electrolyte under AM 1.5G, 100 mW cm^{-2} illumination. **d**, Different methods for the chlorination of cyclohexane. Photocatalysis (PC): photocatalysts were scraped from TiO_2-O_v-400 FTO substrate and reacted in 0.5 M NaCl under AM 1.5G, 100 mW cm^{-2} illumination for 2 h; Electrocatalysis (EC): chlorination of cyclohexane at 1.6 V vs. RHE in 0.5 M NaCl under dark for 2 h.

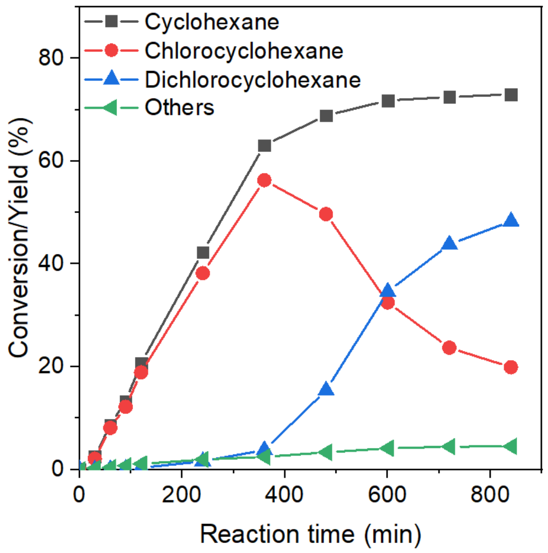

Supplementary Figure 11 | Photoelectrochemical chlorination of cyclohexane. Kinetic curves for cyclohexane transformation as a function of reaction time, other liquid products containing cyclohexanol, cyclohexanone, cyclohexene and bicyclohexane. Reaction conditions: 3 mmol cyclohexane, 20 mL 0.5 M NaCl, TiO_2-O_v-400 photoanode, 1.6 V vs. RHE under AM 1.5G, 100 mW cm^{-2} illumination.

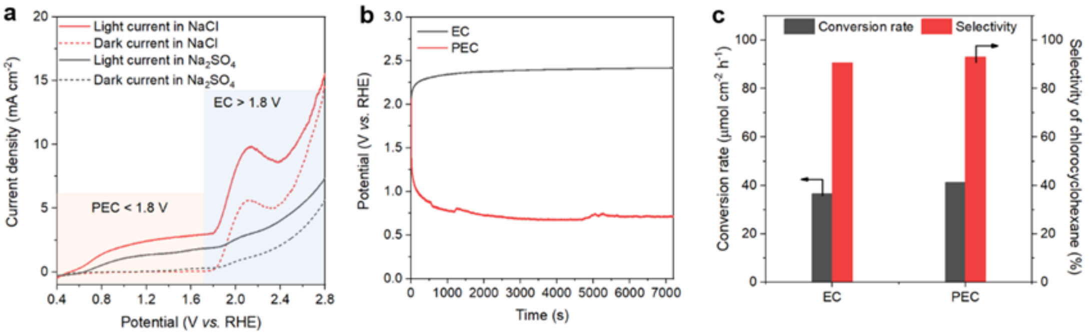

Supplementary Figure 12 | Cyclohexane chlorination in EC and PEC system. a, LSV curves of TiO_2-O_v-400 photoanode measured with 0.5 M NaCl/Na_2SO_4 in the dark or under AM 1.5G, 100 mW cm^{-2} illumination. **b**, Potential-time (*V-t*) curve of TiO_2-O_v-400 for EC/PEC cyclohexane chlorination for 2 h. **c**, Cyclohexane conversion rate and chlorocyclohexane selectivity in EC/PEC cyclohexane chlorination system. The EC experiment was carried out in 0.5 M NaCl containing cyclohexane with TiO_2-O_v-400 as the working electrode at a constant current of 2 mA cm^{-2} for 2 h. The PEC experiment was performed in 0.5 M NaCl containing 18.8 mmol cyclohexane with TiO_2-O_v-400 as the photoanode under AM 1.5G, 100 mW cm^{-2} illumination at a constant current of 2 mA cm^{-2} for 2 h.

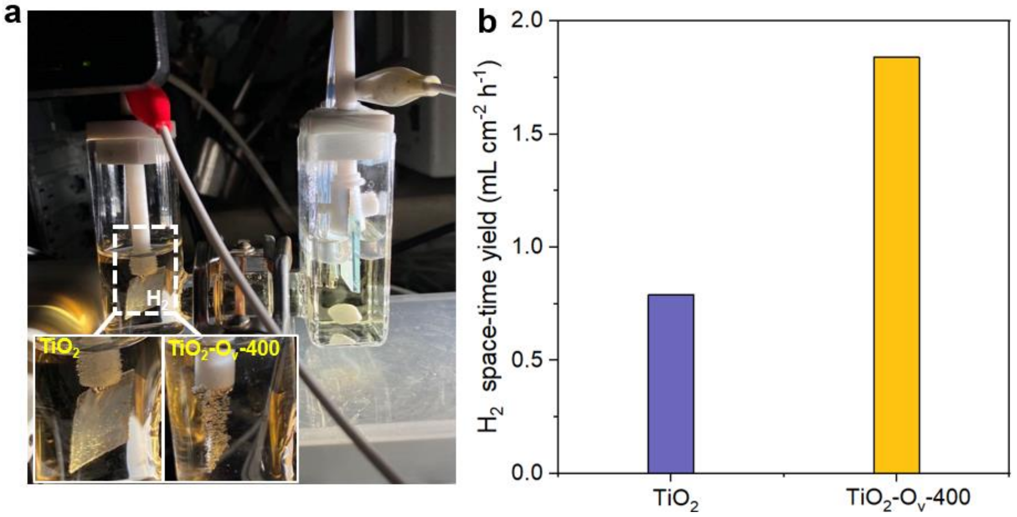

Supplementary Figure 13 | PEC chlorination of cyclohexane coupled with H_2 production. **a**, Photographs of the H-type cell for PEC cyclohexane chlorination over TiO_2-O_V-400 or TiO_2 photoanodes coupled with H_2 production at the counterpart Pt cathode. **b**, Space-time yields of H_2 by using TiO_2 and TiO_2-O_V-400 as photoanodes in 0.5 M NaCl with 18.8 mmol cyclohexane at 1.6 V vs. RHE under AM 1.5G, 100 mWcm^{-2} illumination for 2 h.

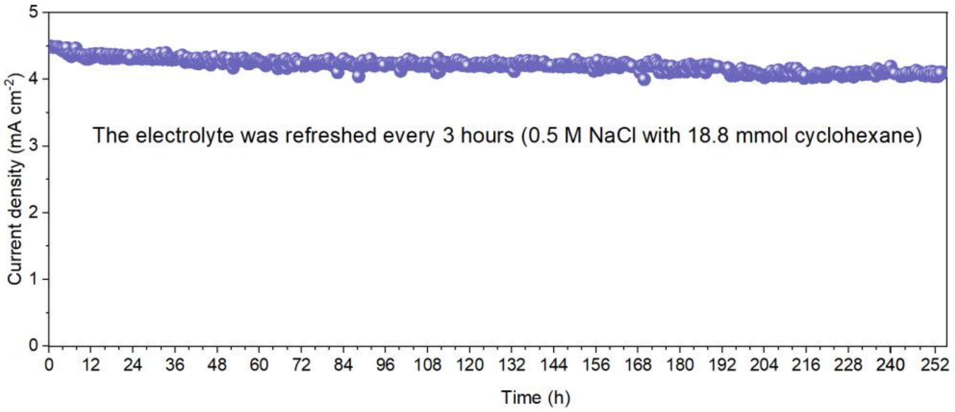

Supplementary Figure 14 | Batch stability test. Current-time (*I-t*) curve of TiO_2-O_V-400 for PEC cyclohexane chlorination for 255 h in batch reaction (85 batches). The reaction was carried out in batch reactions in 0.5 M NaCl with 18.8 mmol cyclohexane at 1.6 V vs. RHE under AM 1.5G, 100 mW cm^{-2} illumination, with 3 h for one batch. The spent TiO_2-O_V-400 photoanode was washed by ethanol and deionized water and dried under vacuum before it was ready for the next batch reaction.

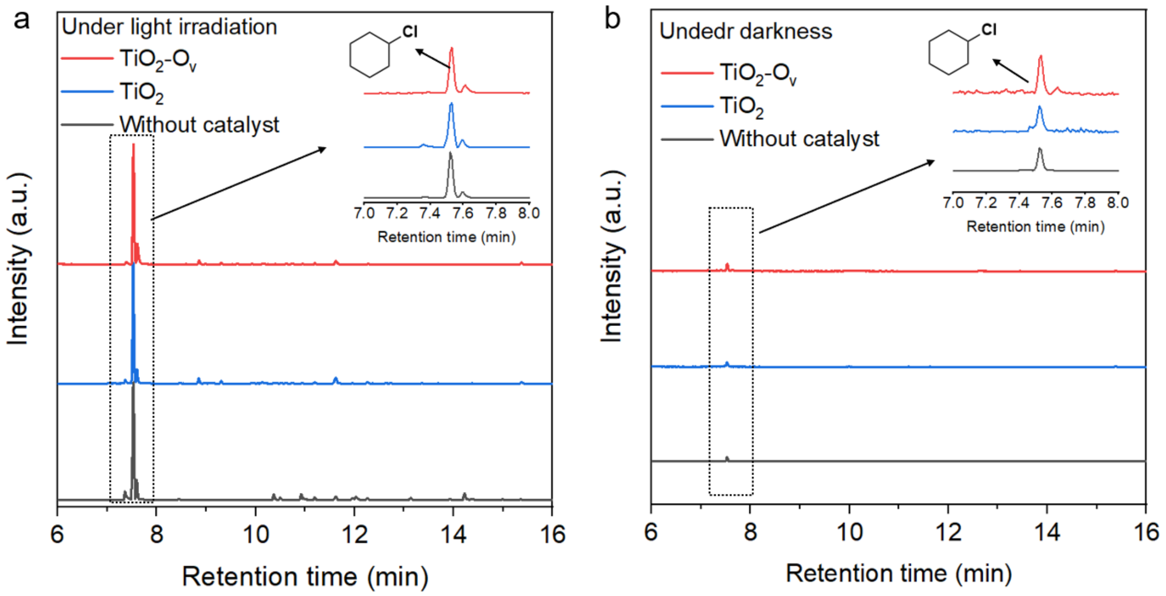

Supplementary Figure 15 | Chlorination of cyclohexane with Cl_2. a, GC spectra of the products of chlorination of cyclohexane (5 mmol) in aqueous solution used Cl_2 (1 mL) as the chlorine source under AM 1.5G, 100 mW cm^{-2} illumination and **b**, under darkness for 15 min.

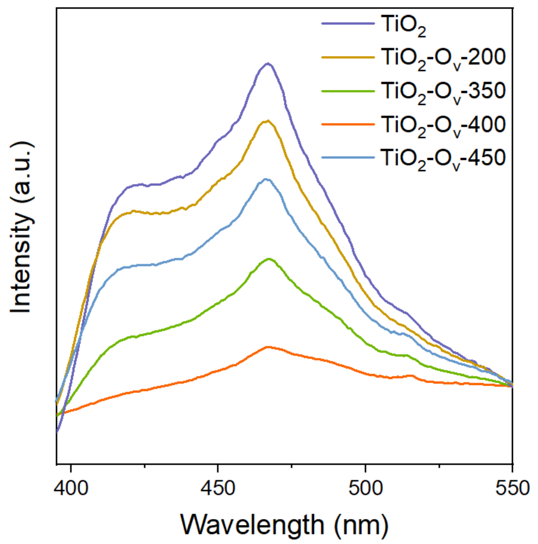

Supplementary Figure 16 | Photogenerated carrier separation efficiency. Photoluminescence spectra of TiO_2-O_V-T photoanodes, excitation wavelength 300 nm.

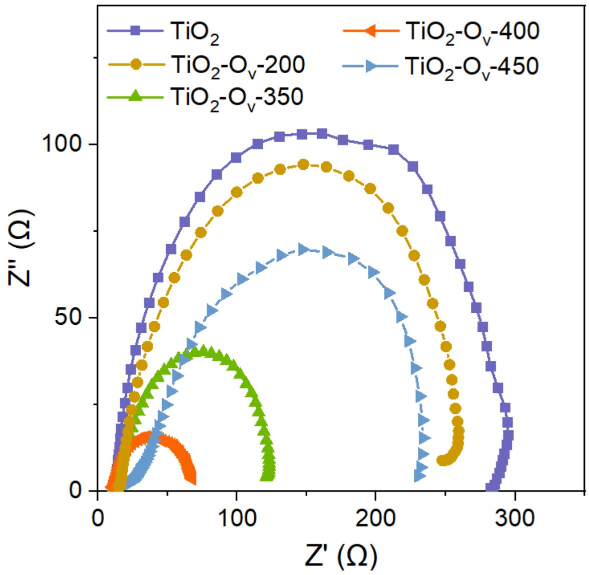

Supplementary Figure 17 | Charge transfer processes. The Nyquist plots of the EIS data measured under AM 1.5G, 100 mW cm^{-2} illumination.

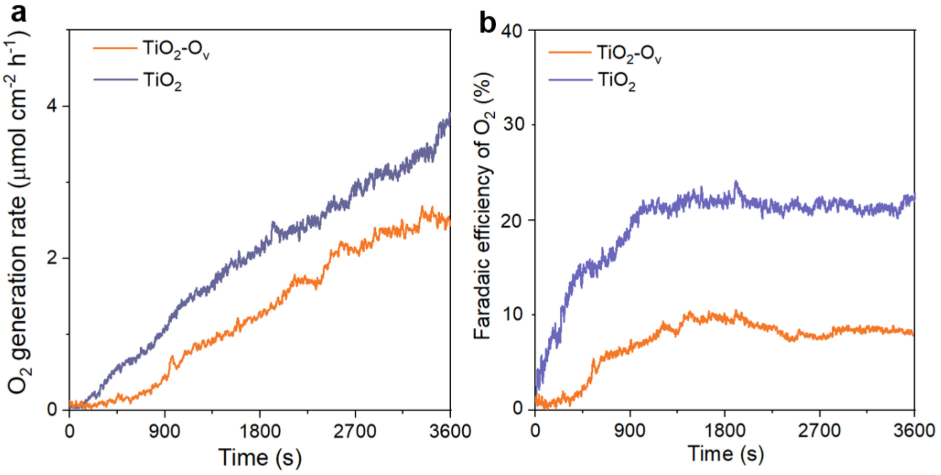

Supplementary Figure 18 | OER side reaction. a, O_2 generation during the PEC cyclohexane chlorination over TiO_2 and TiO_2-O_v-400 photoanodes in 0.5 M NaCl with 18.8 mmol cyclohexane at 1.6 V vs. RHE under AM 1.5G, 100 mW cm^{-2} illumination for 1 h. **b**, the corresponding Faradaic efficiency of O_2 during PEC cyclohexane chlorination over TiO_2 and TiO_2-O_v-400 photoanodes.

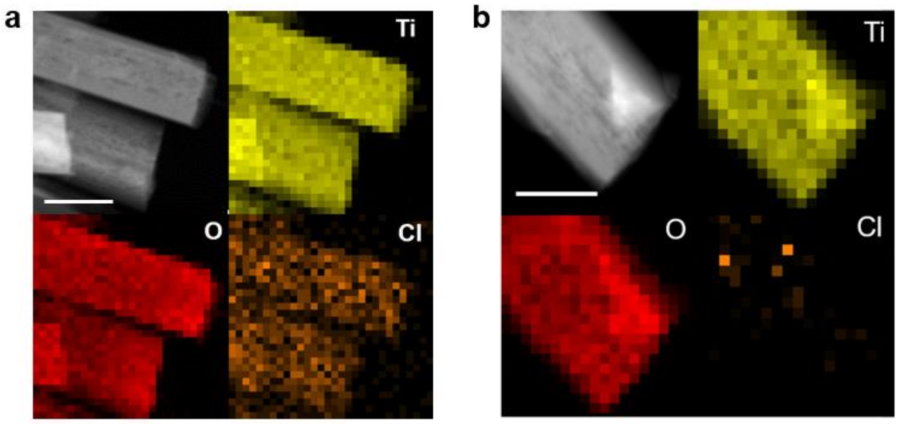

Supplementary Figure 19 | Cl⁻ adsorption over TiO_2-O_v-400. HAADF-STEM-EDS mapping of **a**, TiO_2-O_v-400-Cl and **b**, TiO_2-Cl samples. Scale bars: 100 nm.

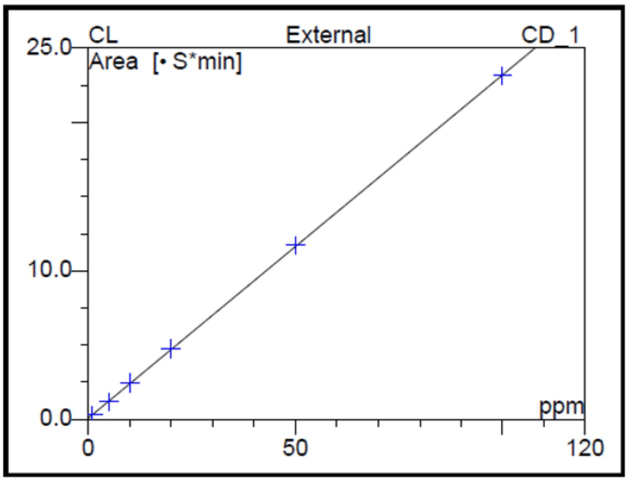

No.	Ret.Time min	Peak Name	Cal.Type	Points	Offset (C0)	Slope (C1)	Curve (C2)	Coeff.Det. %
2	3.72	CL	LOff	6	0.084	0.231	0.000	99.9969
4	5.00	SO4	LOff	6	0.053	0.134	0.000	99.9929
5	5.52	NO3	LOff	6	-0.079	0.129	0.000	99.9876
AVERAGE:					0.0193	0.1647	0.0000	99.9925

Supplementary Figure 20 | Ion chromatography (IC) standard curve line for Cl⁻.

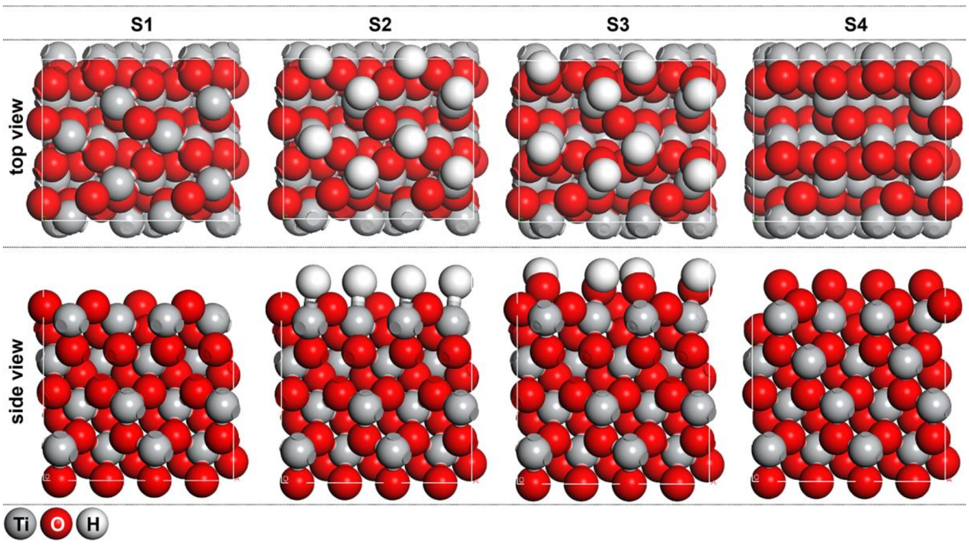

Supplementary Figure 21 | Optimized geometries of models. Optimized geometries of models S1, S2, S3 and S4, terminated with Ti, –H, –OH, and –O, respectively, in the top and side views. The color for each element is labeled.

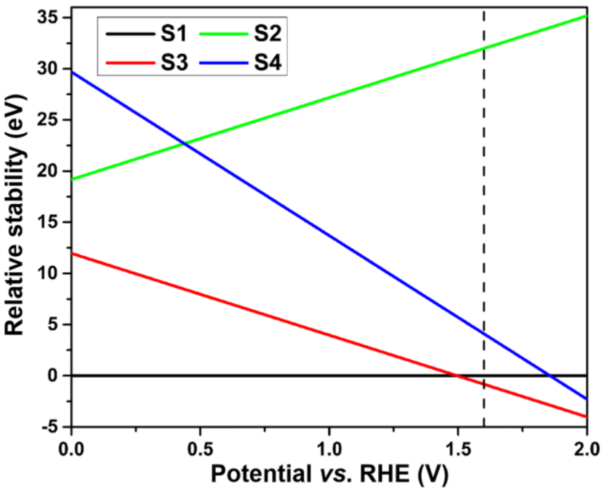

Supplementary Figure 22 | Relative stabilities of models S1, S2, S3 and S4. These models are terminated with Ti, –H, –OH and –O, respectively, under different potentials. The Gibbs free energy of model S1 is set as the zero point. The reaction potential in this work is 1.6 V *vs.* RHE, as labeled with dashed black line.

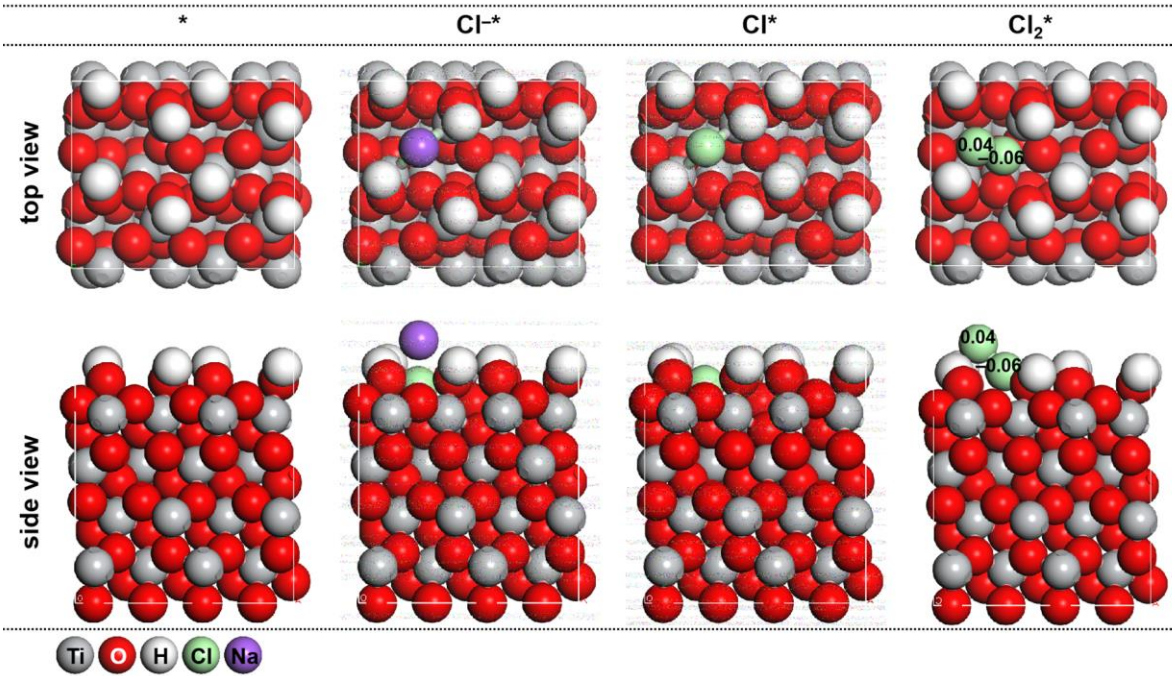

Supplementary Figure 23 | Optimized geometries of reaction intermediates. Optimized geometries of reaction intermediates, *, Cl^{-}*, Cl*, and Cl_2*, for the generation of Cl· and Cl_2 over TiO_2-O_v, in top and side views. The color of each element is labeled. By analyzing the Hirshfeld charge of the Cl_2^* (the top and side views shown in the right column), it is revealed that the atomic charge of Cl atom adsorbed on the oxygen vacancy was calculated to be –0.06 *e*, while the atomic charge of another Cl atom in Cl_2* is 0.04 *e*.

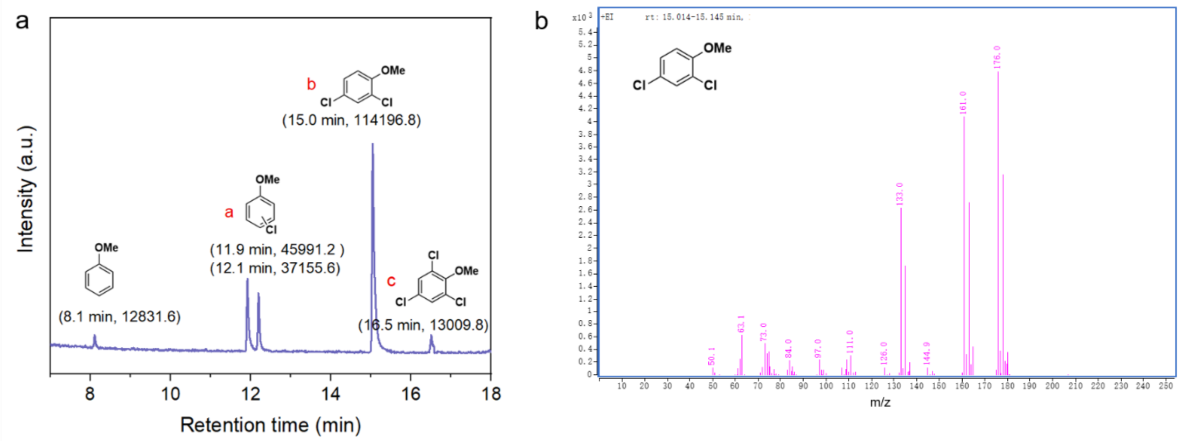

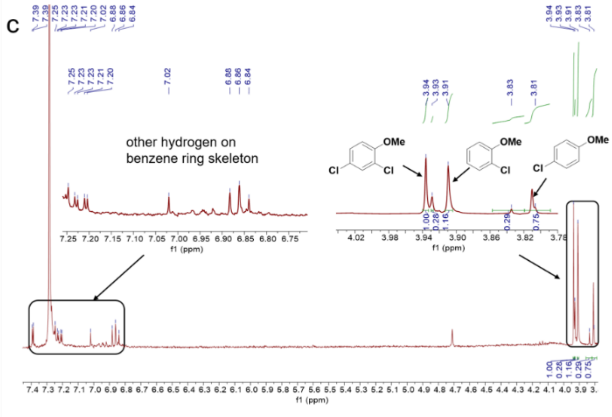

d

Products	Selectivity (%)	Yield (%)
a	37.2(*o*/*p*=1:1.3)	35.0
b	52.6	49.5
c	6.2	5.8

Conversion: 94.2%

Supplementary Figure 24 | PEC chlorination of anisole. a, GC spectrum of the products of PEC chlorination of anisole (compound **1**) on TiO_2-O_V-400 photoanode in 0.5 M NaCl electrolyte with 0.1 mmol anisole at 1.6 V vs. RHE under AM 1.5G, 100 mW cm^{-2} illumination for 10 h. **b**, The MS spectra of product b. **c**, ^{1}H NMR spectrum of the products of PEC chlorination of anisole. **d**, Conversion and selectivity of the products of PEC chlorination of anisole. a.u.: arbitrary units.

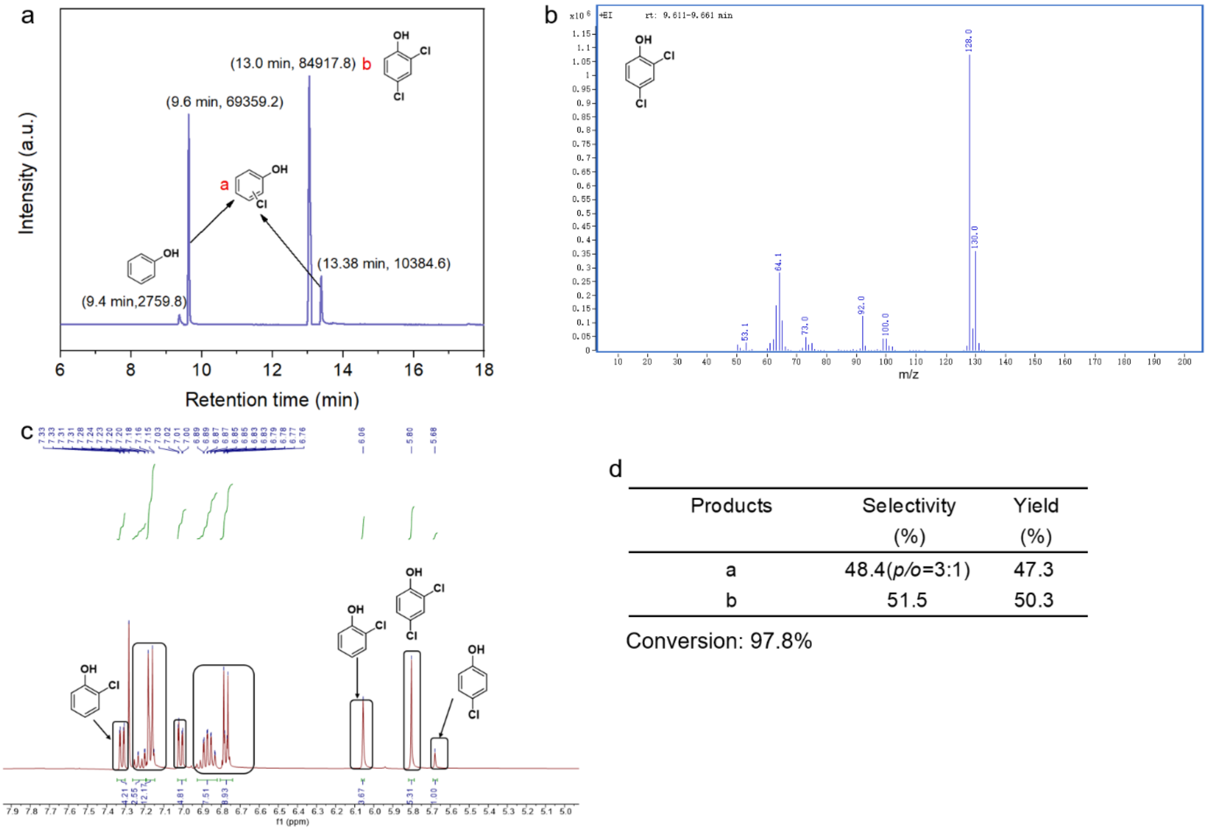

d

Products	Selectivity (%)	Yield (%)
a	48.4(*p*/*o*=3:1)	47.3
b	51.5	50.3

Conversion: 97.8%

Supplementary Figure 25 | PEC chlorination of phenol. a, GC spectrum of the products of PEC chlorination of phenol (compound **2**) on TiO_2-O_V-400 photoanode in 0.5 M NaCl electrolyte with 0.1 mmol phenol dissolved in 0.5 mL acetonitrile (MeCN) at 1.6 V vs. RHE under AM 1.5G, 100 mW cm^{-2} illumination for 10 h. **b**, The MS spectra of product b. **c**, ^1H NMR spectrum of the products of PEC chlorination of phenol. **d**, Conversion and selectivity of PEC chlorination of phenol. a.u.: arbitrary units.

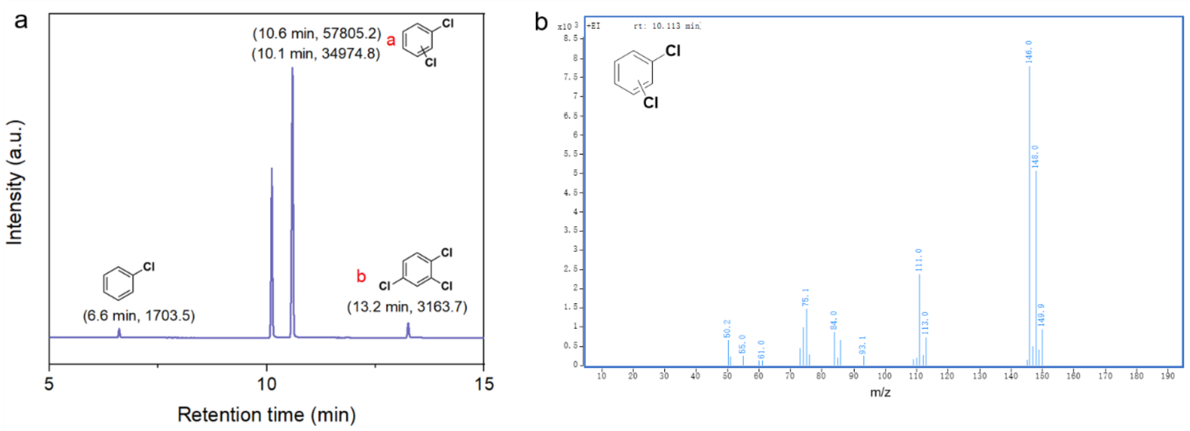

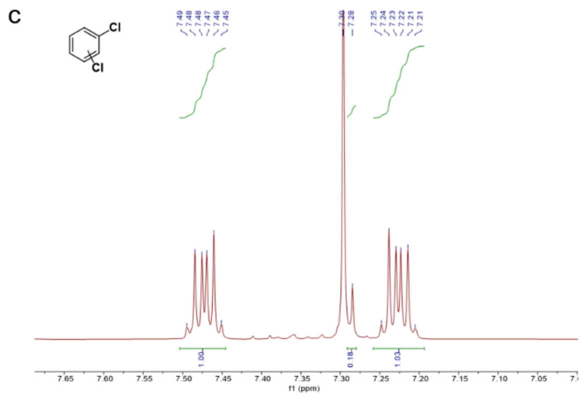

d

Products	Selectivity (%)	Yield (%)
a	95.5(*p*/*o*=1:1)	93.4
b	3.2	3.1

Conversion:97.8%

Supplementary Figure 26 | PEC chlorination of chlorobenzene. a, GC spectrum of the products of PEC chlorination of chlorobenzene (compound **3**) on TiO_2-O_v-400 photoanode in 0.5 M NaCl electrolyte with 0.1 mmol chlorobenzene at 1.6 V vs. RHE under AM 1.5G, 100 mW cm^{-2} illumination for 10 h. **b**, The MS spectra of product a. **c**, ^{1}H NMR spectrum of the products of PEC chlorination of chlorobenzene. **d**, Conversion and selectivity of PEC chlorination of chlorobenzene. a.u.: arbitrary units.

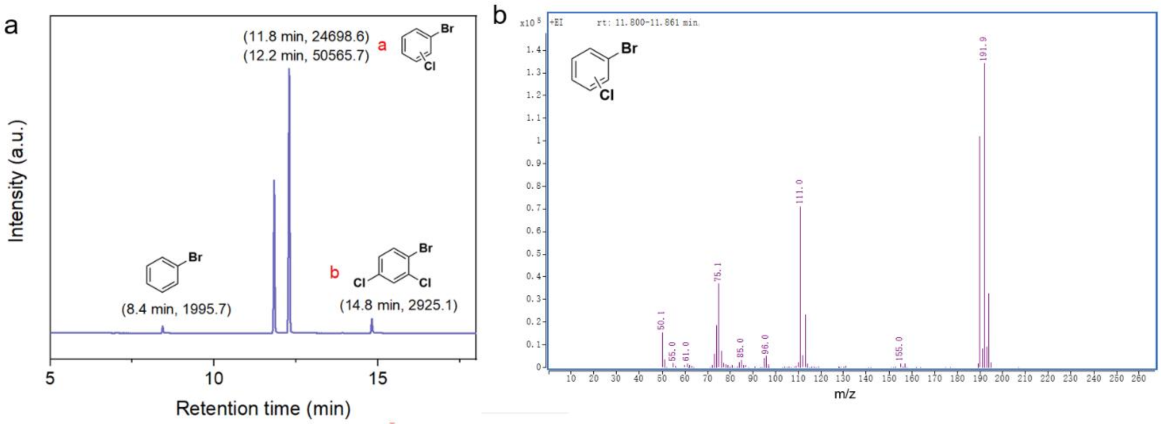

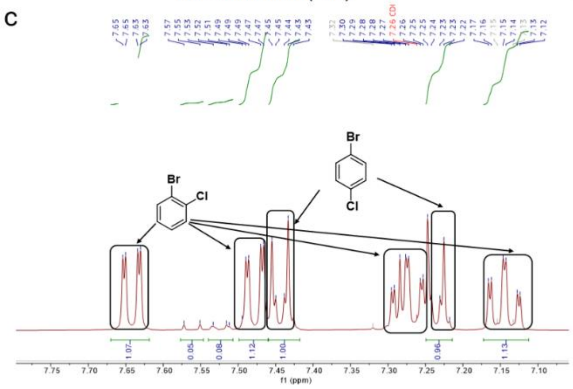

d

Products	Selectivity (%)	Yield (%)
a	93.1(*p*/*o*=1:0.9)	90.7
b	3.7	3.6

Conversion: 97.5%

Supplementary Figure 27 | PEC chlorination of bromobenzene. a, GC spectrum of the products of PEC chlorination of bromobenzene (compound **4**) on TiO_2-O_v-400 photoanode in 0.5 M NaCl electrolyte with 0.1 mmol bromobenzene at 1.6 V vs. RHE under AM 1.5G, 100 mW cm^{-2} illumination for 10 h. **b**, The MS spectra of product a. **c**, ^1H NMR spectrum of the products of PEC chlorination of bromobenzene. **d**, Conversion and selectivity of PEC chlorination of bromobenzene. a.u.: arbitrary units.

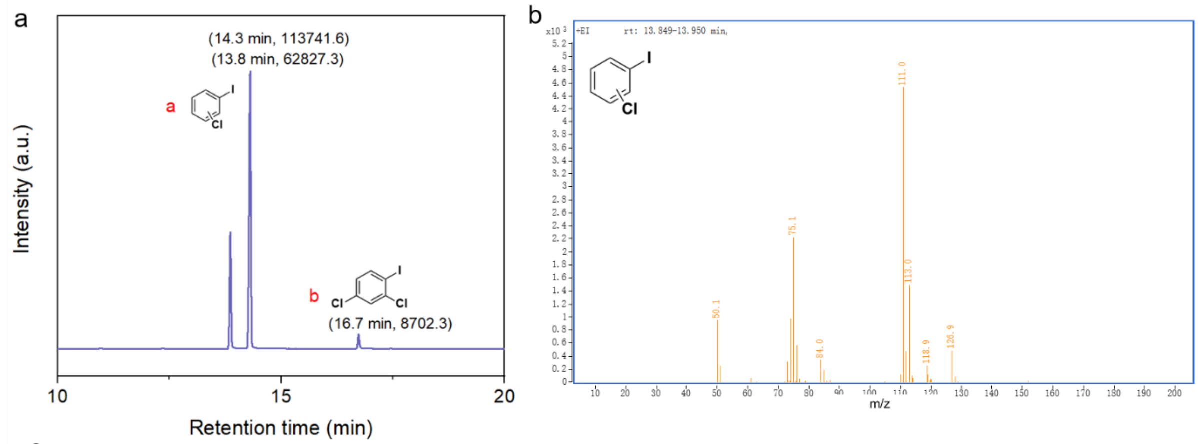

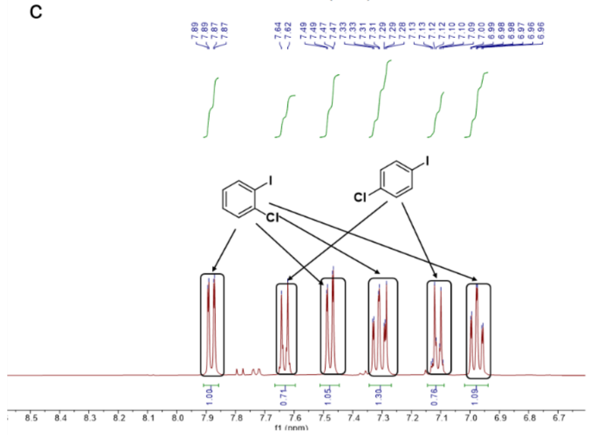

d

Products	Selectivity (%)	Yield (%)
a	93.3(*p*/*o*=1:0.7)	93.3
b	4.7	4.7

Conversion: >99%

Supplementary Figure 28 | PEC chlorination of iodobenzene. a, GC spectrum of the products of PEC chlorination of iodobenzene (compound **5**) on TiO_2-O_v-400 photoanode in 0.5 M NaCl electrolyte with 0.1 mmol iodobenzene at 1.6 V vs. RHE under AM 1.5G, 100 mW cm^{-2} illumination for 10 h. **b**, The MS spectra of product a. **c**, ^{1}H NMR spectrum of the products of PEC chlorination of iodobenzene. **d**, Conversion and selectivity of PEC chlorination of iodobenzene. a.u.: arbitrary units.

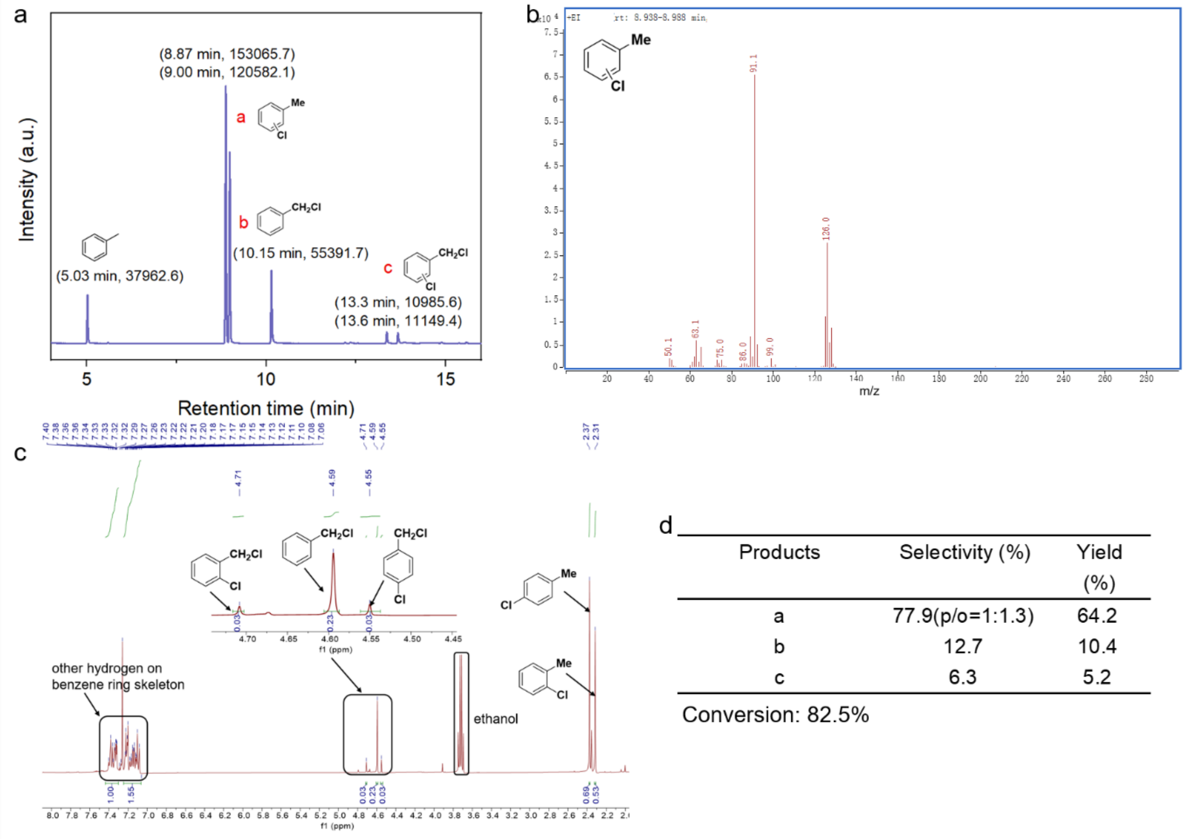

d

Products	Selectivity (%)	Yield (%)
a	77.9(p/o=1:1.3)	64.2
b	12.7	10.4
c	6.3	5.2

Conversion: 82.5%

Supplementary Figure 29 | PEC chlorination of toluene. a, GC spectrum of the products of PEC chlorination of toluene (compound **6**) on TiO_2-O_v-400 photoanode in 0.5 M NaCl electrolyte with 0.1 mmol toluene at 1.6 V vs. RHE under AM 1.5G, 100 mW cm^{-2} illumination for 10 h. **b**, The MS spectra of product a. **c**, ^1H NMR spectrum of the products of PEC chlorination of toluene. **d**, Conversion and selectivity of PEC chlorination of toluene. a.u.: arbitrary units.

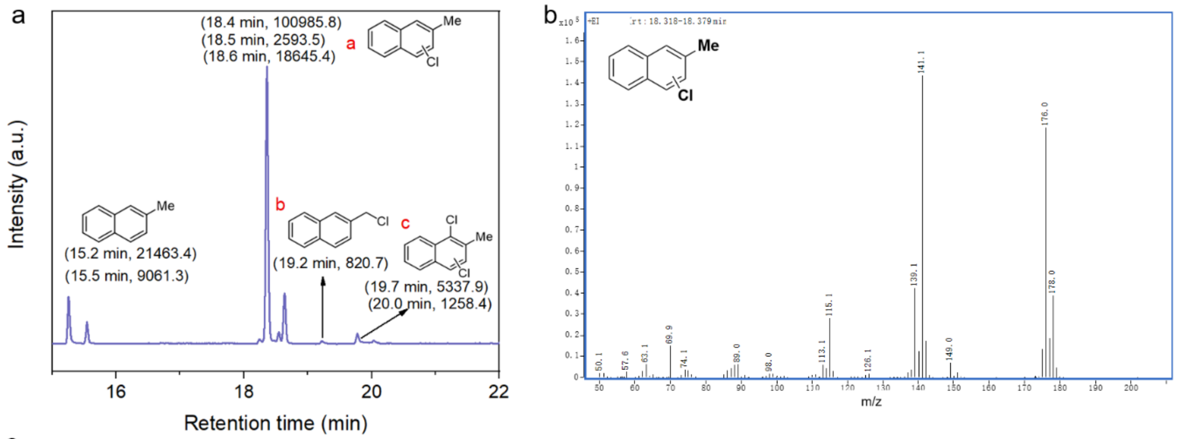

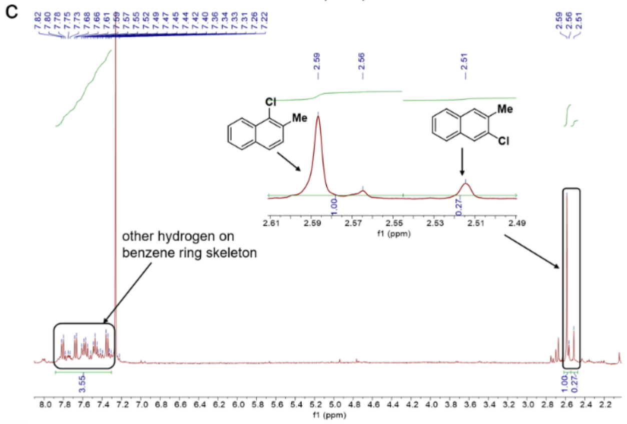

d

Products	Selectivity (%)	Yield (%)
a	90.2(1-Cl/3-Cl=1:0.27)	73.8
b	0.6	0.5
c	5.0	4.0

Conversion: 81.9%

Supplementary Figure 30 | PEC chlorination of naphthalene. a, GC spectrum of the products of PEC chlorination of methyl naphthalene (compound **7**) on TiO_2-O_V-400 photoanode in 0.5 M NaCl electrolyte with 0.1 mmol methyl naphthalene at 1.6 V vs. RHE under AM 1.5G, 100 mW cm^{-2} illumination for 10 h. **b**, The MS spectra of product a. **c**, ^{1}H NMR spectrum of the products of PEC chlorination of methyl naphthalene. **d**, Conversion and selectivity of PEC chlorination of methyl naphthalene. a.u.: arbitrary units.

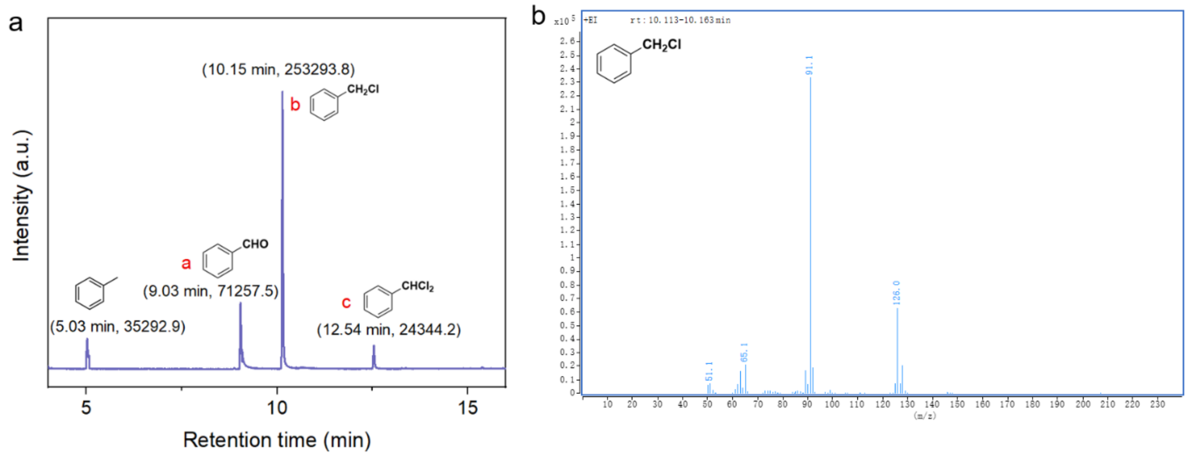

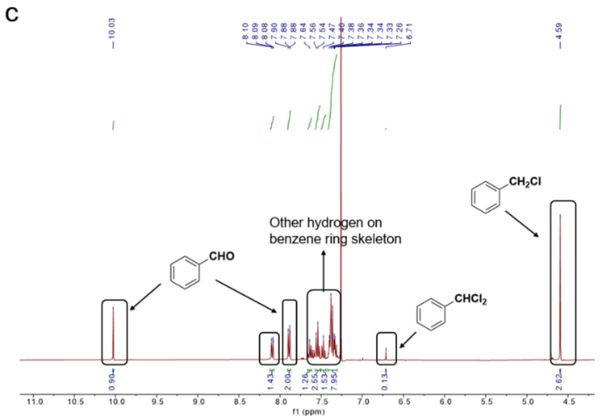

d

Products	Selectivity (%)	Yield (%)
a	15.4	12.8
b	75.5	62.8
c	4.8	3.8

Conversion: 83.2%

Supplementary Fig. 31 | PEC chlorination of toluene. a, GC spectra of the products of PEC chlorination of toluene (0.1 mmol) on TiO_2 photoanode in 0.5 M NaCl electrolyte at 1.6 V vs. RHE under AM 1.5G, 100 mW cm^{-2} illumination for 10 h. **b**, The MS spectra of product b. **c**, Corresponding ^{1}H NMR spectra of the products of PEC chlorination of toluene. **d**, Conversion and selectivity of PEC chlorination of toluene. a.u.: arbitrary units.

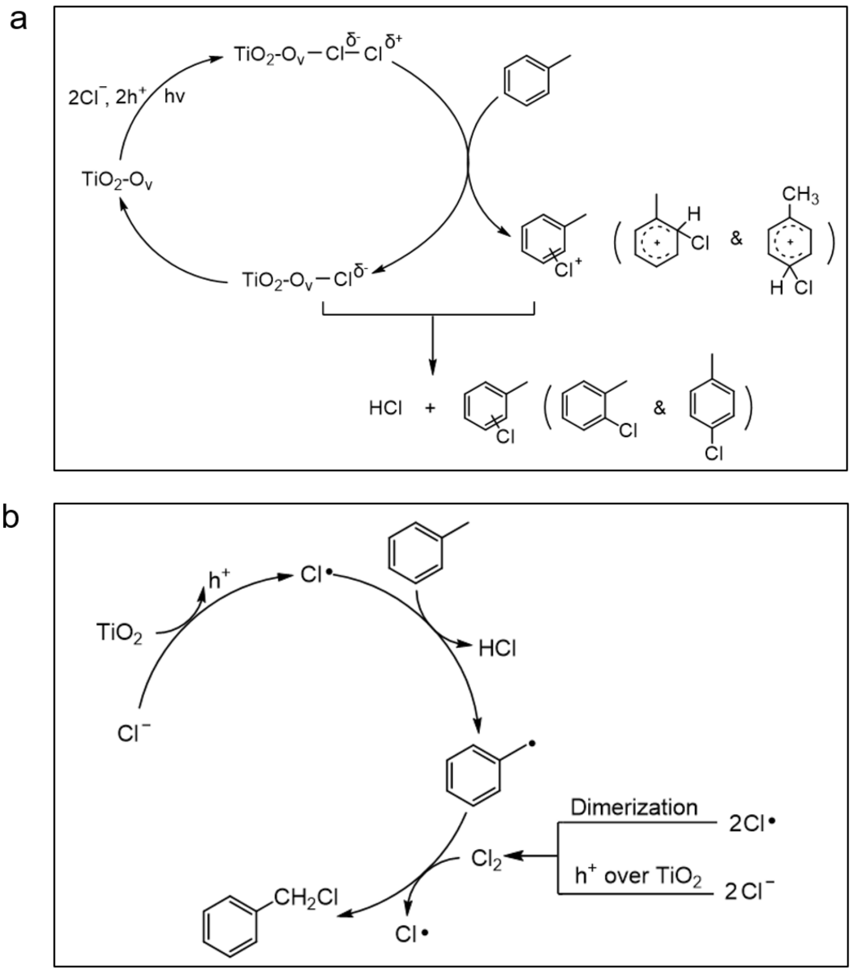

Supplementary Fig. 32 | Mechanism of PEC toluene chlorination. a, Proposed mechanism of PEC chlorination of toluene over TiO_2-O_v-400 photoanode. **b**, Proposed mechanism of PEC chlorination of toluene over TiO_2 photoanode.

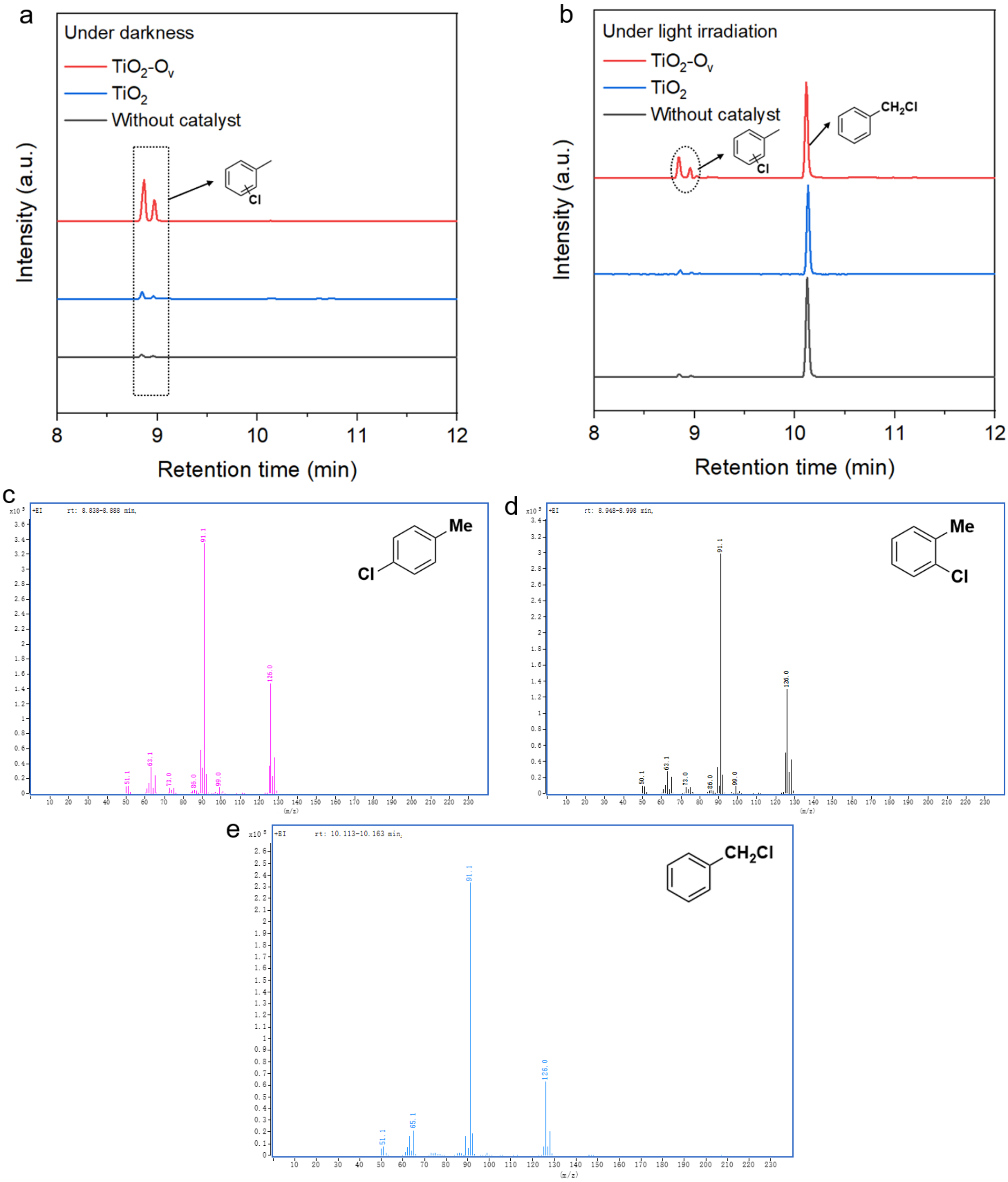

Supplementary Fig. 33 | Chlorination of toluene with Cl_2. a, GC spectra of the products of chlorination of toluene (5 mmol) in aqueous solution used Cl_2 (1 mL) as the chlorine source under darkness for 15 min. **b**, GC spectra of the products of chlorination of toluene (5 mmol) in aqueous solution used Cl_2 (1 mL) as the chlorine source under AM 1.5G, 100 mW cm^{-2} illumination for 15 min. **c-e**, MS spectra of standard samples (*o*-chlorotoluene (8.8 min), *p*-chlorotoluene (8.9 min) and benzyl chloride (10.1 min).

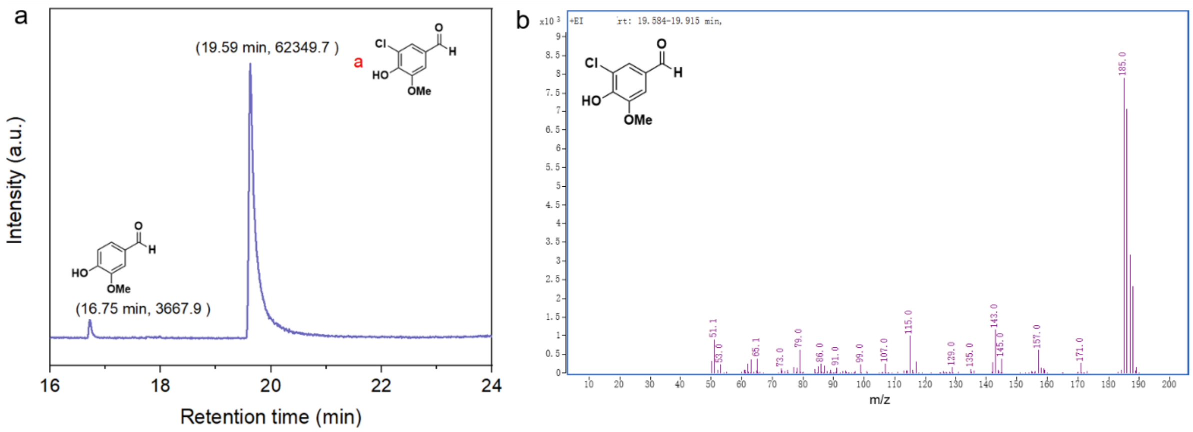

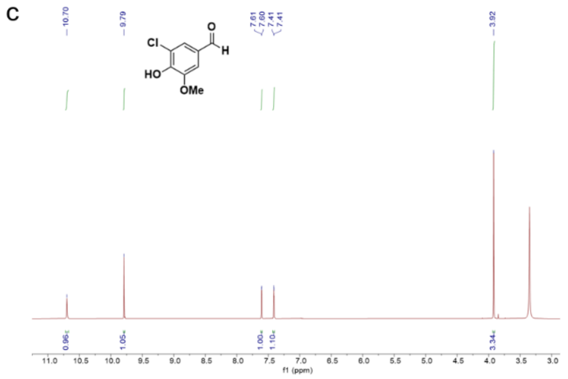

d

Products	Selectivity (%)	Yield (%)
a	>99	95.0

Conversion: 95.0%

Supplementary Fig. 34 | PEC chlorination of vanillin. a, GC spectrum of the products of PEC chlorination of vanillin (compound **8**) on TiO_2-O_v-400 photoanode in 0.5 M NaCl electrolyte with 0.1 mmol phenol dissolved in 0.5 mL acetonitrile (MeCN) at 1.6 V vs. RHE under AM 1.5G, 100 mW cm^{-2} illumination for 10 h. **b**, The MS spectra of product a. **c**, Corresponding ^{1}H NMR spectra of the products of PEC chlorination of vanillin. **d**, Conversion and selectivity of PEC chlorination of vanillin. a.u.: arbitrary units.

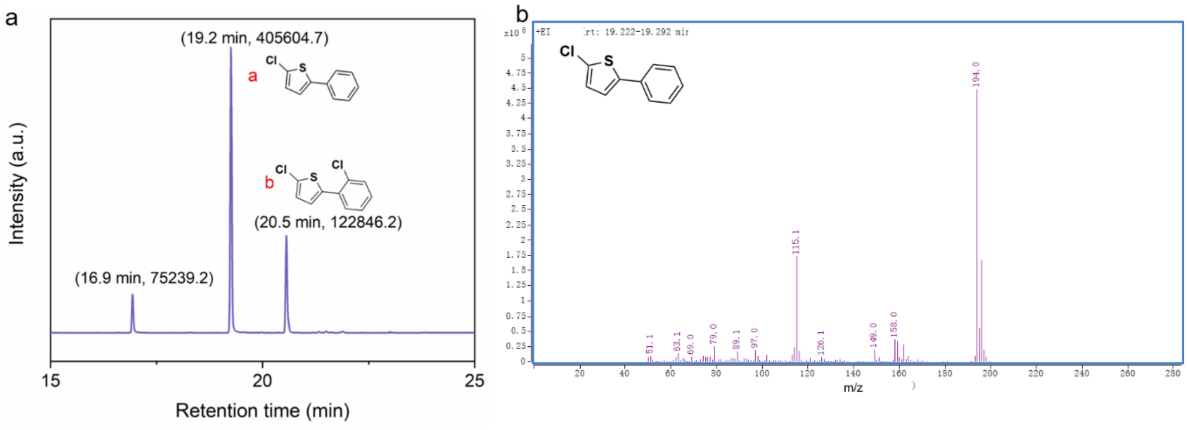

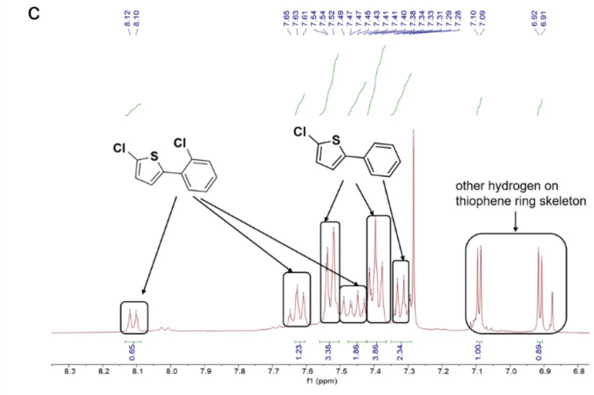

d

Products	Selectivity (%)	Yield (%)
a	75.9	70.2
b	22.8	21.1

Conversion: 92.5%

Supplementary Figure 35 | PEC chlorination of O-phenylthiophene. a, GC spectrum of the products of PEC chlorination of O-phenylthiophene (compound **9**) on TiO_2-O_v-400 photoanode in 0.5 M NaCl electrolyte with 0.1 mmol O-phenylthiophene dissolved in 0.5 mL MeCN at 1.6 V vs. RHE under AM 1.5G, 100 mW cm^{-2} illumination for 10 h. **b**, The MS spectra of product a. **c**, ^{1}H NMR spectrum of the products of PEC chlorination of O-phenylthiophene. **d**, Conversion and selectivity of PEC chlorination of O-phenylthiophene. a.u.: arbitrary units.

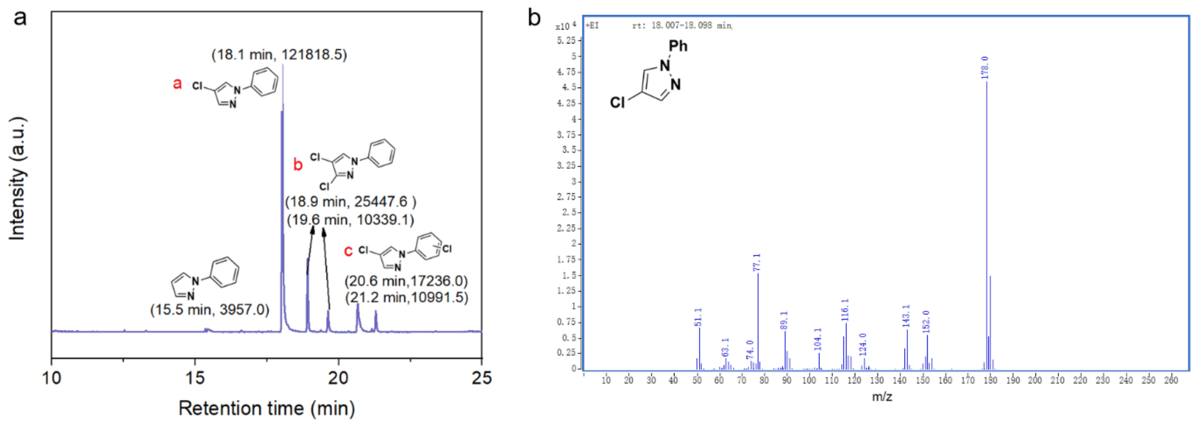

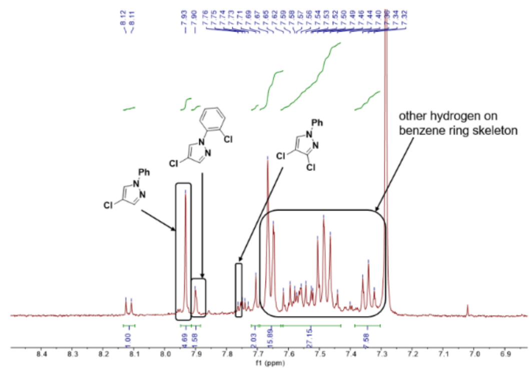

d

Products	Selectivity (%)	Yield (%)
a	65.6	63.7
b	19.2	18.3
c	13.6	13.2

Conversion: 97.2%

Supplementary Figure 36 | PEC chlorination of 1-phenyl-1H-pyrazole. a, GC spectrum of the products of PEC chlorination of 1-phenyl-1H-pyrazole (compound **10**) on TiO_2-O_v-400 photoanode in 0.5 M NaCl electrolyte with 0.1 mmol 1-phenyl-1H-pyrazole at 1.6 V vs. RHE under AM 1.5G, 100 mW cm^{-2} illumination for 10 h. **b**, The MS spectra of product a. **c**, ^{1}H NMR spectrum of the products of PEC chlorination of 1-phenyl-1H-pyrazole. **d**, Conversion and selectivity of PEC chlorination of 1-phenyl-1H-pyrazole. a.u.: arbitrary units.

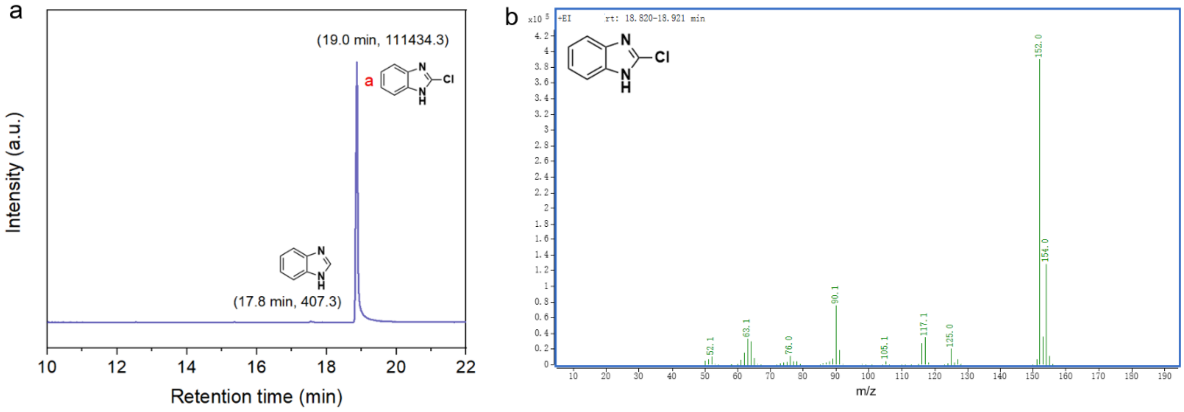

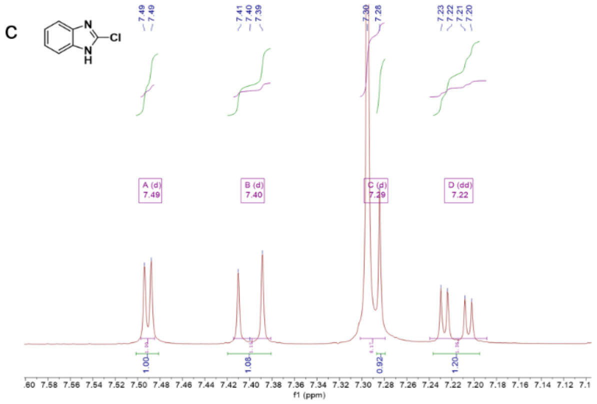

d

Products	Selectivity (%)	Yield (%)
a	>99	>99

Conversion: >99%

Supplementary Figure 37 | PEC chlorination of 1H-benzo[d]imidazole. a, GC spectrum of the products of PEC chlorination of 1H-benzo[d]imidazole (compound **11**) on TiO_2-O_v-400 photoanode in 0.5 M NaCl electrolyte with 0.1 mmol 1H-benzo[d]imidazole dissolved in 0.5 mL MeCN at 1.6 V vs. RHE under AM 1.5G, 100 mW cm^{-2} illumination for 10 h. **b**, The MS spectra of product a. **c**, ^{1}H NMR spectrum of the products of PEC chlorination of 1H-benzo[d]imidazole. **d**, Conversion and selectivity of PEC chlorination of 1H-benzo[d]imidazole. a.u.: arbitrary units.

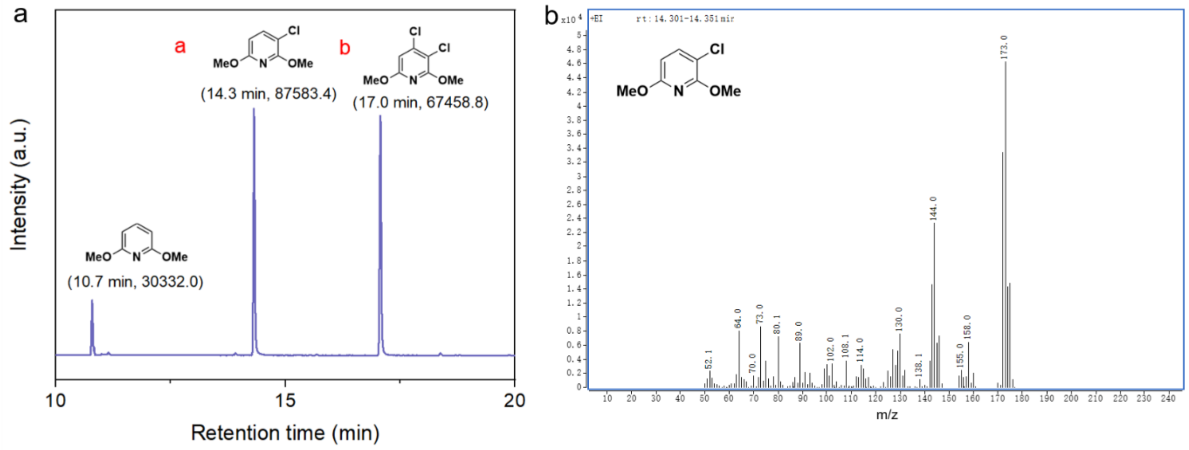

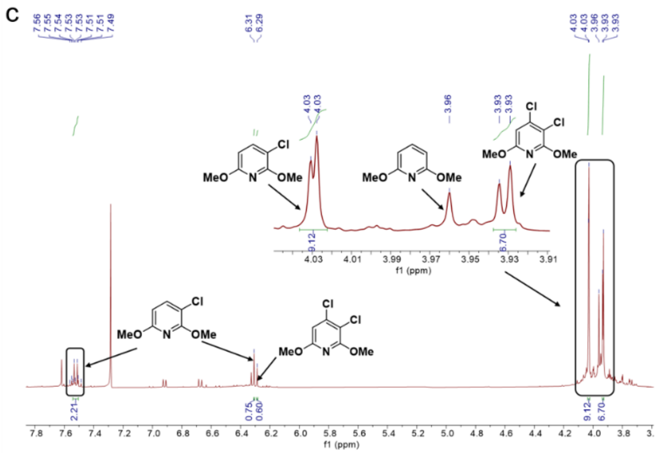

d

Products	Selectivity (%)	Yield (%)
a	56.4	47.1
b	42.3	36.5

Conversion: 83.6%

Supplementary Figure 38 | PEC chlorination of 2,6-dimethoxypyridine. a, GC spectrum of the products of PEC chlorination of 2,6-dimethoxypyridine (compound **12**) on TiO_2-O_v-400 photoanode in 0.5 M NaCl electrolyte with 0.1 mmol 2,6-dimethoxypyridine at 1.6 V vs. RHE under AM 1.5G, 100 mW cm^{-2} illumination for 10 h. **b**, The MS spectra of product a. **c**, ^{1}H NMR spectrum of the products of PEC chlorination of 2,6-dimethoxypyridine. **d**, Conversion and selectivity of PEC chlorination of 2,6-dimethoxypyridine. a.u.: arbitrary units.

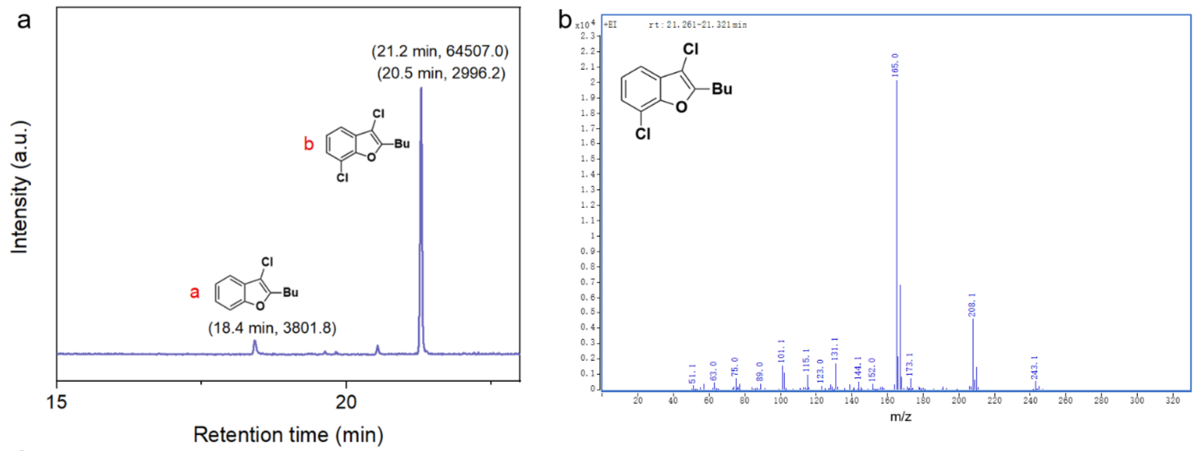

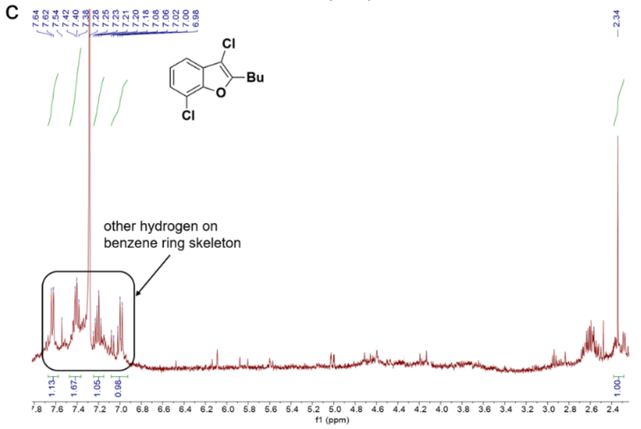

d

Products	Selectivity (%)	Yield (%)
a	5.3	5.3
b	93.3	93.3

Conversion: >99%

Supplementary Figure 39 | PEC chlorination of 2-butylbenzofuran. a, GC spectrum of the products of PEC chlorination of 2-butylbenzofuran (compound **13**) on TiO_2-O_v-400 photoanode in 0.5 M NaCl electrolyte with 0.1 mmol 2-butylbenzofuran dissolved in 1 mL MeCN at 1.6 V vs. RHE under AM 1.5G, 100 mW cm^{-2} illumination for 10 h. **b**, The MS spectra of product b. **c**, ^1H NMR spectrum of the products of PEC chlorination of 2-butylbenzofuran. **d**, Conversion and selectivity of PEC chlorination of 2-butylbenzofuran. a.u.: arbitrary units.

a

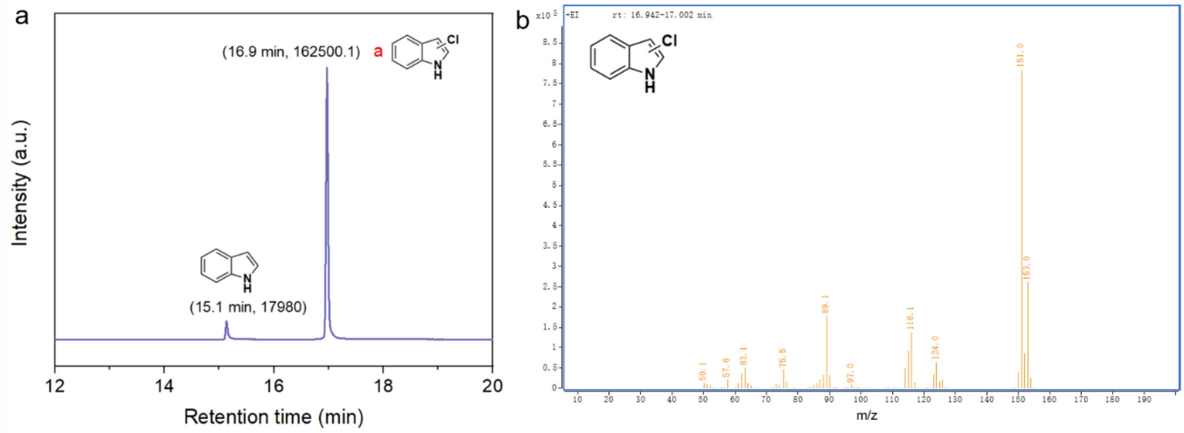

c

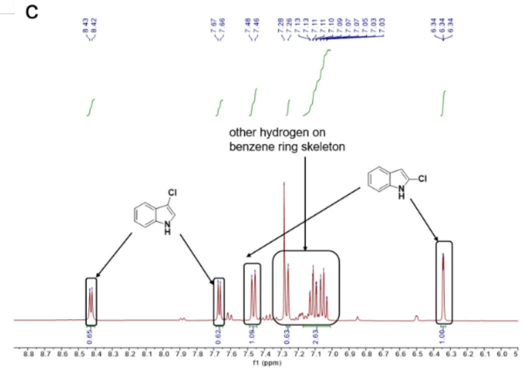

d

Products	Selectivity (%)	Yield (%)
a	>99(2-Cl/3-Cl=1:0.6)	90

Conversion: 90.0%

Supplementary Figure 40 | PEC chlorination of 1H-indole. a, GC spectrum of the products of PEC chlorination of 1H-indole (compound **14**) on TiO_2-O_V-400 photoanode in 0.5 M NaCl electrolyte with 0.1 mmol 1H-indole dissolved in 0.5 mL MeCN at 1.6 V vs. RHE under AM 1.5G, 100 mW cm^{-2} illumination for 10 h. **b**, The MS spectra of product a. **c**, ^{1}H NMR spectrum of the products of PEC chlorination of 1H-indole. **d**, Conversion and selectivity of PEC chlorination of 1H-indole. a.u.: arbitrary units.

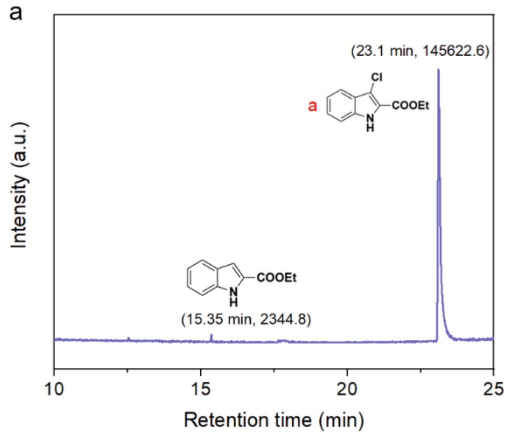

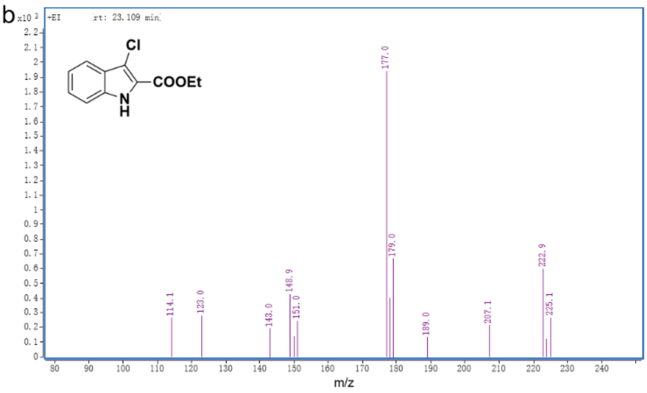

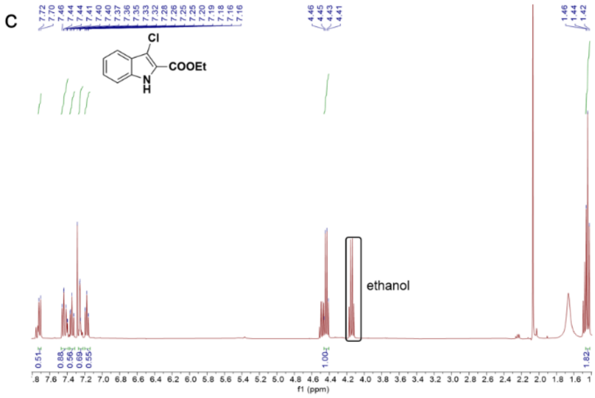

d

Products	Selectivity (%)	Yield (%)
a	>99	94.7

Conversion: 94.7%

Supplementary Figure 41 | PEC chlorination of 1H-indole-2-carboxylate. a, GC spectrum of the products of PEC chlorination of 1H-indole-2-carboxylate (compound **15**) on TiO_2-O_v-400 photoanode in 0.5 M NaCl electrolyte with 0.1 mmol ethyl 1H-indole-2-carboxylate dissolved in 0.5 mL MeCN at 1.6 V vs. RHE under AM 1.5G, 100 mW cm^{-2} illumination for 10 h. **b**, The MS spectra of product a. **c**, ^{1}H NMR spectrum of the products of PEC chlorination of 1H-indole-2-carboxylate. **d**, Conversion and selectivity of PEC chlorination of 1H-indole-2-carboxylate. a.u.: arbitrary units.

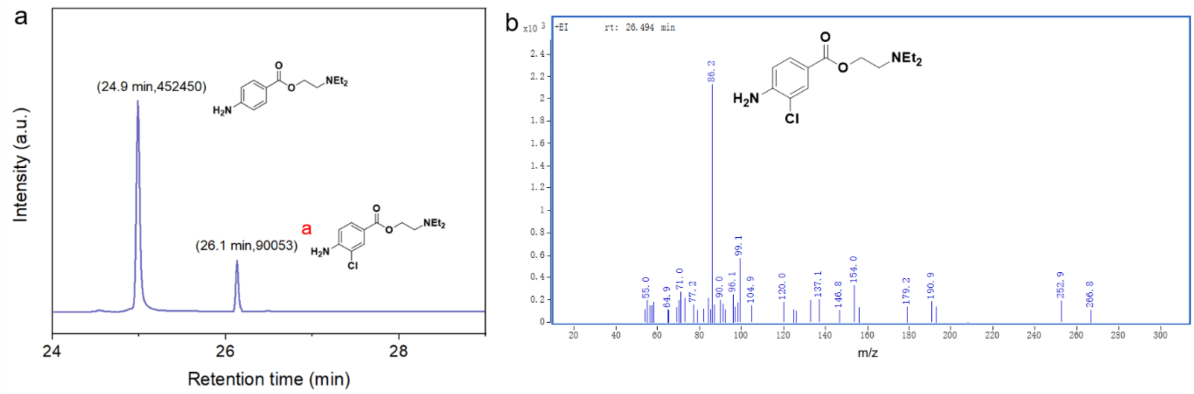

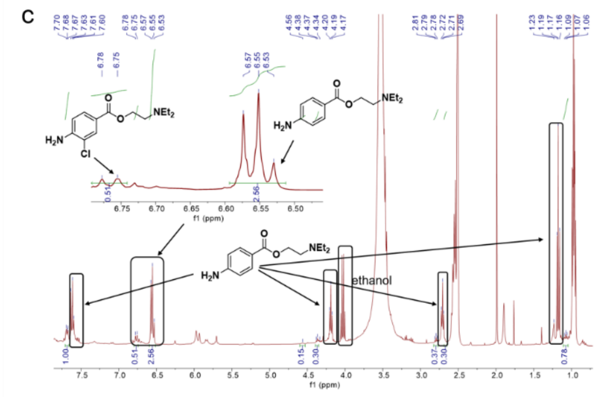

d

Products	Selectivity (%)	Yield (%)
a	>99	23.2

Conversion: 23.2%

Supplementary Figure 42 | PEC chlorination of procaine. a, GC spectrum of the products of PEC chlorination of procaine (compound **16**) on TiO_2-O_v-400 photoanode in 0.5 M NaCl electrolyte with 0.1 mmol procaine dissolved in 0.5 mL MeCN at 1.6 V vs. RHE under AM 1.5G, 100 mW cm^{-2} illumination for 10 h. **b**, The MS spectra of product a. **c**, ^{1}H NMR spectrum of the products of PEC chlorination of procaine. **d**, Conversion and selectivity of PEC chlorination of procaine. a.u.: arbitrary units.

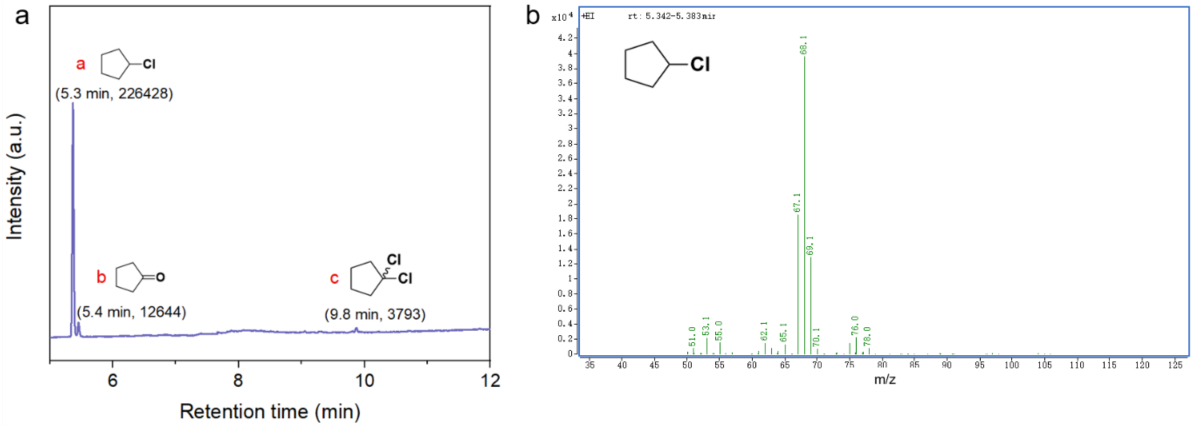

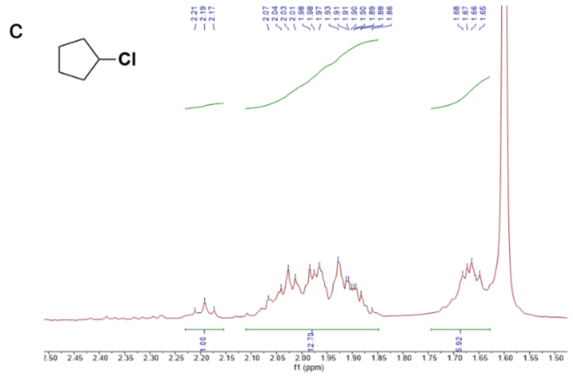

d

Products	Selectivity (%)	Yield (%)
a	87.4	78.8
b	5.7	5.1
c	2.8	2.5

Conversion: 90.3%

Supplementary Figure 43 | PEC chlorination of cyclopentane. a, GC spectrum of the products of PEC chlorination of cyclopentane (compound **17**) on TiO_2-O_v-400 photoanode in 0.5 M NaCl electrolyte with 0.1 mmol cyclopentane at 1.6 V vs. RHE under AM 1.5G, 100 mW cm^{-2} illumination for 10 h. **b**, The MS spectra of product a. **c**, ^{1}H NMR spectrum of the products of PEC chlorination of cyclopentane. **d**, Conversion and selectivity of PEC chlorination of cyclopentane. a.u.: arbitrary units.

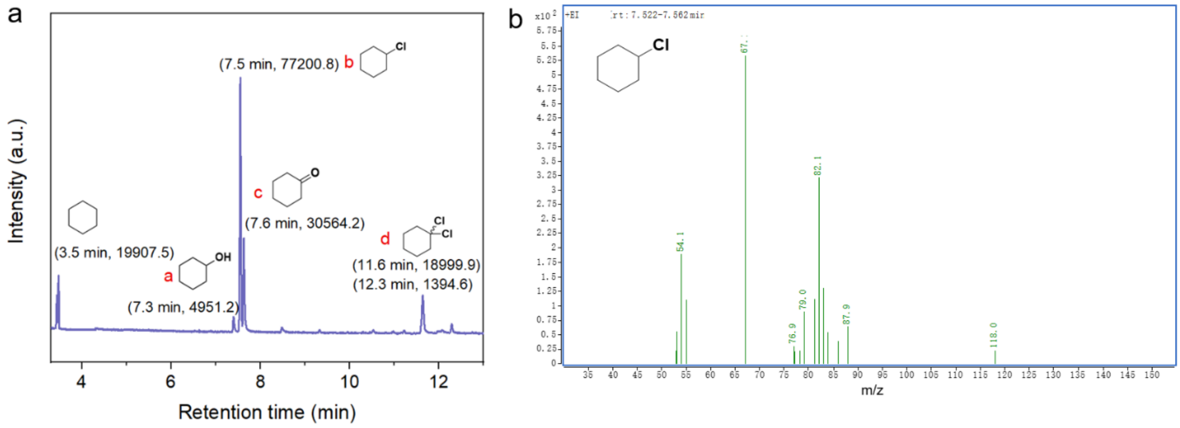

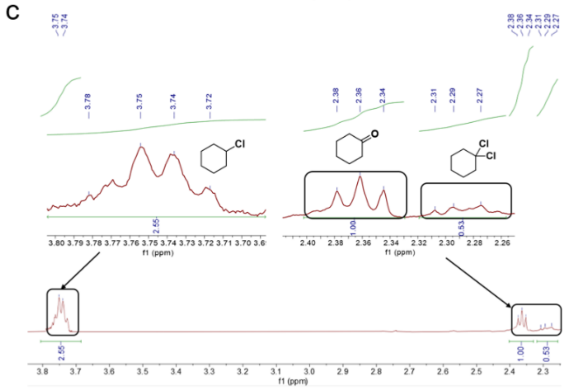

d

Products	Selectivity (%)	Yield (%)
a	3.7	3.2
b	54.9	47.7
c	22.9	19.9
d	14.8	12.8

Conversion: 88.9%

Supplementary Figure 44 | PEC chlorination of cyclohexane. a, GC spectrum of the products of PEC chlorination of cyclohexane (compound **18**) on TiO_2-O_v-400 photoanode in 0.5 M NaCl electrolyte with 0.1 mmol cyclohexane at 1.6 V vs. RHE under AM 1.5G, 100 mW cm^{-2} illumination for 10 h. **b**, The MS spectra of product b. **c**, ^{1}H NMR spectrum of the products of PEC chlorination of cyclohexane. **d**, Conversion and selectivity of PEC chlorination of cyclohexane. a.u.: arbitrary units.

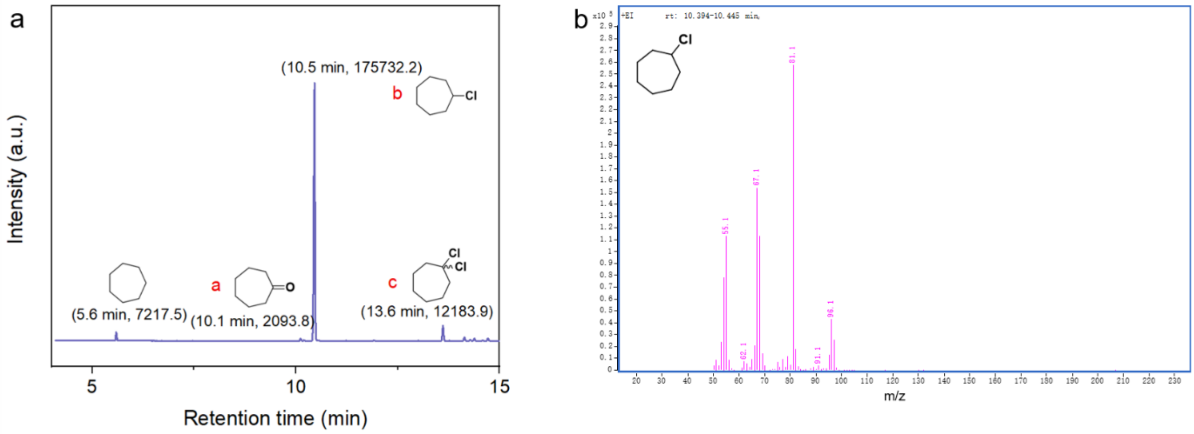

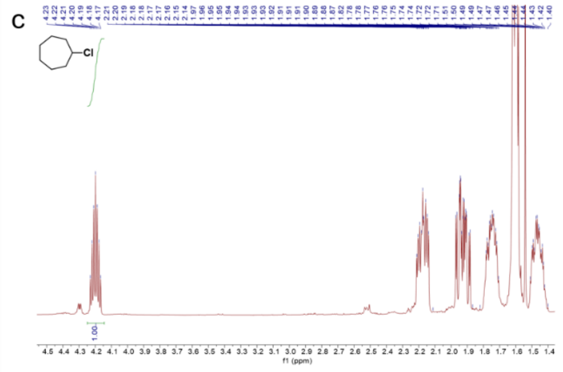

d

Products	Selectivity (%)	Yield (%)
a	12.2	11.2
b	69.2	63.8
c	7.4	6.8

Conversion: 92.3%

Supplementary Figure 45 | PEC chlorination of cycloheptane. a, GC spectrum of the products of PEC chlorination of cycloheptane (compound **19**) on TiO_2-O_V-400 photoanode in 0.5 M NaCl electrolyte with 0.1 mmol cycloheptane at 1.6 V vs. RHE under AM 1.5G, 100 mW cm^{-2} illumination for 10 h. **b**, The MS spectra of product b. **c**, ^1H NMR spectrum of the products of PEC chlorination of cycloheptane. **d**, Conversion and selectivity of PEC chlorination of cycloheptane. a.u.: arbitrary units.

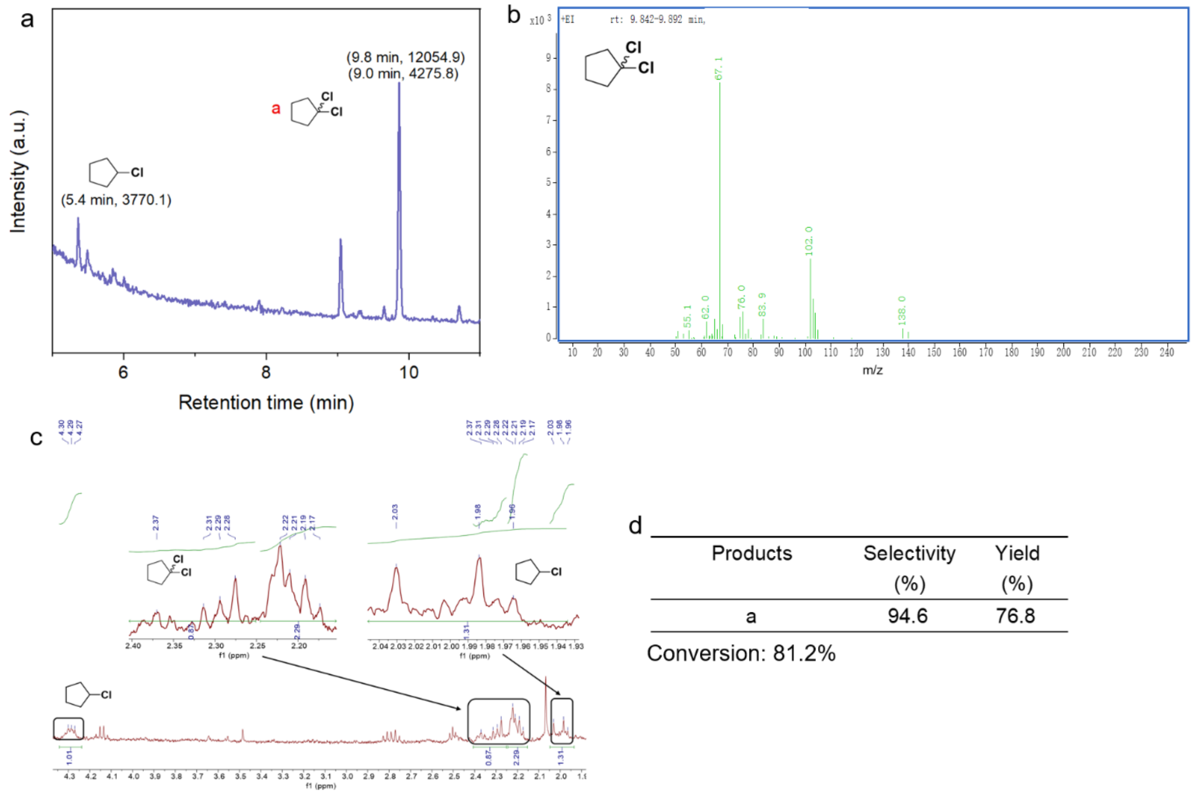

d

Products	Selectivity (%)	Yield (%)
a	94.6	76.8

Conversion: 81.2%

Supplementary Figure 46 | PEC chlorination of chlorocyclopentane. a, GC spectrum of the products of PEC chlorination of chlorocyclopentane (compound **20**) on TiO_2-O_v-400 photoanode in 0.5 M NaCl electrolyte with 0.1 mmol chlorocyclopentane at 1.6 V vs. RHE under AM 1.5G, 100 mW cm^{-2} illumination for 10 h. **b**, The MS spectra of product a. **c**, ^1H NMR spectrum of the products of PEC chlorination of chlorocyclopentane. **d**, Conversion and selectivity of PEC chlorination of chlorocyclopentane. a.u.: arbitrary units.

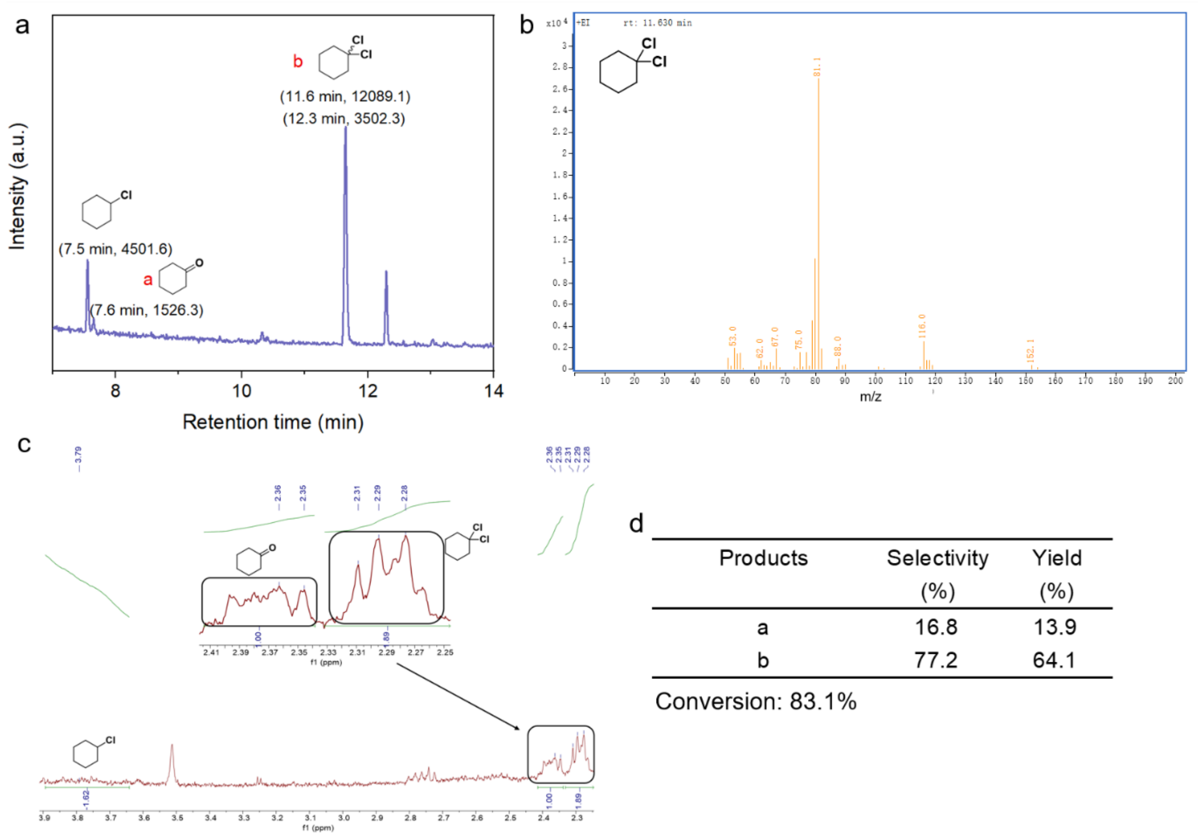

Products	Selectivity (%)	Yield (%)
a	16.8	13.9
b	77.2	64.1

Conversion: 83.1%

Supplementary Figure 47 | PEC chlorination of chlorocyclohexane. a, GC spectrum of the products of PEC chlorination of chlorocyclohexane (compound **21**) on TiO_2-O_V-400 photoanode in 0.5 M NaCl electrolyte with 0.1 mmol chlorocyclohexane at 1.6 V vs. RHE under AM 1.5G, 100 mW cm^{-2} illumination for 100 h. **b**, The MS spectra of product b. **c**, ^{1}H NMR spectrum of the products of PEC chlorination of chlorocyclohexane. **d**, Conversion and selectivity of PEC chlorination of chlorocyclohexane. a.u.: arbitrary units.

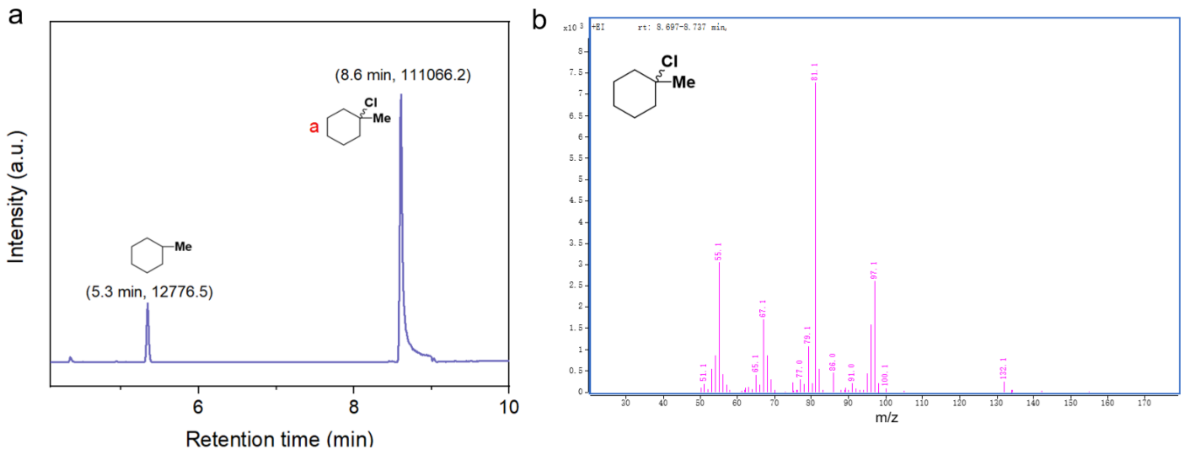

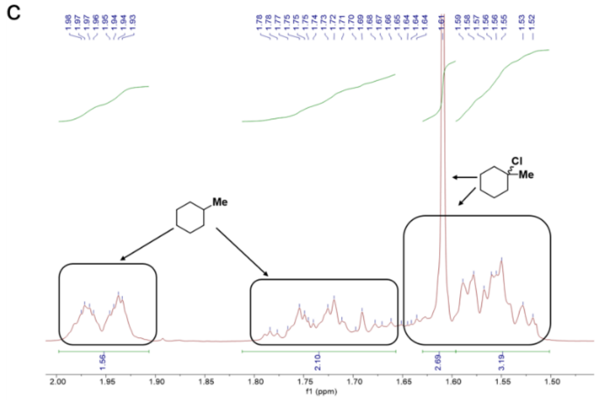

d

Products	Selectivity (%)	Yield (%)
a	89.4	81.9

Conversion: 90.4%

Supplementary Figure 48 | PEC chlorination of methylcyclohexane. a, GC spectrum of the products of PEC chlorination of methylcyclohexane (compound **22**) on TiO_2-O_v-400 photoanode in 0.5 M NaCl electrolyte with 0.1 mmol methylcyclohexane at 1.6 V vs. RHE under AM 1.5G, 100 mW cm^{-2} illumination for 10 h. **b**, The MS spectra of product a. **c**, ^1H NMR spectrum of the products of PEC chlorination of methylcyclohexane. **d**, Conversion and selectivity of PEC chlorination of methylcyclohexane. a.u.: arbitrary units.

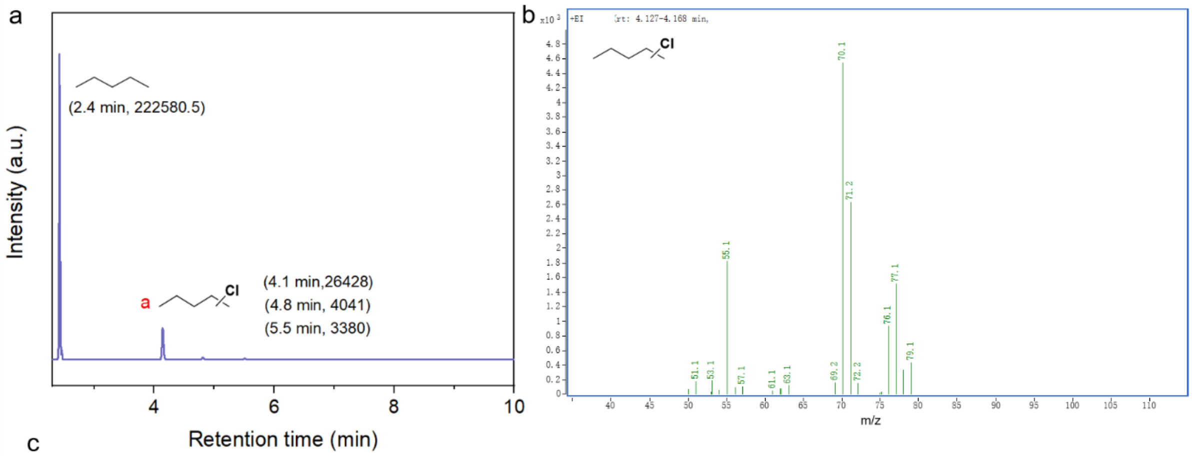

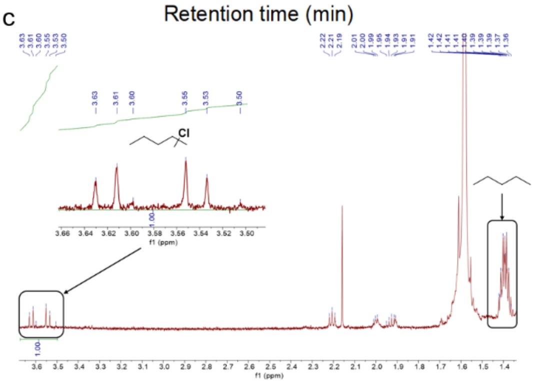

d

Products	Selectivity (%)	Yield (%)
a	94.5	8.3

Conversion: 8.8%

Supplementary Figure 49 | PEC chlorination of pentane. a, GC spectrum of the products of PEC chlorination of pentane (compound **23**) on TiO_2-O_v-400 photoanode in 0.5 M NaCl electrolyte with 20 mmol pentane at 1.6 V vs. RHE under AM 1.5G, 100 mW cm^{-2} illumination for 2 h. **b**, The MS spectra of product a. **c**, ^{1}H NMR spectrum of the products of PEC chlorination of pentane. **d**, Conversion and selectivity of PEC chlorination of pentane. a.u.: arbitrary units.

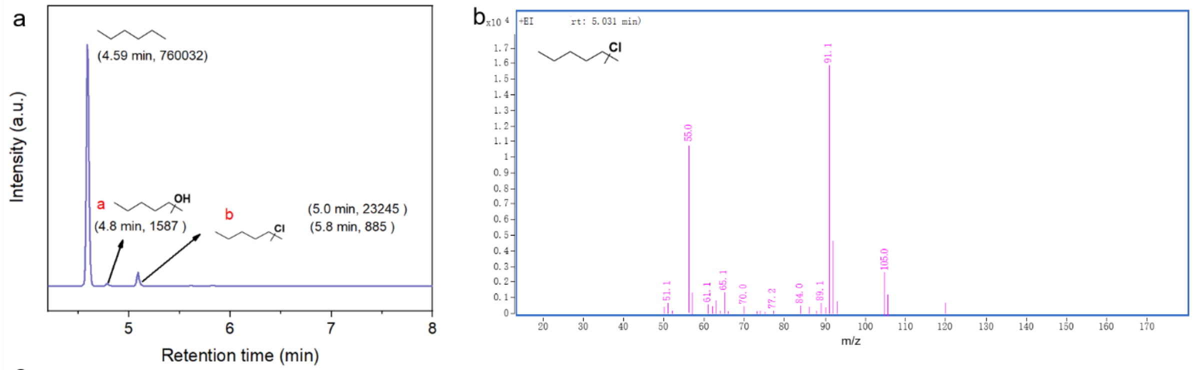

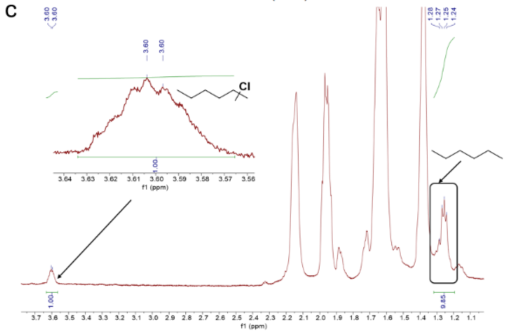

d

Products	Selectivity (%)	Yield (%)
a	5.2	0.5
b	91.8	8.8

Conversion: 9.6%

Supplementary Figure 50 | PEC chlorination of hexane. a, GC spectrum of the products of PEC chlorination of hexane (compound **24**) on TiO_2-O_v-400 photoanode in 0.5 M NaCl electrolyte with 20 mmol hexane at 1.6 V vs. RHE under AM 1.5G, 100 mW cm^{-2} illumination for 2 h. **b**, The MS spectra of product b. **c**, ^{1}H NMR spectrum of the products of PEC chlorination of hexane. **d**, Conversion and selectivity of PEC chlorination of hexane. a.u.: arbitrary units.

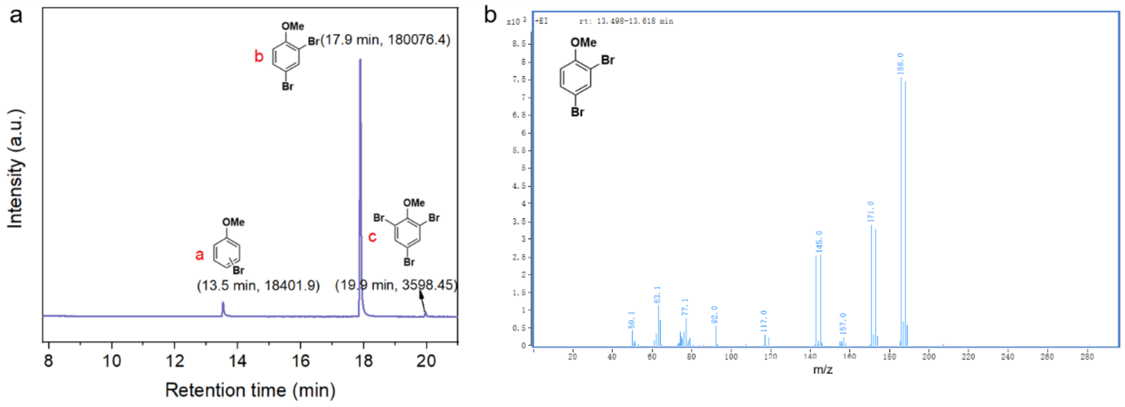

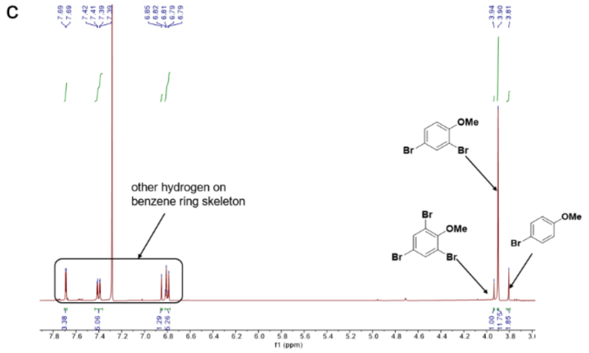

d

Products	Selectivity (%)	Yield (%)
a	8.7	8.7
b	86.2	86.2
c	1.6	1.6

Conversion: >99%

Supplementary Figure 51 | PEC bromination of anisole. a, GC spectrum of the products of PEC bromination of anisole (compound **25**) on TiO_2-O_v-400 photoanode in 0.5 M NaBr electrolyte with 0.1 mmol anisole at 1.6 V vs. RHE under AM 1.5G, 100 mW cm^{-2} illumination for 10 h. **b**, The MS spectra of product b. **c**, ^1H NMR spectrum of the products of PEC bromination of anisole. **d**, Conversion and selectivity of PEC bromination of anisole. a.u.: arbitrary units.

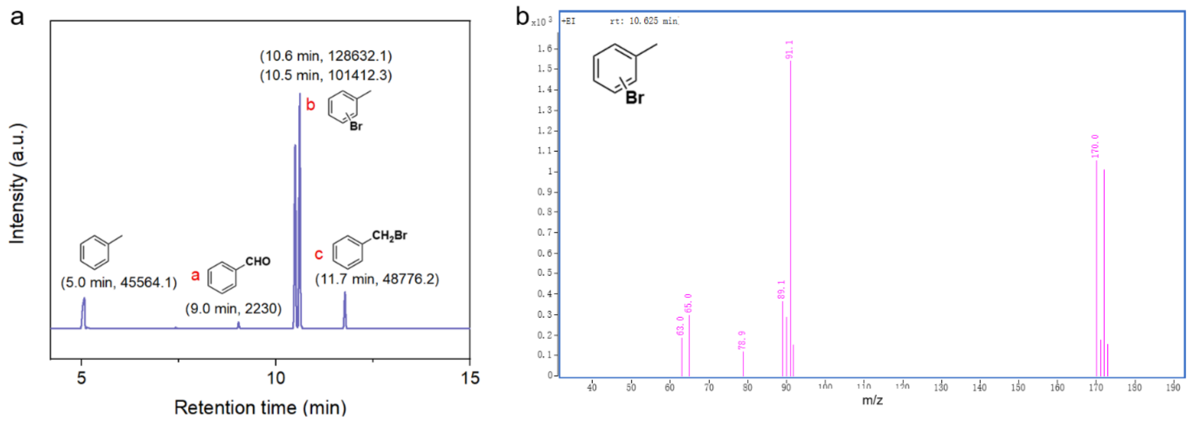

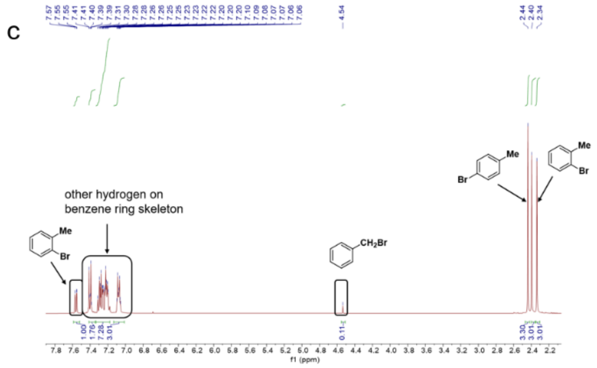

d

Products	Selectivity (%)	Yield (%)
a	0.8	0.7
b	81.2(*p*/*o*=1.1:1)	69.3
c	17.3	11.3

Conversion: 85.4%

Supplementary Figure 52 | PEC bromination of toluene. a, GC spectrum of the products of PEC bromination of toluene (compound **26**) on TiO_2-O_v-400 photoanode in 0.5 M NaBr electrolyte with 0.1 mmol toluene at 1.6 V vs. RHE under AM 1.5G, 100 mW cm^{-2} illumination for 10 h. **b**, The MS spectra of product b. **c**, ^{1}H NMR spectrum of the products of PEC bromination of toluene. **d**, Conversion and selectivity of PEC bromination of toluene. a.u.: arbitrary units.

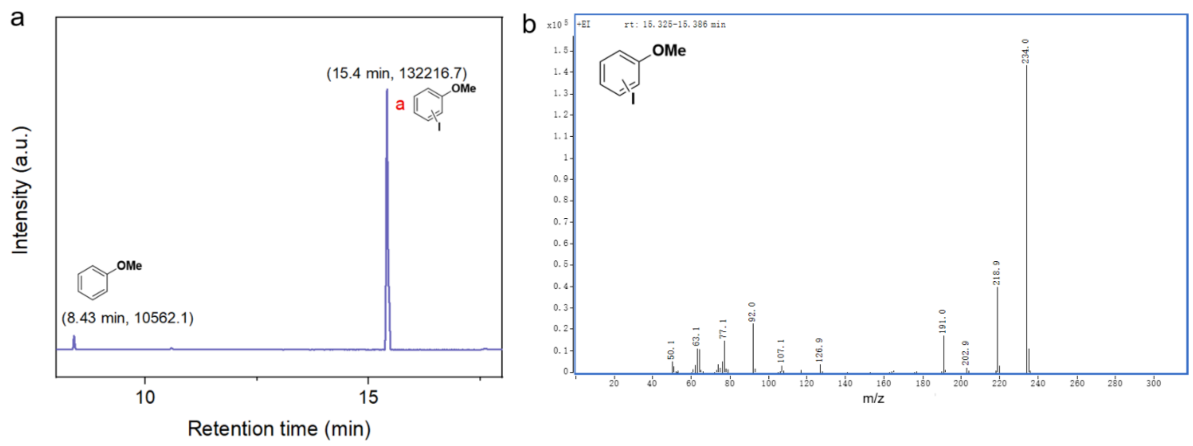

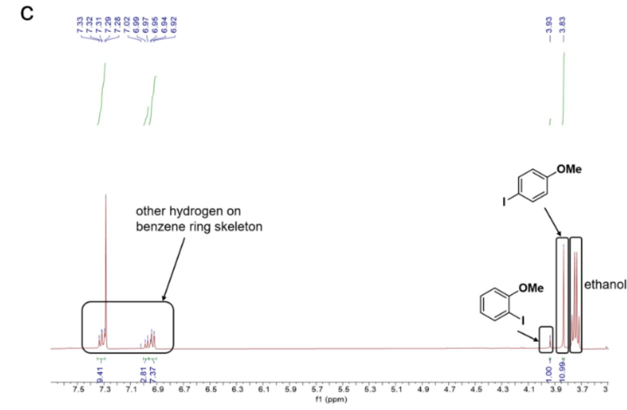

d

Products	Selectivity (%)	Yield (%)
a	>99(*p*/*o*=1:10)	90.2

Conversion: 90.2%

Supplementary Figure 53 | PEC iodination of anisole. a, GC spectrum of the products of PEC iodination of anisole (compound **27**) on TiO_2-O_v-400 photoanode in 0.5 M NaI electrolyte with 0.1 mmol anisole at 1.6 V vs. RHE under AM 1.5G, 100 mW cm^{-2} illumination for 10 h. **b**, The MS spectra of product a. **c**, ^{1}H NMR spectrum of the products of PEC iodination of anisole. **d**, Conversion and selectivity of PEC iodination of anisole. a.u.: arbitrary units.

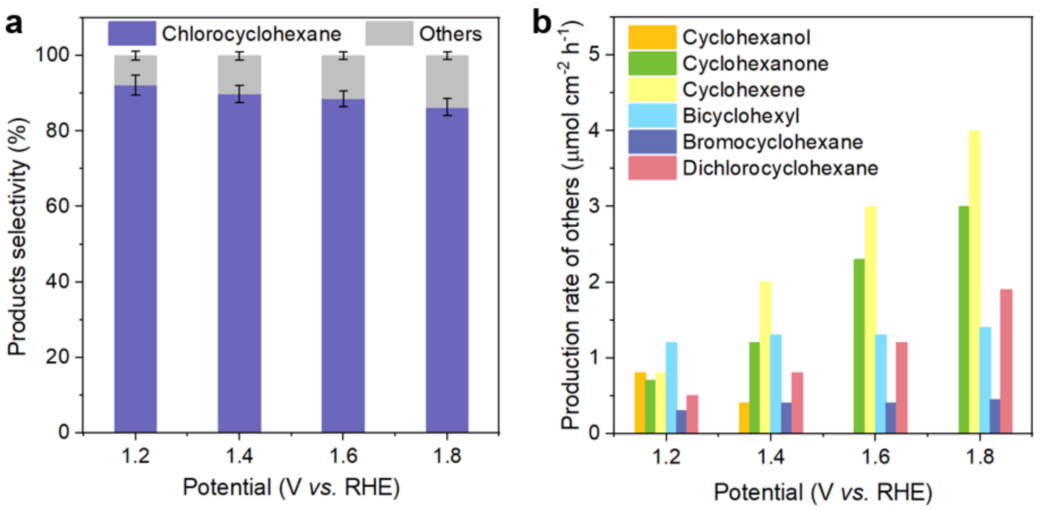

Supplementary Figure 54 | PEC chlorination of cyclohexane in sea water. a, Products selectivity of PEC cyclohexane chlorination on TiO_2-O_v-400 photoanode using sea water as electrolyte containing 18.8 mmol cyclohexane under AM 1.5G, 100 mW cm^{-2} illumination at different potentials for 2 h in the H-type cell. **b**, Production rate of other PEC cyclohexane chlorination products on TiO_2-O_v-400 photoanode using sea water as electrolyte under AM 1.5G, 100 mW cm^{-2} illumination at different potentials for 2 h in the H-type cell.

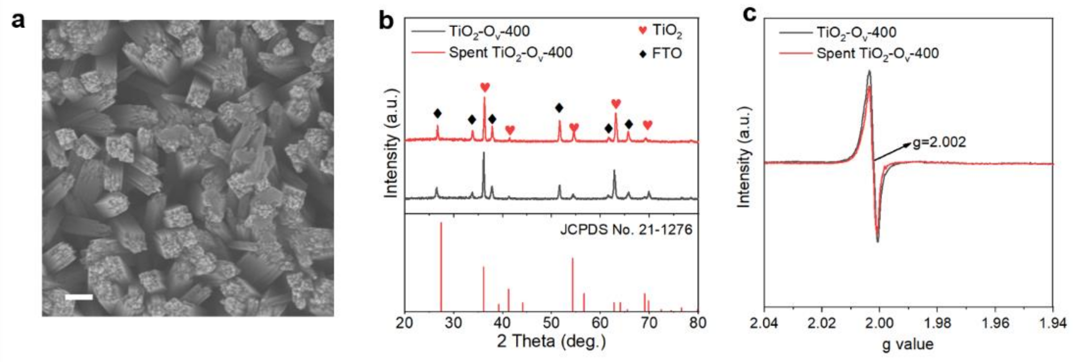

Supplementary Figure 55 | Structural characterizations of spent TiO_2-O_v-400 photoanode. a, Top-view SEM image of spent TiO_2-O_v-400 photoanode. Scale bar, 200 nm. **b**, X-ray diffraction patterns of TiO_2-O_v-400 and spent TiO_2-O_v-400 photoanodes. **c**, EPR spectra of TiO_2-O_v-400 and spent TiO_2-O_v-400 photoanodes. The spent TiO_2-O_v-400 photoanode was washed by ethanol and deionized water and dried under vacuum for further measurements.

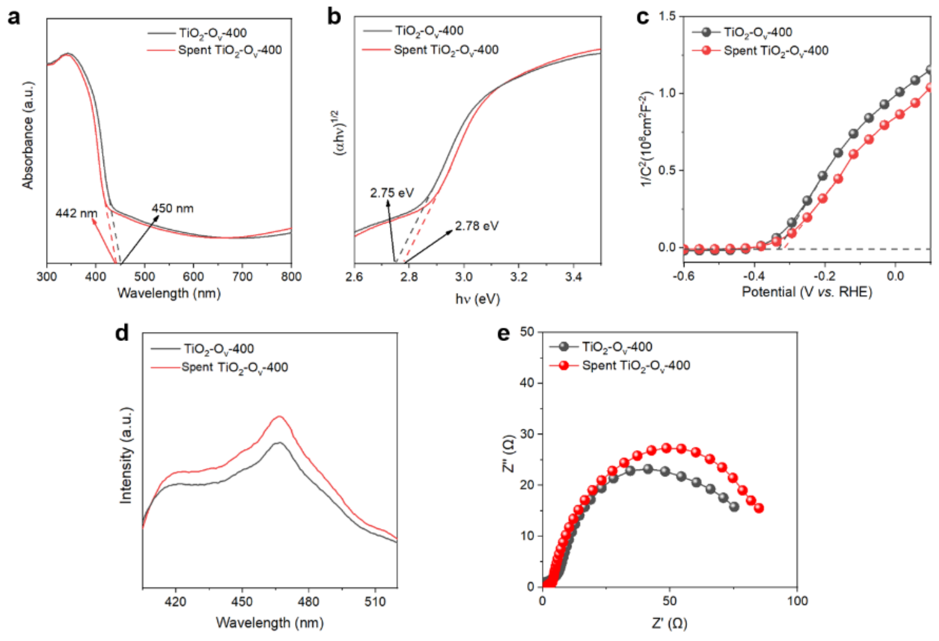

Supplementary Figure 56 | Photoelectric properties of spent TiO2-Ov-400 photoanode. a, Diffuse reflectance ultraviolet-visible spectra and the corresponding **b**, $(\alpha h\nu)^{1/2}$ versus photon energy plots of TiO2-Ov-400 and spent TiO2-Ov-400 photoanodes. **c**, Mott–Schottky plots of TiO2-Ov-400 and spent TiO2-Ov-400 photoanodes measured in 0.5 M NaCl under dark (1000 Hz). **d**, Photoluminescence spectra of TiO2-Ov-400 and spent TiO2-Ov-400 photoanodes, excitation wavelength 300 nm. **e**, The Nyquist plots of the EIS data measured under AM 1.5G, 100 mW cm^{-2} illumination.

Supplementary Tables

Supplementary Table 1. PEC conversion rate of cyclohexane, the corresponding selectivity and FE of products over TiO_2, WO_3, $BiVO_4$ and ZnO photoanodes.

Catalysts	Conversion rate (μmol cm^{-2} h^{-1})[a]	Selectivity / FE (%) [a]					FE (%)	
		chlorocyclohexane	cyclohexanol	cyclohexanone	cyclohexene	bicyclohexane	Liquid products[b]	O_2[c]
TiO_2	16.0	82.3/56.8	3.8/2.6	7.5/5.2	2.8/2.6	3.6/1.9	69.1	23.0
WO_3	6.0	86.9/65.6	2.5/1.9	5.6/4.1	2.9/2.2	2.1/1.5	75.3	20.5
$BiVO_4$	6.4	85.1/63.2	3/2.3	5.8/4.4	3.1/2.3	3/2.3	74.5	22.6
ZnO	1.6	88/68.5	2.6/2.0	5/3.9	2.6/2.0	1.8/1.4	77.8	19.7

[a]The reaction was carried out in 0.5 M NaCl electrolyte with 18.8 mmol cyclohexane at 1.6 V vs. RHE under AM 1.5G irradiation (100 mW cm^{-2}) for 2 h.
[b]Liquid products includes chlorocyclohexane, cyclohexanol, cyclohexanone, cyclohexene and bicyclohexane.
[c]Oxygen production was measured using a oxygen-sensing system.

Supplementary Table 2. The oxygen vacancy ratio of TiO_2-O_v-*T* photoanodes calculated by X-ray photoelectron spectra (XPS).

Catalysts	$O_{Vacancy}$ (eV)	$O_{Lattice}$ (eV)	$O_{Vacancy}/O_{Total}$ ratio[a]
TiO_2	532.4	530.2	0.08
TiO_2-O_v-200	532.5	530.2	0.13
TiO_2-O_v-350	532.4	530.1	0.24
TiO_2-O_v-400	532.4	530.2	0.35
TiO_2-O_v-450	532.4	530.1	0.46

[a]The ratio was calculated from the integrated area of corresponding peaks.

Supplementary Table 3. PEC conversion rate of cyclohexane, the corresponding selectivity and FE of products over different TiO_2-O_v-*T* photoanodes.

Catalysts	Conversion	Selectivity / FE (%) [a]	FE (%)

	rate (μmol cm^{-2} h^{-1})[a]	chlorocyclohexane	cyclohexanol	cyclohexanone	cyclohexene	bicyclohexane	Liquid products[b]	O_2[c]
TiO_2	16.0	82.3/56.8	3.8/2.6	7.5/5.2	2.8/2.6	3.6/1.9	69.1	23.0
TiO_2-O_v-200	34.1	85.2/62.5	3.7/2.7	4.3/3.6	2.8/2.5	4.0/2.0	73.3	19.6
TiO_2-O_v-350	52.4	89.2/71.7	-	3.6/2.9	3.1/2.8	3.5/2.4	79.8	12.6
TiO_2-O_v-400	70.0	92.5/77.5	-	1.9/1.6	1.8/1.4	3.8/3.4	83.9	7.3
TiO_2-O_v-450	42.2	92.7/79.0	-	1.8/1.5	2.6/2.2	3.9/3.3	86	7.5

[a]The reaction was carried out in 0.5 M NaCl electrolyte with 18.8 mmol cyclohexane at 1.6 V vs. RHE under AM 1.5G irradiation (100 mW cm^{-2}) for 2 h.
[b]Liquid products includes chlorocyclohexane, cyclohexanol, cyclohexanone, cyclohexene and bicyclohexane.
[c]Oxygen production was measured using a oxygen-sensing system.

Supplementary Table 4. EDS analysis of TiO_2 and TiO_2-O_v-400 after Cl^- adsorption.

Samples	Element	Weight (%)	Atomic (%)	Uncert. (%)	Detector correction	K-Factor
TiO_2-Cl	O	43.48	69.69	1.97	0.49	1.974
	Cl	0.03	0.02	0.17	0.95	1.063
	Ti	56.47	30.22	1.46	0.98	1.229
TiO_2-O_v-400-Cl	O	31.34	57.66	1.13	0.49	1.974
	Cl	0.67	0.56	0.08	0.95	1.063
	Ti	67.98	41.77	1.13	0.98	1.229

Supplementary Table 5. The amount of Cl^- remaining in the NaCl solution afterTiO_2-O_v-*T* samples adsorption.

**Samples**	**O_V/O_T ratio**	**Cl^- amount[a] (ppm)**	**Cl^- adsorption (ppm)**
NaCl	-	64	-
TiO_2	0.18	63.5	0.5
TiO_2-O_v-200	0.13	62.1	1.9
TiO_2-O_v-350	0.24	60.5	3.5
TiO_2-O_v-400	0.35	56.5	7.5
TiO_2-O_v-450	0.46	55.4	8.6

[a]The Cl^- amount was calculated from the integrated area of corresponding IC peaks.

Supplementary Note 1

The construction of models S1, S2, S3 and S4 has been described in the methods section. The chemical formulae of models S1, S2, S3, and S4 are $Ti_{32}O_{69}$, $Ti_{32}O_{69}H_8$, $Ti_{32}O_{77}H_8$, and $Ti_{32}O_{77}$, respectively. The optimized geometries of these four models in the top and side views are displayed in Supplementary Fig. 21. The transformation of S1 to S2, S3 and S4 are accomplished with eqs. 2-4:

$$S1 + 8(H^+ + e^-) \rightarrow S2 \quad (2)$$

$$S1 + 8H_2O \rightarrow S3 + 8(H^+ + e^-) \quad (3)$$

$$S1 + 8H_2O \rightarrow S4 + 16(H^+ + e^-) \quad (4)$$

Thus, the relative stabilities (defined as the ΔG for generating S2/S3/S4 from S1) of models S1, S2, S3 and S4 were calculated and displayed in Supplementary Fig. 22. As shown in Supplementary Fig. 22, S3 terminated with –OH is the most stable with the lowest Gibbs free energy under reaction potential (1.6 V vs. RHE).

Supplementary Note 2

For PEC halogenation of toluene, the products over the TiO_2-O_v-400 photoanode are the aromatic ring-chlorinated compounds with total selectivity of 77.9% at high toluene conversion (82.5%) (Supplementary Fig. 29), including *p*-chlorotoluene and *o*-chlorotoluene selectivity of 33.9 and 44.0%, respectively (ratio of *p*/*o* = 1/1.3). Benzyl chloride was also observed but with much lower selectivity (12.7%). The ratio of *p*/*o* was determined by NMR measurement (Supplementary Fig. 29c). When pure TiO_2 (with oxygen vacancy) was used as the photoanode, the main product is benzyl chloride with selectivity of 75.5% at toluene conversion of 83.2% (Supplementary Fig. 31). The above results suggest that the PEC chlorination of toluene over TiO_2-O_v-400 and pure TiO_2 may follow different mechanisms.

For PEC chlorination of toluene over TiO_2-O_v-400 photoanode that obtains *p*-chlorotoluene and *o*-chlorotoluene as the main products, we propose the reaction mainly follows an electrophilic substitution mechanism. As shown in Supplementary Fig. 32a, according to the previous reports[1], Cl^- can be oxidized by the photogenerated holes (h^+) over

photoanode to produce Cl_2. The generated Cl_2 is adsorbed on the oxygen vacancy of TiO_2-O_v-400 and polarized to form TiO_2-O_v-$Cl^{\delta-}$-$Cl^{\delta+}$ moieties due to the electron-withdrawing effect of oxygen vacancy as a Lewis acid, which was supported by the DFT calculations shown below. The charge-positive $Cl^{\delta+}$ then acts as an electrophile to attack the benzene ring of toluene to form a π-complex, while the charge-negative $Cl^{\delta-}$ remains on the oxygen vacancy to form a chlorinated anion (denoted as TiO_2-O_v-$Cl^{\delta-}$). Then, the π-complex evolves into a σ-complex, which is eventually converted to *p*-chlorotoluene or *o*-chlorotoluene by deprotonation (ratio of *p*/*o* = 1/1.3). The resulting proton reacts with the TiO_2-Ov-$Cl^{\delta-}$ anion to form HCl and the oxygen vacancy on TiO_2-O_v-400 is recovered.

The adsorption and polarization of Cl_2 on the oxygen vacancy of TiO_2-O_v-400 was demonstrated by DFT calculation. According to the HAADF-STEM image (Fig. 4c), the exposed facet of TiO_2-O_V-400 is the (101) surface. Thus, the adsorption and polarization of Cl_2 on the oxygen vacancy of the (101) surface of TiO_2-O_V-400 was investigated by DFT calculations (computational details can be found in the Methods section). By analyzing the Hirshfeld charge of the adsorbed Cl_2 (Cl_2^*) (the top and side views shown in the right column in Supplementary Fig. 23), it is revealed that the atomic charge of Cl atom adsorbed on the oxygen vacancy was calculated to be –0.06 *e*, while the atomic charge of another Cl atom in Cl_2* is 0.04 *e*, an indicative of the formation of TiO_2-O_v-$Cl^{\delta-}$-$Cl^{\delta+}$ moieties that facilitate an electrophilic substitution mechanism for PEC chlorination of toluene.

Moreover, to understand the important role of oxygen vacancy for Cl_2 adsorption and the electrophilic substitution reaction of toluene, we directly used chlorine gas (Cl_2) as the chlorine source to investigate if Cl_2 can be activated on the TiO_2-O_v to obtain similar product selectivity. We injected Cl_2 into an aqueous solution containing 5 mmol toluene in the presence of TiO_2-O_v-400 or TiO_2 or without catalyst, with the following reaction performed in darkness for 15 min. As shown in Supplementary Fig. 33a, *p*-chlorotoluene and *o*-chlorotoluene were obtained using TiO_2-O_v-400 as the photoanode demonstrating that the TiO_2-O_v with oxygen vacancy can activate and polarize Cl_2 with the following electrophilic substitution of toluene to obtain aromatic ring-chlorinated compounds. These results also reveal that Cl_2 activation over oxygen vacancy of TiO_2 can even occur under dark condition. In contrast, very small amount of aromatic ring-chlorinated products are observed over TiO_2 (blue curve) or without catalyst (black curve) under the same reaction conditions. The formation of the products may come from

the spontaneous electrophilic substitution reaction between Cl_2 and toluene but with much slower conversion than the catalytic reaction over TiO_2-O_v catalyst. These comparison results show the important role of TiO_2 with oxygen vacancy in facilitating Cl_2 activation and following toluene chlorination via an electrophilic substitution reaction.

The catalytic results of PEC chlorination of methylnaphthalene over TiO_2-O_v-400 can also be explained by the electrophilic substitution mechanism. 1-chloro-2-methylnaphthalene was observed as the main product by using TiO_2-O_v-400 as the photoanode (Supplementary Fig. 30).

For PEC halogenation of toluene over pure TiO_2 photoanode that obtains benzyl chloride as the main product, we propose that the reaction mainly follows a free-radical mechanism. As shown in Supplementary Fig. 32b, Cl^- can be activated by the photogenerated holes (h^+) over TiO_2 photoanode to chlorine radicals (Cl·) through direct single electron transfer (SET)[2]. In the reaction system, Cl_2 can be formed also by dimerization of two Cl· or by the direct oxidation of two Cl^- by h^+ over TiO_2. The generated Cl· reacts with toluene to form a carbon-centered radical via C–H bond dissociation of the methyl group on toluene. Finally, the generated carbon-centered radical reacts with Cl_2 to form benzyl chloride and release the Cl· for the next cycle.

The important role of Cl_2 for the free-radical reaction was then demonstrated by Cl_2 experiment. We injected Cl_2 into an aqueous solution containing 5 mmol toluene in the presence of TiO_2-O_v-400 or TiO_2 or without catalyst, with the following reaction performed under light irradiation for 15 min. As shown in Supplementary Fig. 33b, benzyl chloride was observed on the GC spectra for all the three reactions. This is attributed the generation of Cl· via homolytic cleavage of Cl_2 under light irradiation that doesn't require catalyst. The formed Cl· then activates C–H bond of the methyl group of toluene to form carbon radical, which is followed by the free-radical chain reaction between the carbon radical and Cl_2 to give benzyl chloride and Cl·. Therefore, we propose that the PEC halogenation of toluene over pure TiO_2 photoanode to produce benzyl chloride possibly follows a similar free-radical mechanism, in which case the Cl_2 is produced by direct oxidation of Cl^- by the photogenerated holes (h^+) over TiO_2 photoanode. Noted that *p*-chlorotoluene and *o*-chlorotoluene were also observed over TiO_2-O_v-400, although with much lower selectivity than benzyl chloride, which may be due to the occurrence of electrophilic substitution reaction of Cl_2 on oxygen vacancy of TiO_2 discussed above.

Supplementary References

1. Rassoolkhani, A. M., *et al.* Nanostructured bismuth vanadate/tungsten oxide photoanode for chlorine production with hydrogen generation at the dark cathode. *Commun. Chem.* **2**, 57 (2019).

2. Huang, C. Y., Li, J. & Li, C. J. A cross-dehydrogenative C(*sp*^{3})−H heteroarylation via photo-induced catalytic chlorine radical generation. *Nat. Commun.* **12**, 4010 (2021).
